# Analysis of the Beaufort Gyre Freshwater Content in 2003–2018

**DOI:** 10.1029/2019JC015281

**Published:** 2019-12-28

**Authors:** A. Proshutinsky, R. Krishfield, J. M. Toole, M.‐L. Timmermans, W. Williams, S. Zimmermann, M. Yamamoto‐Kawai, T. W. K. Armitage, D. Dukhovskoy, E. Golubeva, G. E. Manucharyan, G. Platov, E. Watanabe, T. Kikuchi, S. Nishino, M. Itoh, S.‐H. Kang, K.‐H. Cho, K. Tateyama, J. Zhao

**Affiliations:** ^1^ Woods Hole Oceanographic Institution Woods Hole MA USA; ^2^ Geology and Geophysics department Yale University New Haven CT USA; ^3^ Fisheries and Oceans Canada Institute of Ocean Sciences Sidney British Columbia Canada; ^4^ Graduate School of Marine Science and Technology Tokyo University of Marine Science and Technology Tokyo Japan; ^5^ Jet Propulsion Laboratory California Institute of Technology Pasadena CA USA; ^6^ Center for Ocean‐Atmospheric Prediction Studies Florida State University Tallahassee FL USA; ^7^ Institute of Computational Mathematics and Mathematical Geophysics Siberian Branch of Russian Academy of Science Novosibirsk Russia; ^8^ Laboratory of Mathematical Modeling of Atmosphere and Hydrosphere Processes Novosibirsk State University Novosibirsk Russia; ^9^ Japan Agency for Marine‐Earth Science and Technology Yokosuka Japan; ^10^ Korea Polar Research Institute Incheon Republic of Korea; ^11^ Kitami Institute of Technology Kitami, Hokkaido Japan; ^12^ Physical Oceanography Laboratory Ocean University of China, Qingdao China

**Keywords:** Beaufort Gyre, Arctic Ocean, freshwater balance, circulation, modeling, climate change

## Abstract

Hydrographic data collected from research cruises, bottom‐anchored moorings, drifting Ice‐Tethered Profilers, and satellite altimetry in the Beaufort Gyre region of the Arctic Ocean document an increase of more than 6,400 km^3^ of liquid freshwater content from 2003 to 2018: a 40% growth relative to the climatology of the 1970s. This fresh water accumulation is shown to result from persistent anticyclonic atmospheric wind forcing (1997–2018) accompanied by sea ice melt, a wind‐forced redirection of Mackenzie River discharge from predominantly eastward to westward flow, and a contribution of low salinity waters of Pacific Ocean origin via Bering Strait. Despite significant uncertainties in the different observations, this study has demonstrated the synergistic value of having multiple diverse datasets to obtain a more comprehensive understanding of Beaufort Gyre freshwater content variability. For example, Beaufort Gyre Observational System (BGOS) surveys clearly show the interannual increase in freshwater content, but without satellite or Ice‐Tethered Profiler measurements, it is not possible to resolve the seasonal cycle of freshwater content, which in fact is larger than the year‐to‐year variability, or the more subtle interannual variations.

## Key Points

- Beaufort Gyre freshwater content time series (2003–2018) from different data sets are updated, compared, and analyzed
- Qualitative and quantitative estimates of factors and mechanisms driving freshwater content changes are provided
- In 2003–2018, the major sources of accumulated fresh water were sea ice melt, Mackenzie River runoff, and Bering Strait transport

## 1 Introduction

The Arctic Ocean freshwater budget is influenced by changes in the intensity of freshwater sources associated with precipitation, including runoff from the land and exchanges with the Pacific and Atlantic Oceans, and processes governing the redistribution of the fresh water under the influence of winds, sea ice conditions (drift, concentration, and thickness), and ocean currents, as well as mixing between water masses. Many of these physical processes are influenced by changes in the vertical and horizontal distribution of ocean fresh water, with numerous interrelationships and feedback mechanisms (positive and negative). An example of the latter involves sea ice melting that forms a freshwater cap at the ocean surface, strengthening the ocean vertical stratification, and reducing heat fluxes from the ocean to sea ice, which is favorable for expanding sea ice area and increasing ice thickness (i.e., Aagaard & Carmack, 1989; Kellogg, 1975; Toole et al., 2010; Zakharov, 1981, 1997). On the other hand, loss of the freshwater cap (termed a “halocline catastrophe” by Aagaard & Carmack, 1989, and Aagaard, 1990) allowing deep convection and associated large vertical heat flux could have irreversible consequences for the climate via considerable ice melt and uncertain response of the global climate system. Another important feedback is between the Beaufort Gyre spin‐up, increased eddy activity, and stabilization of halocline properties (e.g., Davis et al., 2014; Manucharyan & Spall, 2016; Manucharyan et al., 2016; Meneghello et al., 2017; Wang et al., 2018, 2019; Zhang et al., 2016; Zhao et al., 2016, 2018). Furthermore, ecosystems depend crucially on Arctic Ocean freshwater content changes (e.g., Carmack et al., 2016).

The total climatological (here defined to be prior to 1989) liquid freshwater content of the Arctic Ocean was estimated to be around 80,000 km^3^, relative to a reference salinity of 34.8 (Aagaard & Carmack, 1989). Serreze et al. (2006) assessed that for the period 1979–2001, the Arctic Ocean liquid freshwater content was 74,000±7,400 km^3^, while Haine et al. (2015) found the figure to be around 93,000 km^3^ for 1980–2000, increasing to 101,000 km^3^ in 2000–2010. The solid fresh water stored in sea ice in 1989 was estimated to be about 17,000 km^3^ (Aagaard & Carmack, 1989) but only 10,000 km^3^ 16 years later (Serreze et al., 2006), while Haine et al. (2015) estimated the mean freshwater volume in sea ice as 14,300 km^3^ in 2000–2010. These studies indicated that over the period 2000–2010, the Arctic Ocean accumulated about 8,000 km^3^ of liquid freshwater compared to earlier decades, while the freshwater content in sea ice fell by about 2,700 km^3^. This suggests that ~5,300 km^3^ of fresh water from sources other than ice melt accumulated in the Arctic Ocean during the first decade of the 21st century. This fresh water volume is comparable to that released to the sub‐arctic seas during the Great Salinity Anomaly (GSA) episode of the 1970s (e.g., Belkin et al., 1998; Dickson et al., 1988). Thus, since the 2000s, the stage has been set for another possible release of fresh water to lower latitudes with accompanying climate impacts, including changes to sea ice conditions, ocean circulation, and ecosystems of the Sub‐Arctic similar to the influence of the GSA observed in the 1970s (e.g., Dickson et al., 2000; Greene et al., 2013; Zhang & Vallis, 2006). While the estimates of freshwater content and its changes cited above provide approximate bounds, the uncertainties in these budgets are significant due both to a lack of observational data and substantial differences in freshwater content calculation methods (e.g., estimates can differ by region considered, data quality, seasons, reference salinity, and methods of data interpolation; see Carmack et al., 2016).

**Figure 1:**
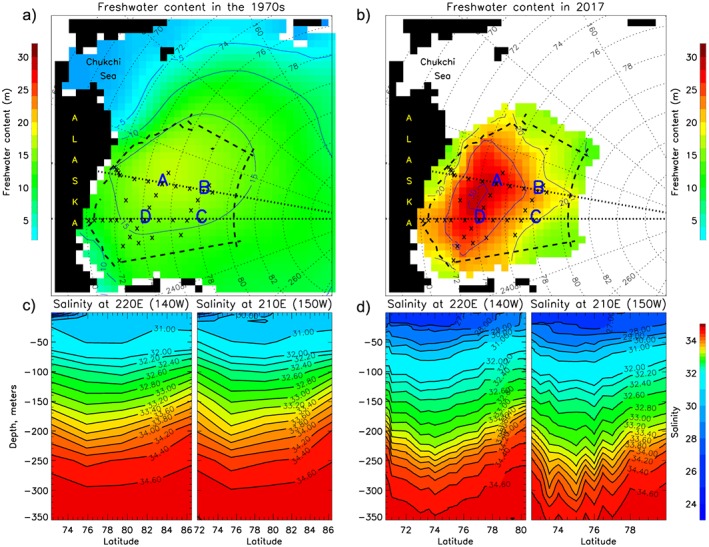
(a and b) Beaufort Gyre Observing System (BGOS) region with locations of moorings A, B, C, and D and standard CTD hydrographic survey stations (crosses). The dashed black line shows the boundary of the Beaufort Gyre region. Colors and contours are freshwater content (relative to a reference salinity of 34.8), and white grid cells indicate no data, in (a) the 1970s (from gridded climatology of Timokhov and Tanis (1998, summer atlas) at depths 0, 5, 10, 15, 25, 50, 75, 100, 150, 200, 250, 300, 400, and 500m) and (b) from optimal interpolation of 2017 hydrographic data collected from July through October and averaged every 1‐ to 500‐m depth (see section 2.1); see also Proshutinsky et al. (2019) where 2017 station locations and optimal interpolation errors are shown in supporting information S1. (c and d) Depth‐latitude sections of salinity along 140 and 150°W (thick dotted lines in panels a and b) in (c) the 1970s and (d) 2017.

The Beaufort Gyre centered in the Canada Basin of the Arctic Ocean is the major reservoir of fresh water in the Arctic (Figure 1). The primary focus of this study is on quantifying variability and trends in liquid (water) and solid (sea ice) freshwater content in the Beaufort Gyre region, defined to be between 70 and 81°N, and 130–170°W where water depths exceed 300 m (Figure 1). The Beaufort Gyre Exploration Program (BGEP) was initiated in 2003 to synthesize results of historical data analysis, design and conduct long‐term observations in the Beaufort Gyre region, and to provide information to guide numerical modeling activities (Proshutinsky, Krishfield, & Barber, 2009) under the umbrella of the FAMOS (Forum for Arctic Observing and Modeling Synthesis) project (Proshutinsky et al., 2019, preface paper in this special issue). This regional focus and our analysis based on extensive measurements from the Beaufort Gyre Observational System (BGOS; Figure 1; Krishfield et al., 2014; Proshutinsky, Krishfield, Timmermans, et al., 2009; Proshutinsky et al., 2015, 2019) to some extent limits the major uncertainties associated with Arctic‐wide measurements. However, uncertainties remain associated with limitations in measurement methods and data analysis, which are discussed below.

It is important to add that estimates of Arctic Ocean annual (1995–2010) freshwater content were made by Giles et al. (2012) based on annual sea surface height (SSH) information derived from satellite altimetry data, relative to the World Geodetic System 1984 (WGS84) ellipsoid and accounting for tidal and atmospheric geophysical corrections. They estimated a western Arctic freshwater content increase between 1995 and 2010 of 8,000±2,000 km^3^, in agreement with changes estimated by McPhee et al. (2009) and Rabe et al. (2011) based on in situ observations. Morison et al. (2012) analyzed a combination of satellite altimetry and gravimetry data from the Gravity Recovery And Climate Experiment (GRACE) to infer sources of fresh water in the Beaufort Gyre region. They put forward a hypothesis that the major source of freshwater content change in 2005–2008 was Eurasian river runoff diverted toward the Canada Basin by cyclonic winds over the Eurasian Basin associated with a low Arctic Oscillation (AO) index (Thompson & Wallace, 1998). Armitage et al. (2016) used Envisat satellite altimetry data for the period 2002–2012 and CryoSat‐2 altimetry data from 2012–2014 to estimate monthly Dynamic Ocean Topography (DOT: SSH measured by satellites relative to the geoid (e.g., Armitage et al., 2016; Farrell et al., 2012)). They combined the altimetric DOT with GRACE data and showed that together these datasets are appropriate for quantifying seasonal and interannual SSH changes, barotropic SSH changes, steric height, and freshwater content for the entire Arctic Ocean. Note that these satellite data are only available from 2003 to 2014. In the Beaufort Gyre region, Armitage et al.'s (2016) estimates of freshwater content from satellite data are in good agreement with freshwater content changes reported by Krishfield et al. (2014) based on in situ annual surveys of the Beaufort Gyre region. Carret et al. (2017) have also investigated Arctic Ocean SSH using satellite altimetry and GRACE, as well as hydrographic data from the Ocean ReAnalysis Pilot 5 (ORAP5; Zuo et al., 2015). Importantly, Carret et al. (2017) also provide analyses of Arctic sea level and freshwater content projections based on Coupled Model Intercomparison Project Phase 5 (CMIP5) simulations.

In this paper, data from the BGOS moorings and Ice‐Tethered Profilers (ITPs; Krishfield et al., 2008; Toole et al., 2011) are analyzed in context with each other and with freshwater content estimates from annual late summer‐hydrographic surveys in order to quantify Beaufort Gyre fresh water accumulation and release at different time scales (amplitudes and mechanisms) and characterize the uncertainties of the different types of measurements analyzed. In addition, satellite altimetry data corrected for ocean barotropic dynamics are used to infer freshwater content (e.g., Armitage et al., 2016; Giles et al., 2012) to quantify seasonal changes, and investigate the causes and mechanisms of fresh water accumulation and release. Each data set has its limitations. For example, effectively synoptic, full water column hydrographic surveys provide the most accurate assessment of freshwater content, however only for conditions during one season. Mooring data, on the other hand, allow for year‐round freshwater estimate, however only below 65‐m depth (the shallowest sample depth common to all BGOS moorings). Significant freshwater content changes are known to occur shallower than this in the upper ocean layer under the influence of sea ice transformations and Ekman transport convergence (Proshutinsky, Krishfield, Timmermans, et al., 2009). Freshwater content estimated from ITP data has significant uncertainty for the construction of time series and data averaging for large areas like the Beaufort Gyre region due to the Lagrangian sampling aspect of the drifting systems, the fact that ITPs typically only operate in ice covered waters, and their limited areal coverage that depends on the number of operating ITPs and their drift patterns. In addition, ITPs do not sample the uppermost (freshest) 5‐ to 7‐m ocean layer (an issue only in summer; see Proshutinsky, Krishfield, Timmermans, et al., 2009). Monthly freshwater content inferred from satellite radar altimetry has inherent uncertainties due to the empirical nature of the coefficients required to convert changes in SSH to freshwater content. These coefficients depend on ocean stratification, which differs depending on region and season. In addition, freshwater content inferred from SSH represents an integrated measure, with no information about the vertical distribution of fresh water in the water column. Finally, SSH change can only infer freshwater content change, while an estimate of the absolute volume of freshwater content requires knowledge from another data source (e.g., hydrographic surveys). While each individual data set has its limitations with respect to estimating freshwater content, the analysis of freshwater content changes combined from all of these data sources, together with the results of numerical modeling, provides better understanding of the processes and mechanisms of fresh water accumulation and release in the region.

This paper is organized as follows. First the various data sources are detailed, and the evolution of freshwater content in the Beaufort Gyre region over 2003–2018 is quantified from these different datasets. In section 3, the results are discussed in context with the major uncertainties in the freshwater content estimates. Section 4 includes a discussion of the mechanisms of fresh water accumulation and release, the sources of fresh water, and its major pathways from those source regions to the Beaufort Gyre region. Section 5 summarizes major results of this study.

## 2 Data and Methods

In sections below, each data type is described along with the particular method employed for computing freshwater content. In section 2.5 the regional atmospheric and sea‐ice forcing are described, while section 2.6 presents basic information about regional Arctic Ocean model that was employed to develop better understanding of the fresh water circulation in the region.

### 2.1 Hydrographic Surveys

Hydrographic surveys represent ship‐based CTD measurements of water temperature (T) and salinity (S). They have been conducted in the Beaufort Gyre region each year between August and October (hereafter, annual hydrographic surveys) over about 25 days at standard locations (Figure 1) during BGOS cruises. These data are available at https://www.whoi.edu/beaufortgyre and NSF's Arctic data center (https://arcticdata.io/). To this core dataset, other publically available hydrographic data in the region during summer have been added (e.g., NCEI World Ocean Database, https://www.nodc.noaa.gov/). The freshwater content at each hydrographic station has been calculated relative to a reference salinity of 34.8 and then optimally interpolated onto a 55.5‐km grid (see Proshutinsky, Krishfield, Timmermans, et al., 2009, for details of optimal interpolation method). The high‐accuracy shipboard salinity measurements result in freshwater content uncertainties for individual profiles of less than 0.1 m. Interpolation errors for the hydrographic surveys are estimated to be between 0.1 and 1.5 m depending on the number of observations and the distances between the grid points and the location of the observation sites (see Proshutinsky, Krishfield, Timmermans, et al., 2009, for details).

### 2.2 Mooring Data

**Figure 2:**
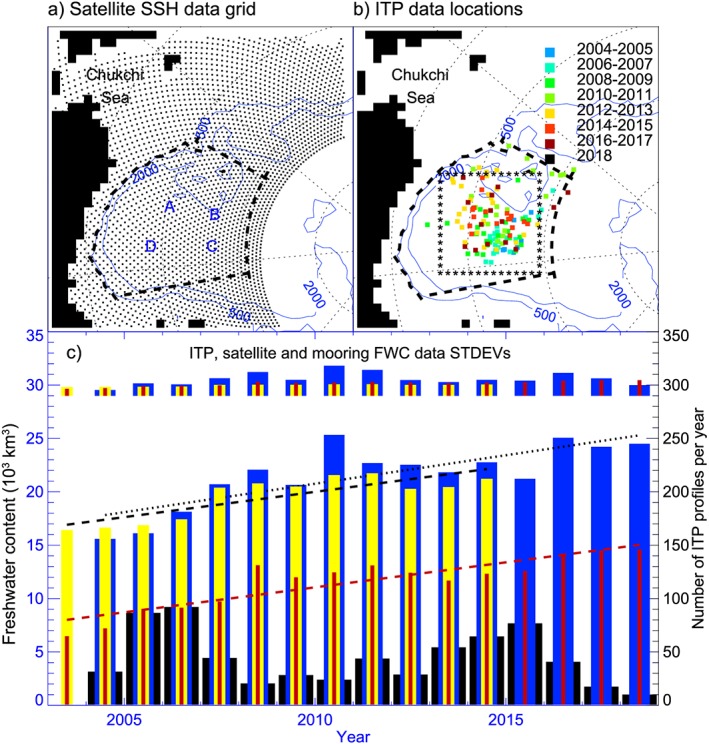
Annual freshwater content inferred from satellite data and calculated using Ice‐Tethered Profiler (ITP) and mooring data. (a) Sea surface height (SSH) grid is shown by dots. Beaufort Gyre region is bounded by the thick dashed line; mooring locations are indicated by letters A, B, C, and D. The blue contours are 500‐ and 2,000‐m isobaths. (b) Similar to the left panel but shows locations of ITP data averaged by year in 55.5‐ ×55.5‐km grid cells. Colors depict year of collected data. (c) Annual freshwater content inferred from satellite SSH data (yellow bars), calculated from ITP data (dark blue bars), and from mooring data (red bars). The black wide bars are number of ITP profiles per year in the region used for freshwater content calculations. The bars distributed along the top of the figure show the freshwater content uncertainties for each of the data sets. The black dotted, black dashed, and red dashed lines depict linear freshwater content trends from ITP (455**±**232 km^**3**^/a), SSH (524**±**256 km^**3**^/a), and mooring data (534**±**153 km^**3**^/a), respectively. Thus, all time series have similar trends (positive and significantly different from 0), and the 95% confidence intervals overlap. Note that freshwater content from moorings is calculated from a depth of 65 m to the depth of S=34.8 and it does not include the freshwater content in the upper 65 m of the water column.

Mooring data were acquired at three Beaufort Gyre locations (denoted A, B, and C in Figures 1, 2, and 6) during 2003, four locations (A, B, C, D) for the 2004–2008 period, and three sites (A, B, and D) over 2009–2018. Three types of data from the moorings are utilized here: S profiles measured by McLane Moored Profilers (MMPs), sea ice draft measured by Upward Looking Sonars (ULSs), and bottom pressure measured by Bottom Pressure Recorders (BPRs).

#### 2.2.1 MMP Data

**Figure 3:**
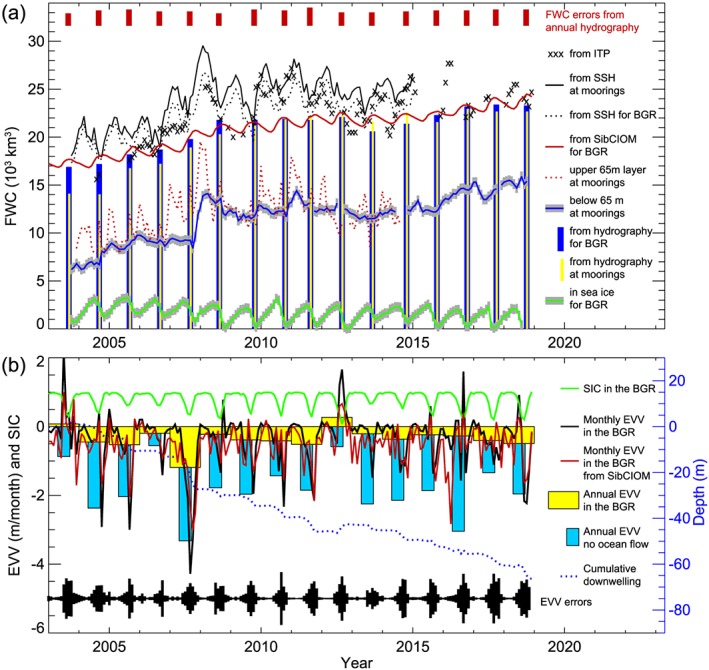
Monthly freshwater content (FWC) and Ekman vertical velocity (EVV) time series. (a) The solid and dotted black lines are FWC inferred from satellite data averaged at mooring locations and averaged for the Beaufort Gyre region (BGR; Figure 1), respectively; the black crosses are FWC from ITP data; the thick blue line with grey shading and dotted red line are FWC from mooring data below 65 m and in the upper 65‐m layer, respectively; the thick red line depicts FWC from SibCIOM model results; the blue vertical bars show FWC in the BGR from annual hydrographic surveys; the yellow vertical bars illustrate FWC from CTD casts at mooring sites before mooring deployments. The thick green line with gray shading demonstrates changes of sea ice (solid) FWC derived from ULS data with estimates of uncertainties (shading). Data uncertainties are shown as bars at the top of the panel or as shading. (b) Monthly (black line) and annual (yellow and blue bars) Ekman vertical velocity (EVV, m/month) calculated based on observations of wind, sea ice concentration and drift, and ocean currents averaged over the BGR. The thicker red line represents EVV from SibCIOM model results; the green line depicts sea ice concentration. Annual EVV calculated for motionless ocean is shown as blue bars. The blue dotted line depicts the depth of an isopycnal surface driven up or down by EVV. More precisely, this is a time integral of the EVV. The black bars depict standard EVV error.

Freshwater content at the mooring sites was calculated in the layer from 65‐m depth to the depth of the 34.8 isohaline (see Proshutinsky, Krishfield, Timmermans, et al., 2009). Note that the tops of the moorings were in all cases designed to sit deeper than 25 m to avoid collisions with ice keels, limiting the shallowest depth achievable by the profilers. Depths of the mooring tops varied on different moorings and different years, but most MMPs profiled between 65 and 2,000 m. As analyzed in Proshutinsky, Krishfield, Timmermans, et al. (2009), MMP salinity data are believed to have an uncertainty of less than 0.005, and resultant errors in freshwater content estimates (for depth intervals sampled by the MMPs) are less than 0.1 m. Freshwater content estimates were averaged in time to quantify both annual (Figure 2) and monthly changes from 2003 to 2018 (Figure 3).

#### 2.2.2 ULS Data

Sea ice draft was measured at all moorings using ULSs. The draft data are used to estimate freshwater content in the sea ice (see Proshutinsky, Krishfield, Timmermans, et al., 2009, and Krishfield et al., 2014). Note that sea ice draft changes recorded by ULSs include changes due to snow on the sea ice surface (∼0.10 m in water equivalent during seasonal cycle). It is assumed that the snow contribution to the uncertainty in our freshwater content estimates is encompassed by the estimated error in sea ice thickness (details are available in Proshutinsky, Krishfield, Timmermans, et al., 2009).

#### 2.2.3 BPR Data

Mounted on the mooring anchors, the BPRs effectively measure changes in water mass resulting from net precipitation, river influxes, and convergence/divergence associated with ocean flows. These data are used to calibrate and validate data derived from GRACE (e.g., Macrander et al., 2010; Rietbroek et al., 2006). While BPR data cannot be used for analysis of interannual and longer variability because of sensor accuracy and drift, and small changes in mooring locations each year (and therefore changes in mean bottom pressure), they are an excellent data set for the analysis of seasonal change. The pressure sensor can vary over time by 1 ppm (0.001%) of its full scale (4,136 dbar) per year. So each year the pressure sensor could vary by as much as 4 mm from its baseline due to aging of the sensor (or 0.3333 mm/month). In section 2.4, BPR data are used in conjunction with satellite radar altimetry data to investigate correlations between satellite‐derived changes in steric SSH and freshwater content in the region. For this analysis, it is assumed that atmospheric pressure changes are not sensed by the BPR; this sea‐level response is often referred to as the inverted barometer effect (sea level rise in response to reduced atmospheric pressure, and vice versa).

### 2.3 Ice‐Tethered Profiler Measurements

ITPs provide additional information on freshwater content evolution. ITPs measure water conductivity (providing S), T, and pressure from around 7‐ to 750‐m depth, with some variation depending on ice‐drift speed and other factors (see Krishfield et al., 2008, for full system and data processing details). ITP data are available at http://www.whoi.edu/itp and at https://data.nodc.noaa.gov/cgi-bin/iso?id=gov.noaa.nodc:WHOI-ITP;view=html). ITP T and S profiles from a total of 43 systems were analyzed here (Figure 2). The freshwater content in the upper waters shallower than the top of each ITP profile (typically 7‐ to 8‐m depth) was calculated assuming that the salinity at the shallowest measured layer extends to the ocean surface (see Proshutinsky, Krishfield, Timmermans, et al., 2009). Note that ITP profiles that did not extend above 10‐m depth were excluded from the analysis. In the winter, when mixed‐layer depths are consistently deeper than 10 m, this does not introduce any uncertainty. However, this approach potentially underestimates freshwater content in the upper layer during the melt season. Proshutinsky, Krishfield, Timmermans, et al. (2009) addressed this by assessment of concurrent shipboard CTD and ITP measurements and concluded that this extrapolation of ITP data during the ice melt season introduces an error in freshwater content estimates of less than 0.50 m.

### 2.4 Remote Sensing Data

Gridded DOT (SSH measured by satellites relative to the geoid) data (Figure 2) provided by Armitage et al. (2016; http://www.cpom.ucl.ac.uk/dynamic_topography/) are used to infer changes in freshwater content. In contrast to Armitage et al. (2016) who also use GRACE data in their analysis, here both GRACE data and BGOS mooring bottom pressure measurements are employed. In addition, contributions of both thermosteric and halosteric components of steric sea level changes in the Beaufort Gyre region are estimated to obtain better correlation between changes in the DOT and freshwater content. The results of our calculations are in agreement with previous studies (e.g., Armitage et al., 2016; Morison et al., 2012) showing that thermosteric corrections for the Beaufort Gyre region (even under the significant warming of the Pacific summer water layer over the previous decade described by Timmermans et al., 2018) are negligible.

Finally, we infer freshwater content in the region from satellite data using the methods suggested by Giles et al. (2012) and Morison et al. (2012). They found that the change in freshwater content **Δ** (FWC) from measurement to measurement (in our case from month to month) is approximately **Δ** (FWC) = α**Δ** (DOT_s_), where FWC is freshwater content, α is an empirical coefficient, and DOT_s_ is the steric component of DOT. In Giles et al. (2012) and Armitage et al. (2016), the α coefficient depends weakly on barotropic mass change derived from GRACE data and is on average approximately 34.5. Based on BGOS and World Ocean Data Center data for 2003–2005, Morison et al. (2012) found this coefficient to be 35.5. Our analysis using all BGOS data (2003–2014) and ITP data shows that this coefficient changes in both time and space and is on average approximately 33. The uncertainty of this coefficient is ±1.3 imparting an uncertainty of ±3.9% on our freshwater content calculations.

### 2.5 Ekman Vertical Velocities and External Forcing

External driving factors accounted for in the present analysis include geostrophic wind stress, sea ice concentration and drift, oceanic geostrophic velocities, and derived Ekman vertical velocities, all calculated using established methods. Specifically, Ekman vertical velocities are determined following the methods of Meneghello et al. (2017, 2018) and are identical to the approach published by Regan et al. (2019) in this special issue. The geostrophic wind was calculated from National Center for Atmospheric Research/National Centers for Environmental Prediction (NCAR/NCEP) (reanalysis 1; Kalnay et al., 1996) 6‐hourly sea level pressure (SLP) fields. Sea ice motion is taken from the Polar Pathfinder Daily 25‐km EASE‐Grid Sea Ice Motion Vectors, Version 4.1 (Tschudi et al., 2019). Note that errors in sea ice drift can result in substantial uncertainties in the estimates of Ekman vertical velocities derived from different satellite‐based ice drift products (Sumata et al., 2014, 2015). The ice drift curl calculated from these products reveals that the ice motion curl in winter differs by 18% on average among the different products. Daily sea ice concentration for 2003 to 2018 is from Nimbus‐7 SMMR and DMSP SSM/I‐SSMIS Passive Microwave Data Version 1 (Cavalieri et al., 1996). The ocean geostrophic velocity fields are from Armitage et al. (2017). Meneghello et al. (2017, 2018), Zhong et al. (2018), and Dewey et al. (2018) reported that after 2003, these currents played a significant role in the dynamics of the Beaufort Gyre system. For 2015–2018, we use geostrophic velocities calculated from water T and S relative to a 400‐db level of no motion from the annual hydrographic surveys.

### 2.6 Siberian Coupled Ice Ocean Model

Numerical experiments were conducted employing the Siberian Coupled Ice Ocean Model (SibCIOM), previously known as the Institute of Computational Mathematics and Mathematical Geophysics (ICMMG) 3‐D hydrostatic ocean model with Lagrangian passive tracers (floats; Golubeva & Platov, 2009). This model was employed to examine freshwater transport in the Arctic Ocean from 1985 to 2016. The ocean model is based on the traditional equations of ocean dynamics and thermodynamics in curvilinear orthogonal coordinates using the hydrostatic and Boussinesq approximations. A rigid‐lid approximation is used at the ocean surface. After vertical integration of the equations of fluid motion, the integral component of the motion (barotropic mode) and the deviation from it (baroclinic mode) are distinguished. For the barotropic mode, the equations of motion reduce to the equation for the integral stream function; a numerical solution is described in more detail by Golubeva and Platov (2009). Integration of the ocean module is carried out together with the CICE v3 model of Hunke and Dukowicz (1997).

Heat and humidity fluxes, wind stress, and downward longwave and shortwave radiation fluxes were taken from NCEP/NCAR reanalysis data (Kalnay et al., 1996). The initial temperature and salinity fields were specified using the climatic data of the Polar science center Hydrographic Climatology for winter (Steele et al., 2001).

The model's grid is configured from 20°S in the Atlantic Ocean to 60°N in the Pacific Ocean (Golubeva & Platov, 2007). The horizontal computational grid is bipolar curvilinear and has an equatorial resolution of 0.5° (minimal spacing is ∼19 km in the study region). In the vertical direction, the grid consists of 38 levels with a maximum resolution of 5 m in the upper 20‐m layer. The minimum depth on the shelf is 20 m. At the grid nodes where the bottom depth was less than 20 m, the depth was artificially deepened to 20 m. Some straits of the Canadian Arctic Archipelago were artificially expanded according to the model resolution.

The model takes into account the inflow from the 52 largest rivers in the domain. Data on the average seasonal runoff of these rivers were obtained from measurements of hydrological stations (Vörösmarty et al., 1998). The model's particle motion algorithm is provided in the supporting information (S1). This model has been used effectively in AOMIP and FAMOS model intercomparison studies (e.g., Aksenov et al., 2016; Dukhovskoy et al., 2016; Timmermans et al., 2014) and has demonstrated good results.

## 3 Freshwater Content Variability

### 3.1 Interannual Changes

Based on the annual hydrographic surveys, the Beaufort Gyre region accumulated approximately 6,400 km^3^ of fresh water (relative to 34.8 reference salinity) during 16 years between 2003 and 2018 (Proshutinsky et al., 2019; this special issue). This represents a 40% increase of fresh water volume in the region relative to the climatology of the 1970s. While the average rate of fresh water accumulation was 397±116 km^3^/a, the freshwater content growth was not uniform due to changes in wind, sea ice conditions, and ocean geostrophic currents. The ocean circulation played a flywheel role regulating momentum transfer from wind to the ocean (moderated by sea ice conditions), effectively damping disturbances in external forcing and stabilizing freshwater content (e.g., Dewey et al., 2018; Doddridge et al., 2019; Meneghello et al., 2017, 2018; Zhong et al., 2018).

In 2003–2008, freshwater content measured in August–October increased by 4,900±1.550 km^3^, from 16,900±1,400 km^3^ in 2003 to 21,800±1,700 km^3^ in 2008. Freshwater content then remained stable at around 22,000±1,900 km^3^ for 4 years. In 2013, freshwater content decreased by about 1,600±500 km^3^ almost to the volume observed in 2007. In 2014–2016, freshwater content again increased to reach a historic maximum of around 23,200±1,800 km^3^ in 2016. In 2017 and 2018, the freshwater content changed only modestly to 23,400±1,800 km^3^ and 23,300±2,000 km^3^, respectively. As these estimates are based on the annual hydrographic observations, they do not resolve the seasonal cycle.

Annual mean freshwater content integrated over the Beaufort Gyre region derived from satellite‐based data, ITPs, and MMPs (Figure 2) is in general agreement with the freshwater content volume in the region calculated from annual hydrographic survey data and the wind forcing (see also Figure 3). There are high correlations between the annual time series calculated from moorings, satellites, and ITP data (Figure 2). Before computing the correlations, the time series were detrended and first‐differenced (following usual practice to remove autocorrelation in the time series, e.g., Emery & Thomson, 2001). For every correlation coefficient estimate, a statistical *t* test was completed for the null hypothesis of 0 correlation (if *p*‐value <0.05, the null hypothesis is rejected; confidence level is 0.1). All correlations were significant except for the satellite‐based and ITP data time series, due to the ITP‐based time series being too sparse in time and therefore too short in duration. The maximum correlation coefficient is 0.77 with confidence intervals [0.419; 0.918] between mooring and satellite‐based annual freshwater contents, and the minimum is 0.32 [−0.235; 0.712] between ITP and mooring data time series. It is noteworthy that freshwater content inferred from the moorings and satellite data are highly correlated even though moorings do not sample the upper 65 m (i.e., the mixed layer and the top of the Pacific layer water column freshwater content).

The annual trend of freshwater content from the mooring data (534±152 km^3^/a) agrees within uncertainty bounds with the trend obtained from the annual hydrographic surveys (422±130 km^3^/a shown and discussed in Proshutinsky et al., 2019). The linear trend of freshwater content derived from ITP data (455±232 km^3^/a) also agrees within statistical uncertainty to the trends estimated from the mooring data and hydrographic surveys. The satellite inferred freshwater content trend (524±256 km^3^/a) is very close to the trends of fresh water accumulation estimated from the ITP and hydrographic measurements.

Comparison of freshwater content derived from in situ T and S hydrographic measurements with freshwater content from altimetry for those months that coincide with the hydrographic surveys reveals good correlation (0.80 [0.612; 0.950]) between these two time series (Figure 3). Note that statistics and time series of steric SSH are essentially the same whether GRACE or BPR data are used to convert altimetric DOT to steric SSH change.

Based on these results, it is postulated that over the 2003–2018 period mooring‐based estimates of freshwater content changes accurately characterize the accumulation of fresh water in the region, but we admit that there is not enough information about freshwater content in the upper 65 m and that the ITP regionally averaged data have significant uncertainties discussed above. The second important conclusion from this analysis is that the rate of fresh water accumulation below 65‐m depth is in agreement with the rate of freshwater content for the entire water column.

### 3.2 Seasonal Changes

**Figure 4:**
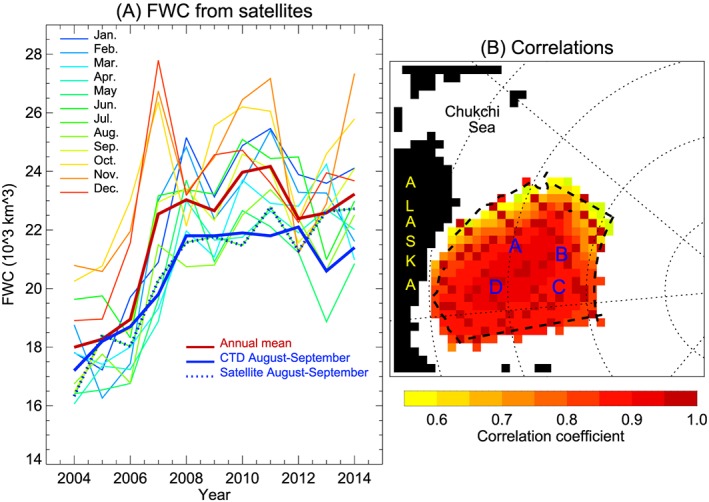
(a) Freshwater content changes in the Beaufort Gyre region inferred from satellite data in 2004–2014 by month. The thin colored lines show the interannual variations by month, while the annual mean is depicted by the thick red line. The thick blue line shows the evolution of freshwater content observed in August–September. This is identical to freshwater content shown by the thin yellow bars in Figure 3a. The dotted blue line represents August–September freshwater content inferred from satellite data. (b) Correlation between time series of freshwater content in each grid point and the mean freshwater content in the Beaufort Gyre region. High correlation at mooring locations indicates that observations at these locations are representative of estimates of freshwater content in the whole region.

It is important to be aware that the estimates of freshwater content discussed above are for the August–October season. Satellite‐based data show that the range of monthly freshwater content changes is greater than that based solely on the annual hydrographic surveys. Analyzing the monthly freshwater content time series for 2004–2014 inferred from satellite data (Table 1 and Figure 4), it is found that the maximum freshwater content occurs in December and the minimum in April, and the maximum interannual trend (710 km^3^/a) was observed in January and minimum trend (360 km^3^/a) in December. The maximum difference between highest and lowest freshwater content in the region was observed in February (9,140 km^3^, comparing 2011 and 2004). These results also demonstrate that regional fresh water accumulation and release has been occurring continuously in response to changes in the wind forcing, sea ice conditions, and ocean circulation. The observations indicate a lag of approximately 2 months between stress curl at the ocean surface and freshwater content in the region (based on data shown in Figure 3).

**Table 1:** Statistics of 2004–2014 FWC Inferred From Satellite Data

Month	Trend ×10^3^km^3^/a	Mean ×10^3^ km^3^	SD ×10^3^ km^3^	Maximum ×10^3^ km^3^	Minimum ×10^3^ km^3^	Change ×10^3^ km^3^
January	0.71	22.35	2.96	25.46	17.22	8.24
February	0.58	21.80	3.01	25.40	16.26	9.14
March	0.60	20.93	2.46	24.24	17.41	6.83
April	0.65	20.47	2.56	23.00	16.06	6.94
May	0.44	19.97	2.55	23.70	16.41	7.29
June	0.40	22.32	2.27	25.09	18.32	6.77
July	0.58	20.66	2.16	22.78	16.32	6.46
August	0.57	20.52	2.38	23.38	16.74	6.63
September	0.62	21.72	2.73	24.59	16.46	8.13
October	0.40	23.92	2.29	26.37	20.25	6.12
November	0.40	23.90	2.66	27.34	20.58	6.75
December	0.36	23.02	2.57	27.79	18.91	8.88

The rate of fresh water accumulation below 65‐m depth is in agreement with the rate of freshwater content for the entire water column. We speculate that the Pacific water layer in the Beaufort Gyre region receives fresh water directly from waters entering through Bering Strait, and modified by surface buoyancy forcing in the Chukchi Sea, that are denser than the surface waters of the Canada Basin. Prevailing winds and ocean geostrophic flow drive these Pacific waters into the Canada Basin where they ventilate the Beaufort Gyre region halocline (Timmermans et al., 2014, 2018). This explanation is in agreement with seasonal changes of freshwater content below 65 m observed in the mooring data where maximum freshwater content was observed in January–February and minimum in June–July when wind‐driven downwelling in the southwest Canada Basin/Chukchi Sea is replaced by upwelling. Zhong et al. (2019, this special issue) investigated changes in the Pacific winter water located between the Pacific summer and Atlantic water layers and showed that under the influence of Ekman pumping and lateral advection, the thickness and freshwater content of this layer increased by approximately 18% over the years 2002–2016.

**Figure 5:**
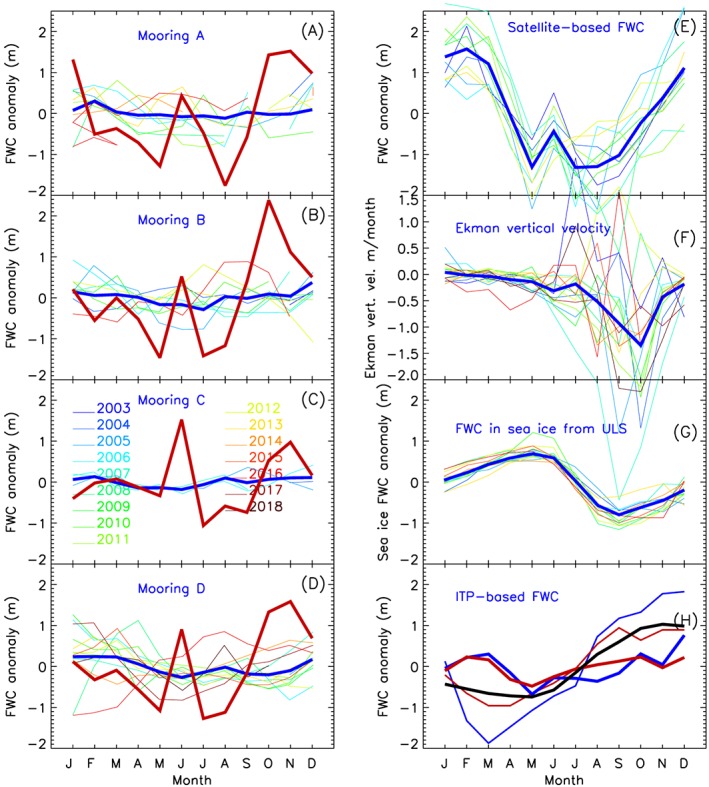
Seasonal anomalies of freshwater content (FWC) and external forcing. The thin lines depict individual years; the thick lines represent the mean annual cycle for the period 2003–2018. (a–d) FWC below 65 m (thin lines and thick blue lines) and in the upper 65‐m layer (thick red line) at moorings A, B, C, and D. Note that the upper 65‐m layer FWC is calculated as the difference between FWC inferred from satellite SSH data at the mooring locations and FWC based on mooring observations below 65 m. (e) FWC inferred from SSH satellite‐based data at mooring locations. (f) Monthly anomalies of Ekman vertical velocities in the rectangular region (RR) are shown. (g) FWC in sea ice at mooring locations from ULS dat. (h) FWC derived from ITPs drifting in the RR (see Figure 2b). In panel H, the thinner red and blue lines depict FWC from ITP data obtained in 2007 for the upper 65‐m water column and for the total water column in the RR, respectively. The thick red and blue lines show the same as thin lines but for the period 2003–2018. The thick black line depicts freshwater content from the SibCIOM model output in the RR.

Analysis of the freshwater content seasonal cycle indicates significant variability in phase and magnitude from year to year (Figures 3, 4, and 5). Proshutinsky, Krishfield, Timmermans, et al. (2009) analysis of 2003–2008 mooring and ITP data revealed that the seasonal cycle of liquid freshwater content during this period was not consistent with the conventional understanding of the Arctic Ocean hydrological cycle in which liquid freshwater content is maximum in August and minimum in April (Steele et al., 2001; Serreze et al., 2006; Timokhov & Tanis, 1997, 1998). Instead, the observations indicated that over 2003–2008, there were two seasonal maxima in freshwater content; one in June–July when sea ice thickness reached its minimum (maximum ice melt) and the second in November–January when Ekman pumping was strongest and salt input from ice growth had not yet reached its maximum. Furthermore, the timing and phase of the seasonal cycle were different depending on location (details are available in Proshutinsky, Krishfield, Timmermans, et al., 2009). Note that in that analysis, the seasonal cycle of freshwater contents from the moorings (below 65 m) were extended to the surface using ITP data, with attendant uncertainties (see Proshutinsky, Krishfield, Timmermans, et al., 2009).

The data inferred from satellites also allows us to estimate how well BGOS observations can manifest freshwater changes. Correlating freshwater content changes in each cell of our 55.5‐km grid (see section 2.1.) with changes of regional mean freshwater content reveals that the data from all moorings are representative (Figure 4) because in the center of the region, correlations are greater than 0.95.

To analyze the seasonal cycle of freshwater content at the mooring sites over 2003–2018, freshwater content is expressed in terms of meters of water at the point locations instead of thousands of cubic kilometers for the region as discussed in previous sections. In general, freshwater content below the mixed layer at the mooring sites (deeper than 65 m; Figures 5a and 5b) shows small seasonal change, with the maximum observed in January–February and minimum in June–July, depending on location. The range of the seasonal change of freshwater content below 65 m is about 0.5 m on average but can be up to 1.5 m in some years at moorings B and D (Figures 5a and 5b, thin colored lines).

The seasonal cycle of freshwater content (at the mooring locations) inferred from the satellite data (that includes contributions from the mixed layer; Figure 5e) reveals a primary maximum in February, a minimum in May, and a secondary well‐pronounced maximum in June. Freshwater content then attains a minimum in July, followed by a gradual increase to February.

Similar freshwater content inferred from satellite data was reported by Armitage et al. (2016) and is also discussed by Regan et al. (2019, this special issue). While Proshutinsky, Krishfield, Timmermans, et al. (2009) and Armitage et al. (2016) explain the freshwater content maximum in June as the result of interplay between Ekman convergence strength and the availability of freshwater from sea ice, Regan et al. (2019) argue that the distribution and strength of seasonal atmospheric forcing may be responsible for this June maximum. This explanation is effectively the same as that put forward by Proshutinsky, Krishfield, Timmermans, et al. (2009) and Armitage et al. (2016) since Ekman convergence strength and sea ice conditions (thickness, drift, and concentration) are regulated by atmospheric winds driven by gradients of the Beaufort High atmospheric pressure.

It is revealing that freshwater content inferred from mooring data (i.e., only including the contribution deeper than 65 m) does not have a maximum in June. Therefore, the June maximum must be a manifestation of changes in the upper 65 m of the ocean. To examine this, monthly freshwater content in the upper 65‐m layer was calculated as the difference between the full water column freshwater content (inferred from satellite data at mooring locations) and mooring freshwater content below 65 m. The resulting freshwater content seasonal cycle in the upper 65 m is effectively the same as the seasonal cycle of freshwater content inferred from the satellite data because the seasonal cycle is confined to the upper 65 m (Figures 5a and 5b, thick red lines). In June, the central part of the Beaufort Gyre receives between 1.5 and 2 m of fresh water, while the following month in July, the fresh water returns to low pre‐June values. Note that this distinct seasonal signal in freshwater content is only inferred from satellite data (and not recorded by ITP data, described next). Further explanation will be provided in the next section.

Freshwater content calculated from ITPs was averaged monthly over the Beaufort Gyre region to investigate seasonal changes (Figures 2 and 3). While monthly changes in freshwater content calculated from the ITP data can be up to 5 m (Figure 3), these estimates may be influenced by ITP irregularity in the temporal and spatial sampling within the Beaufort Gyre region (Figure 2). The mean seasonal cycle of freshwater content derived from ITP data is relatively simple (Figure 5h). It has just one minimum in May and one maximum in September. Interestingly, the freshwater content seasonal cycle from ITP data is in excellent agreement (anticorrelated) with the seasonal cycle of freshwater content in sea ice, which probably reflects the fact that ITPs rarely sampled ice free waters.

### 3.3 Some Uncertainties and Complications

It is not possible without additional information to fully explain the June peak in satellite‐derived freshwater content (Figure 5e), which is not replicated in freshwater content time series inferred from other data sources (Figure 5). It is possible that the discrepancy is due to problems in the SSH estimates unique to this month of the year: for instance, resulting from either melt pond formation and/or rapid freshwater injection from rivers. Armitage et al. (2016) attempted to account for the effect of melt‐pond formation in the summer on estimated SSH using a seasonally varying correction, but there may be unresolved increases in SSH associated with the formation of melt ponds in June. Further, this effect might be expected to be spatially varying and depend on the type of sea ice present, as it is known the extent and height of melt ponds depends to a great extent on sea ice deformation. This might also explain the SSH drop in July when the fresh water from melt ponds drains into the ocean.

Importantly, fresh water release from melt ponds to the ocean is not measured by GRACE and BPRs, and indeed, the GRACE analysis reported by Armitage et al. (2016, Figure 5) and Peralta‐Ferriz and Morison (2010, Figure 2b) show only small peaks in June. Hereafter this will be cited as the A‐P‐F&M effect. BGOS BPR measurements (not shown) indicate a small increase in bottom pressure in June or/and July depending on the year, which is in general agreement with the A‐P‐F&M result although the BPR peak is smaller in magnitude. The differences in magnitude and phase in BGOS BPR data and in A‐P‐F&M are probably due to the different geographic areas investigated with the GRACE and BPR data. Only information for the Beaufort Gyre region is considered in this study, while A‐P‐F&M analyze data from all Arctic basins.

On the other hand, the barotropic SSH peak in June (Armitage et al., 2016) coincides with a peak in the GRACE seasonal cycle, which could be due to the rapid thawing and outflow of rivers (Peralta‐Ferriz & Morison, 2010). This may not be fully accounted for in the steric sea level estimates used to calculate freshwater content. Note that in order to infer freshwater content changes from DOT data, all barotropic SSH changes related to the ocean mass change must be removed. This can be done by removing SSH inferred from GRACE or BPRs from the DOT data. Without this correction, the calculated freshwater content will have an error proportional to the barotropic SSH anomaly multiplied by 33 (see section 2.4; α coefficient). In this sense, an error of 2 to 3 cm in the estimation of barotropic component of SSH will result in 60 to 100 cm of freshwater content bias.

## 4 Discussion

### 4.1 Mechanisms of Fresh Water Accumulation and Release

Three major causes of freshwater content change observed in the Beaufort Gyre region at different timescales have been suggested (Proshutinsky et al., 2002, Proshutinsky, Krishfield, & Barber, 2009; Proshutinsky, Krishfield, Timmermans, et al., 2009), namely, (a) wind‐generated Ekman transport convergence and pumping, which drive the mechanical redistribution of fresh water in the region including fresh water accumulation from outside the study region (such as river runoff and other sources of fresh water discussed above); (b) seasonal and interannual ice melt and growth accompanied by salt exchange between ocean and sea ice; and (c) ocean mixing and changes in ocean stratification. Each is discussed in turn below.

#### 4.1.1 Ekman Pumping

Seasonal changes of wind forcing modified by sea ice conditions and ocean circulation are analyzed here in terms of Ekman vertical velocity anomalies (Figure 5f with negative velocities corresponding to downwelling). Despite significant wind stress curl at the ice surface from December to May, the downwelling increases only slowly at this time because internal ice forces reduce ice motion and inhibit momentum transfer from wind to the ocean surface. These well‐known effects (Hibler, 1979) are typically taken into account directly in numerical models, and also in bulk estimations of stress curl at the ocean surface using observed sea ice motion (e.g., Ma et al., 2017; Yang, 2006, 2009; Zhong et al., 2018). An additional influencing factor on interannual change in Ekman vertical velocity is related to the Beaufort Gyre flywheel effect discussed by Proshutinsky et al. (2002) and is usually included in ice‐ocean modeling studies. The flywheel effect refers to the sustained anticyclonic ocean flow that at times results in the ocean moving faster than the sea ice (i.e., in winter when the expansive sea‐ice pack restricts ice drift or when atmospheric forcing shifts to weakly cyclonic circulation in summer). Several recent studies that have included geostrophic currents in calculations of ice‐ocean stress curl show that stress curl at the ocean surface can be overestimated when geostrophic currents are not accounted for (Dewey et al., 2018; Meneghello et al., 2017, 2018; Zhong et al., 2018).

With the seasonal reduction of sea ice concentration beginning in July and lasting until October–November, sea ice responds more readily to anticyclonic wind forcing, and the upwelling effect associated with the anticyclonic ocean flow becomes less important. Over this period, the magnitude of stress curl at the ocean surface increases, leading to maximum downwelling in October (Figure 5f). This interplay between wind forcing, ice motion, oceanic circulation, and eddies (dissipating available potential energy in the gyre) in the Beaufort Gyre region was recently named “the ice‐ocean governor” (a reference to the regulating effect of sea‐ice on the wind‐driven spin‐up of the gyre), described and tested via theoretical and idealized modeling (Doddridge et al., 2019; Meneghello et al., 2018; Wang et al., 2019).

**Figure 6:**
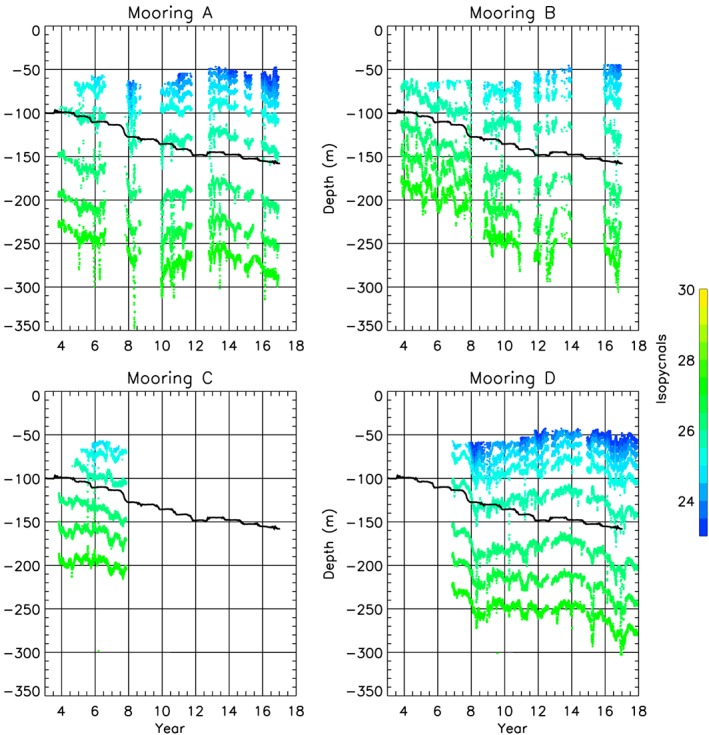
2003–2018 downwelling observed at Beaufort Gyre Observational System (BGOS) moorings A, B, C, and D. Isopycnals (colored dots) are plotted in depth − time (year + 2000) coordinates to illustrate the rate of observed downwelling. The thick black line indicates that the depth an isopycnal would have as a result of the cumulative effects of the Ekman vertical velocity over the Beaufort Gyre region (see also Figure 3b).

Our calculations of Ekman vertical velocity are substantiated by comparison with observed isopycnal displacements at the mooring locations (Figure 6). Cumulative Ekman‐driven downwelling and deepening of the isopycnals at the moorings (Figure 6) are generally in agreement until around 2008–2010. Significant increases in Ekman pumping were observed in 2007–2008 (Figure 3b), particularly well pronounced at mooring D. After 2008, some stabilization of isopycnal depths is observed (and even upwelling at moorings A and D) until around 2014. After 2014, all mooring records again show downwelling in agreement with Ekman pumping. This isopycnal behavior is in broad agreement with freshwater content variability in 2003–2018 (Figure 3).

**Figure 7:**
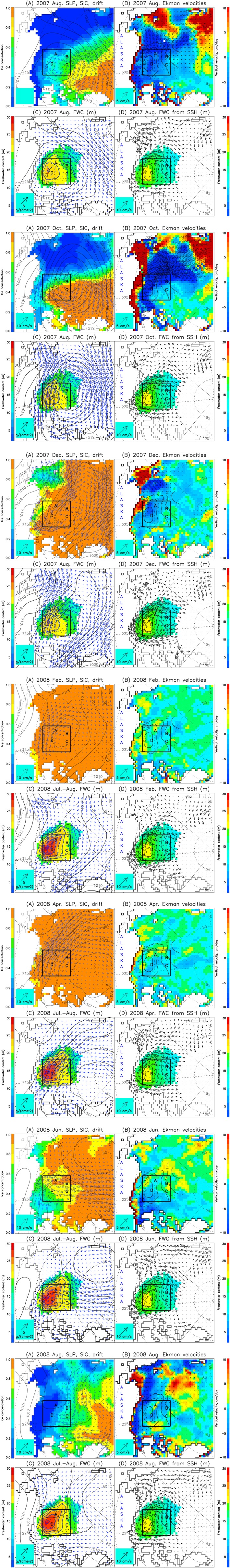
(a) Monthly freshwater content and forcing factors in the Beaufort Gyre region in August 2007. (A) Sea ice concentration (colors), ice drift (vectors, cm/s) where ice was present. The black contours show sea level pressure (SLP; hPa). (B) Ekman velocities. Horizontal Ekman mean velocities are depicted by vectors (cm/s) and Ekman vertical velocity (cm/day) by colors. The black solid lines represent freshwater content (m) with 2‐m increment. (C) freshwater content from CTD data is shown in colors and by blue contours (m, 2‐m increment); Vectors show wind stresses at the ice and ocean surface (g · cm^−**1**^ · s^−**2**^); (D) August freshwater content (m, colors and contours) inferred from DOT. Vectors depict geostrophic currents inferred from DOT and published by Armitage et al. (2017). Note that we intentionally repeat freshwater content observed in the annual hydrographic survey (panels C in all Figures 7) to better visualize the rate of fresh water accumulation and accurately compare observed freshwater content in August with freshwater content inferred from the monthly satellite data. (**b)** Same as in Figure 7a but for October 2007. **(c)** Same as in Figure 7a but for December 2007. (**d)** Same as in Figure 7a but for February 2008. (**e)** Same as in Figure 7a but for April 2008. (**f)** Same as in Figure 7a but for June 2008. (**g)** Same as in Figure 7a but for August 2008.

The process of fresh water accumulation in 2007–2008 for the Beaufort Gyre region is illustrated by Figure 7 (see also Figure S1 in supporting materials), which shows forcing factors and calculated Ekman horizontal and vertical velocities. In 2007 and 2008, the Beaufort Gyre region accumulated more than 2,000 km^3^ of fresh water (30% of the accumulation observed over the 2003 to 2018 period). In August 2007, the Beaufort High (atmospheric pressure maximum in the region) was unusually strong (in contrast to the typical climatological August low pressure system). The associated strong anticyclonic winds (Figure 7a, panel C) drove sea ice to drift anticyclonically much faster than usual (Figure 7a, panel A). This resulted in intensive Ekman transport convergence in the ocean with maximum Ekman pumping (between 5 and 10 cm/day in the central part of the Beaufort Gyre region). Note that the distributions of freshwater content observed during the August hydrographic survey and inferred from satellite data are in excellent agreement (Figure 7a, panels C and D). Further, geostrophic currents (Figure 7a, panel D; Armitage et al., 2017) agree with the wind and ice forcing, and freshwater content distribution. In September and October (Figures 7b and S1a, respectively), fresh water accumulation intensified (sea ice concentration continued to decrease, while wind, ice drift, and geostrophic currents were increasing).

By December 2007 (Figure 7c), due to the increase of sea ice extent and concentration (and resulting intensification of internal ice forces), the seasonal cycle of fresh water accumulation was completed and freshwater content and geostrophic circulation reached seasonal maxima, while Ekman pumping started to decrease (compare Figures 7b–7d, panels B and D). From January (Figure S1c) through April 2008 (7e), despite sustained anticyclonic winds and ice drift, the wind‐driven momentum transfer to the ocean was moderated by strong internal ice stresses. Ekman transport divergence and upwelling ensued when the ice drift became comparable to, or smaller than, the geostrophic ocean currents (compare panels A and D in Figures 7d–7f). See also results of Meneghello et al. (2017, 2018), Zhong et al. (2018), and Dewey et al. (2018) studies. In June–July, the freshwater content in the region slowly decreased and reached a minimum around July–August (Figures 7f and S1f, panels C and D). From June to August, due to reduced sea ice cover and a continuation of anticyclonic wind forcing in 2008, vertical Ekman velocities began increasing and in August the observed (Figures 7g and S1g, panels C) and inferred freshwater content distribution from satellite data (Figures 7g and S1g, panels D) became practically identical.

It is important to note that it would not be possible to understand and explain these dynamics of fresh water accumulation and release by investigating freshwater content changes in the region using only the annual hydrographic survey data. The freshwater content between August 2007 and August 2008 (Figure 3, vertical blue bars) increased by 2,000 km^3^, but the estimated magnitude of freshwater content in the seasonal cycle of 2007–2008 reached more than 5,000 km^3^ (a result well supported by the estimates inferred from the satellite data and calculated from ITP measurements; Figure 3, top panel, black lines and crosses). We also suspect that due to significant changes of freshwater content and upper ocean stratification through the seasonal cycle, the processes of mixing and eddy dynamics also have strong seasonality, but assessment of these processes is beyond the scope of this paper.

#### 4.1.2 Fresh Water Availability and Freshwater Content Balances

Proshutinsky et al. (2002), Proshutinsky, Krishfield, Timmermans, et al., 2009) showed that in order for wind forcing to accumulate fresh water in the Beaufort Gyre, a fresh water anomaly must be available in the surface Ekman layer of the surrounding region. Freshwater sources are well known and include all waters with salinities less than the mean/reference salinity of the Arctic Ocean (here we use S=34.8). These are river influxes, Pacific water coming from Bering Strait, precipitation minus evaporation, and sea ice meltwater (e.g., Aagaard & Carmack, 1989; Carmack et al., 2008). Less understood is when and how fresh waters of different origin reach the Beaufort Gyre region and are accumulated there. Similarly, it remains an open question as to when and how the fresh water is released from the Beaufort Gyre region, and the subsequent pathways of fresh water exiting the Beaufort Gyre region.

To better understand the processes of fresh water accumulation and release, fresh water fluxes (both liquid and solid) were calculated along the boundaries of a rectangular region (RR) shown in Figures 2 and 7. A local maximum in freshwater content has been observed in this region every year since 2003. Moreover, the changes in sea ice and ocean parameters in this particular region are monitored by BGOS moorings, and the density of hydrographic stations and ITP data coverage in this region is higher than in the rest of the Beaufort Gyre region shown in Figures 2 and 7.

Assuming that Ekman transport is the major mechanism for accumulation of fresh water, freshwater fluxes are calculated along the region boundaries as horizontal components of Ekman volume transport in the upper 20 m normal to the boundaries multiplied by freshwater content in the upper 20 m of the water column at the boundaries. The Ekman transport is calculated as in section 2.5, and freshwater content at the boundaries is taken from the annual hydrographic surveys conducted between August and October. For other months of the year, freshwater content in the upper 20 m is reconstructed using changes in sea ice thickness measured by the ULSs at the mooring locations (Figure 3b) and ice concentration estimates from NSIDC data (section 2.5). In this calculation, fresh water is added to the ocean when sea ice melts and is removed from the upper 20‐m layer when sea ice grows. Changes of Ekman transport and freshwater content at the boundaries are shown in Figure S2). The seasonal cycle of freshwater content in the upper 20‐m layer was also reconstructed using ITP data (see also Figure 5h), which is in agreement with freshwater content changes due to sea ice transformations shown in Figure 5g.

**Figure 8:**
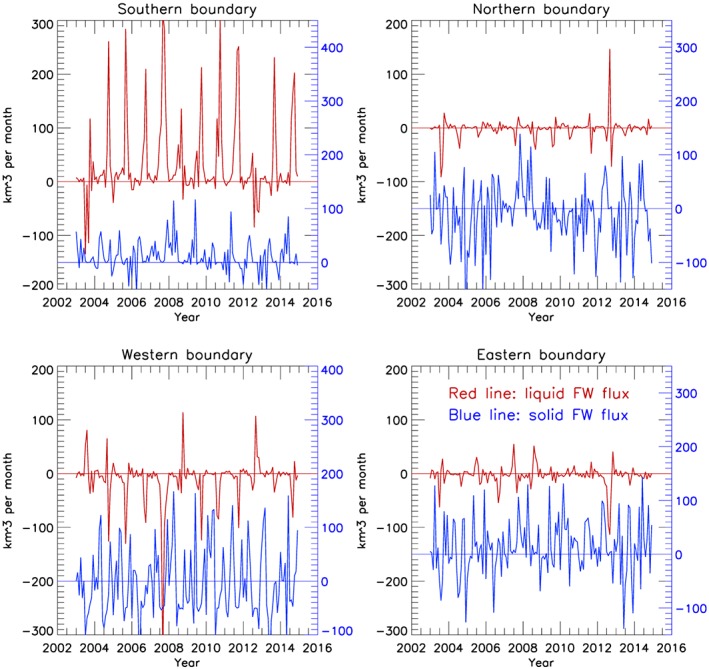
Monthly liquid freshwater fluxes (red lines; km^**3**^/month) calculated based on Ekman transport in the upper ocean 20‐m layer and freshwater content inferred at the boundaries of the rectangular region (Figure 7). Positive directions are northward and westward. The blue lines depict solid (ice) freshwater fluxes via boundaries.

In 2003–2018, the dominant freshwater flux to the region was via the southern boundary—up to 300 km^3^ of fresh water per month each summer (Figure 8). Also, a significant volume of fresh water was delivered to the region via the western boundary (negative flux means into the region). Freshwater fluxes via the northern and eastern boundaries were small except during 2012–2013, when freshwater flux was directed out of the region, resulting in the observed freshwater content reduction in these years (Figures 3 and 4). Rapid freshening of the region observed in 2007–2008 was associated with anomalous freshwater fluxes entering via the southern and western boundaries (Figure 8) driven by strong anticyclonic winds (Figure 7). The average contributions of Ekman transport and available fresh water (Figure S2) to the freshwater fluxes show that both contribute via the southern boundary to the increased freshwater content in the region. Despite the greatest amount of freshwater content being located along the region's eastern boundary, the Ekman transport along this line was very small.

Solid freshwater fluxes at the boundaries were calculated using monthly satellite‐based estimates of ice motion, concentration, and thickness (section 2.5). Results of sea ice draft measured at the four BGOS moorings are sufficiently representative to characterize changes of sea ice thickness in the Beaufort Gyre region (e.g., Krishfield et al., 2014; Proshutinsky, Krishfield, & Barber, 2009; Proshutinsky, Krishfield, Timmermans, et al., 2009; Yaremchuk et al., 2019, in this special issue). Sea ice plays a significant role in fresh water transport to the region via the southern, northern, and eastern boundaries, with fresh water transport out of the region via the western boundary (Figure 8).

**Figure 9:**
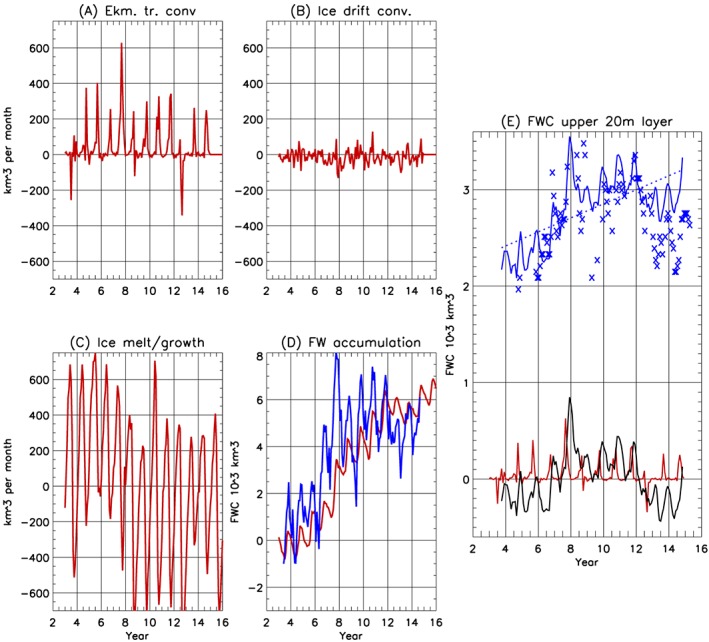
(a–d) Monthly components of fresh water contributions in the rectangular region (RR; Figures 2 and 7); (a) total freshwater content (FWC) changes due to liquid fresh water fluxes via boundaries of the RR; (b) solid FWC due to changes of ice volume associated with ice drift convergence in the RR; (c) FWC changes due to ice melt and growth in the RR; (d) freshwater accumulation due to dynamical and thermodynamic forcing (sum of FWC changes shown in panels a–c), where the blue line shows FWC inferred from satellite data for the RR. (e) FWC in the upper 20 m from annual hydrographic surveys corrected for seasonal sea ice transformations (blue line; see section 4.2) and from ITPs (crosses). The black line shows detrended FWC time series from seasonally corrected hydrographic data, and the red line depicts monthly fresh water volume entering the RR. The correlation of this time series and the FWC time series is 0.54 [0.345; 0.720] (maximum for a 2‐month lag, see text).

The results of the freshwater fluxes across the boundaries (i.e., the mechanical contribution to the regional freshwater budget) compared to freshwater content changes due to local sea ice melt/growth in the region (i.e., the thermodynamical contribution) indicate that fresh water accumulation in the region has been driven primarily by Ekman transport convergence (Figure 9), while sea ice transformations were mainly responsible for the significant seasonal changes. There is a good qualitative correlation between our calculations of freshwater accumulation shown in Figure 9d (red line) and freshwater content anomalies in the rectangular region inferred from satellite data (Figure 9d blue line). The observed seasonal changes are greater than from our calculations and can be explained by fresh water accumulation in the RR due to fluxes of fresh water to the region below the Ekman layer, which we have not taken into account.

Fresh water fluxes across the open boundaries of the rectangular region have been primarily responsible for the overall freshwater content increase in the region by about 5,500 km^3^ from 2003 to 2018. There is a clear negative trend in freshwater content in sea ice over the time series. From 2003 to 2018, melting sea ice was responsible for an increase of approximately 500 km^3^ of fresh water in the region (Figures 3 and 9). Note that Ekman transport convergence confined these melt waters in the Beaufort Gyre. Similar conclusions were recently published by Wang et al. (2019), who employed a global coupled ice‐ocean FESOM model with ~4.5‐km grid resolution in the Arctic Ocean to investigate the role of sea ice decline in the Arctic Ocean freshwater content change.

There is a 2‐month delay (Figure 9) between the maximum freshwater flux observed at the boundaries and the freshwater content maximum inferred from satellite data (correlation coefficient is 0.54 [0.345; 0.720]). This correlation with a 2‐month lag means that it takes about 2 months for the fresh water signal to propagate from the boundary to the center of the region (~300 km) with a speed of approximately 5.5 cm/s. This speed is a bit greater than the mean Ekman velocities in the upper 20‐m water layer shown in Figures 7 (panel B), but in these calculations we do not take into account freshwater content changes due to other factors like net precipitation and sea ice melt/growth. Uncertainties in forcing factors and sea ice conditions have to be taken into account as well.

### 4.2 Sources, Contributions, and Pathways of Fresh Water

Estimates of fresh water contributions from different sources in the Beaufort Gyre based on geochemical methods and analyses have been published by Yamamoto‐Kawai et al. (2005, 2008, 2009), Guay et al. (2009), Alkire et al. (2007, 2015), Alkire et al. (2017), Carmack et al. (2008, 2016), and Prowse et al. (2015). The most important conclusion from these studies is agreement that the Beaufort Gyre water column contains fresh water from all possible sources: meteoric, Eurasian and North American rivers, the Pacific Ocean, and sea ice melt. However, estimates of the fraction of fresh water contributed by different origins have differed substantially between these studies based on different data sets from different years.

BGOS assessments based on ULS data quantify the amount of sea ice as a source of fresh water. After 2006–2007 more sea ice melts than grows (Krishfield et al., 2014). The overall 500‐km^3^ local contribution of ice melt water from 2003 to 2018 is less than 8% of the total freshwater content change in the region. Thus, the vast majority of fresh water that accumulated in the Beaufort Gyre originated beyond the region.

Several numerical modeling studies have simulated fresh water content evolution using tracers to track the pathways of fresh water parcels released from rivers and sea ice, originating from precipitation, and transported with waters via ocean straits (e.g., Jahn et al., 2012; Pemberton et al., 2014) although none have analyzed the changes over the past 20 years. Two papers in this special issue (Hu & Myers, 2019; Kelly et al., 2019) describe results of model analyses of fresh water pathways from different sources to the Beaufort Gyre region. We next describe results from our analysis of modeling experiments to understand freshwater pathways.

Previous studies (Aksenov et al., 2016; Jahn et al., 2012; Maslowski et al., 2000, 2001; McLaughlin et al., 2002; Proshutinsky et al., 2001; Watanabe et al., 2017) have shown that trajectories of fresh water released from rivers and from Bering Strait (contributing as a fraction of Pacific water) change significantly in concert with the wind‐driven circulation. For example, during cyclonic regimes, Siberian river runoff is typically diverted toward the Laptev and East‐Siberian Seas (e.g., Maslowski et al., 2000, 2001), while during anticyclonic regimes, these waters do not extend toward the Canada basin. Similar flow path variability characterizes the Pacific waters originating from the Bering Strait region (i.e., Aksenov et al., 2016; McLaughlin et al., 2002) with the Pacific water outflow toward Canadian Archipelago Straits decreasing during years of anticyclonic circulation.

Analyses of chemical tracers collected during the BGOS hydrographic surveys (Guay et al., 2009; Yamamoto‐Kawai et al., 2005, 2008, 2009) and from North Pole Observatory aerial hydrographic surveys (Alkire et al., 2010); (Alkire et al., 2015) have allowed partitioning of meteoric water and net sea‐ice meltwater contributions, including estimation of their fractional contributions to the Beaufort Gyre region waters.

**Figure 10:**
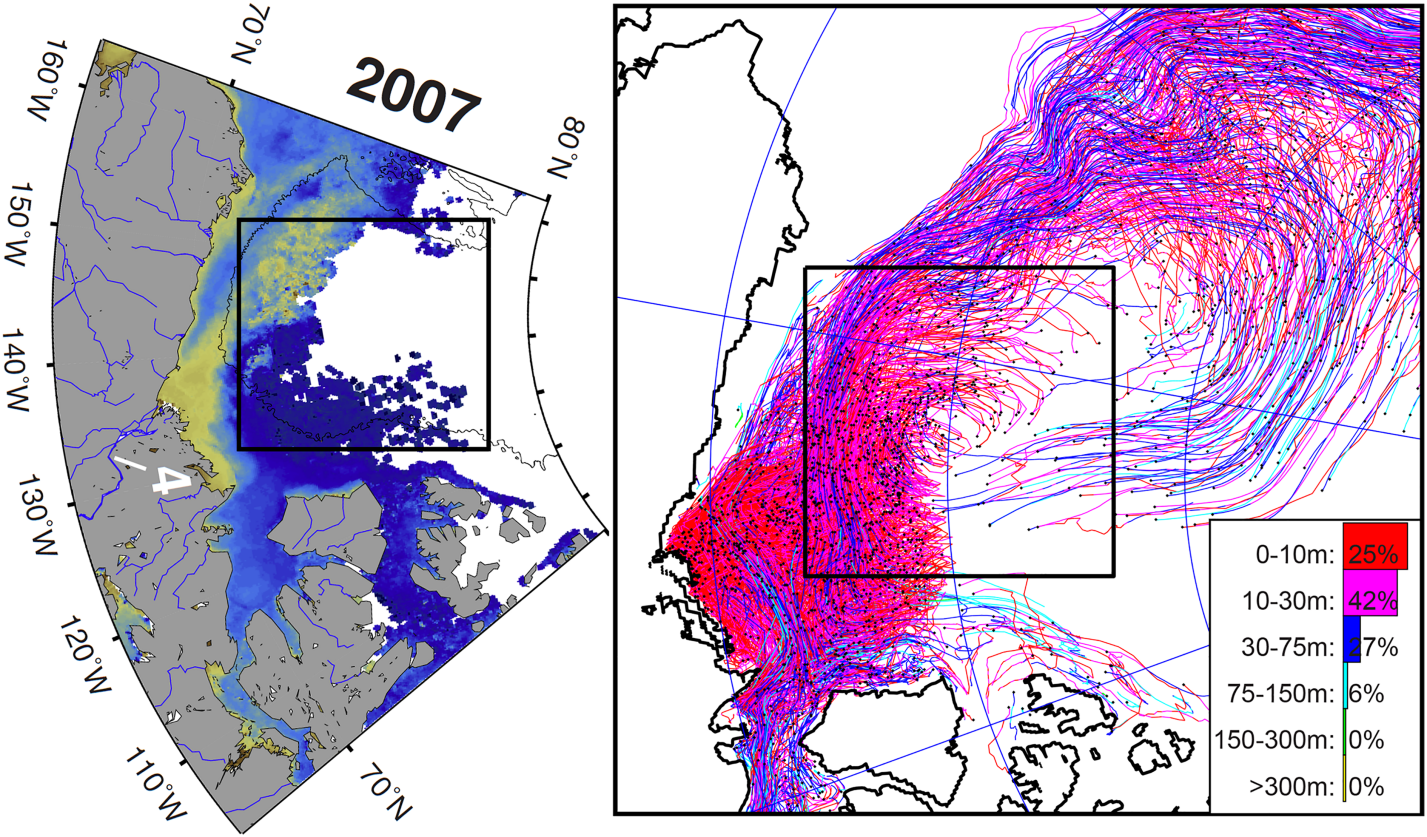
**(**left) The Mackenzie River origin water outflow observed in 2007 (from Fichot et al., 2013). Colors depict distribution of terrigenous dissolved organic matter (tDOM) as a surface manifestation of Mackenzie River water westward propagation (yellow is high concentration and blue is low). Moderate Resolution Imaging Spectroradiometer (MODIS) *Aqua* ocean color 4‐km resolution satellite data were used for analysis. The Mackenzie River is labeled (4). The contour line represents the 2,000‐m isobath and outlines the Canada Basin. (right) Distribution of floats released since 2000 at the Mackenzie River mouth and their trajectories at the end of 2007. Colors of trajectories and legend show percentage of floats at different depths. Results are from the SibCIOM model (see section 2.6 and supporting information S1). The black bounded region is the same as shown in Figures 2 and 7.

**Figure 11:**
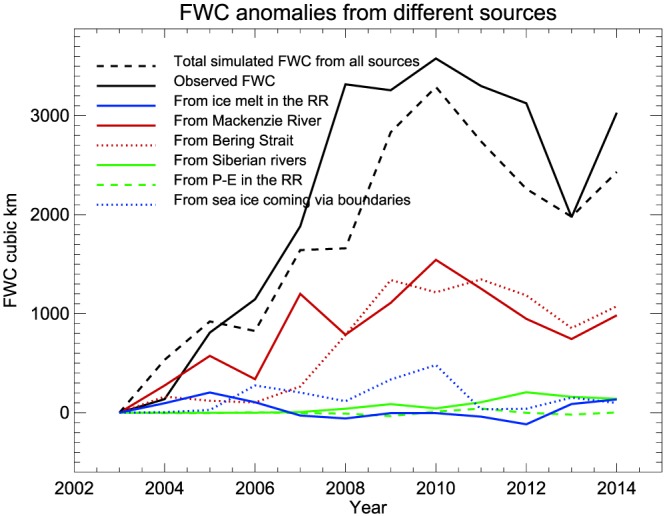
Annual freshwater content anomalies from observations and model results (km^**3**^) for the rectangular region (RR). Freshwater content from sea ice, rivers, and Bering Strait is from tracer analysis. Net precipitation (P‐E) is from NCAR/NCEP reanalysis. Freshwater content contribution from ice melt in the RR and freshwater flux via boundaries are from the calculations described in section 4.2.

**Figure 12:**
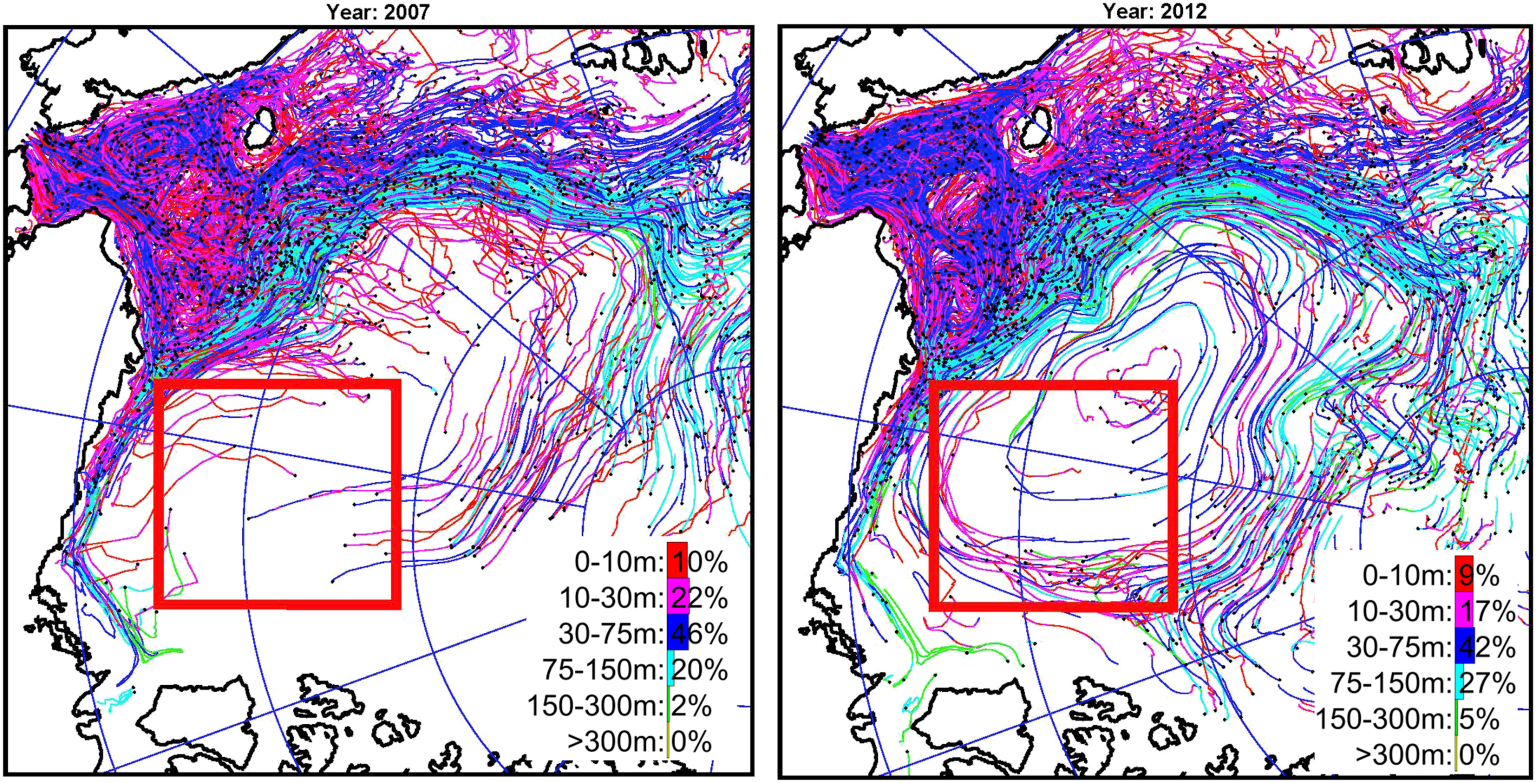
Trajectories of floats released from Bering Strait since 2000 (left) by the end of 2007 and (right) by the end of 2012 based on SibCIOM model results. The Colored bars show the percentage of floats in different layers. The bounded region is the same as shown in Figure 7.

However, there are some differences among these analyses related to difficulties in separating fresh water from sea ice melt and meteoric water and distinguishing between North American and Siberian River runoff. To address these difficulties, and in recognition that there are significant gaps in the data, numerical modeling was employed to better understand the redistribution of fresh water originating from different rivers and straits. Three numerical experiments were conducted employing the SibCIOM model. Trajectories beginning in the year 2000 were initiated at the mouth of the Mackenzie River (experiment 1) and in Bering Strait (experiment 2). In experiment 3, the floats were released in 1985 in the deltas of the Siberian Rivers (mouths of the Kolyma, Indigirka, Yana, Lena, Olenek, Ob, and Yenisei rivers). The algorithm for the float trajectory simulations includes vertical and horizontal diffusion processes given by specified mixing coefficients. Results corresponding to float releases at each of these locations are described below (see also Figures 10, 11, 12).

**Figure 13:**
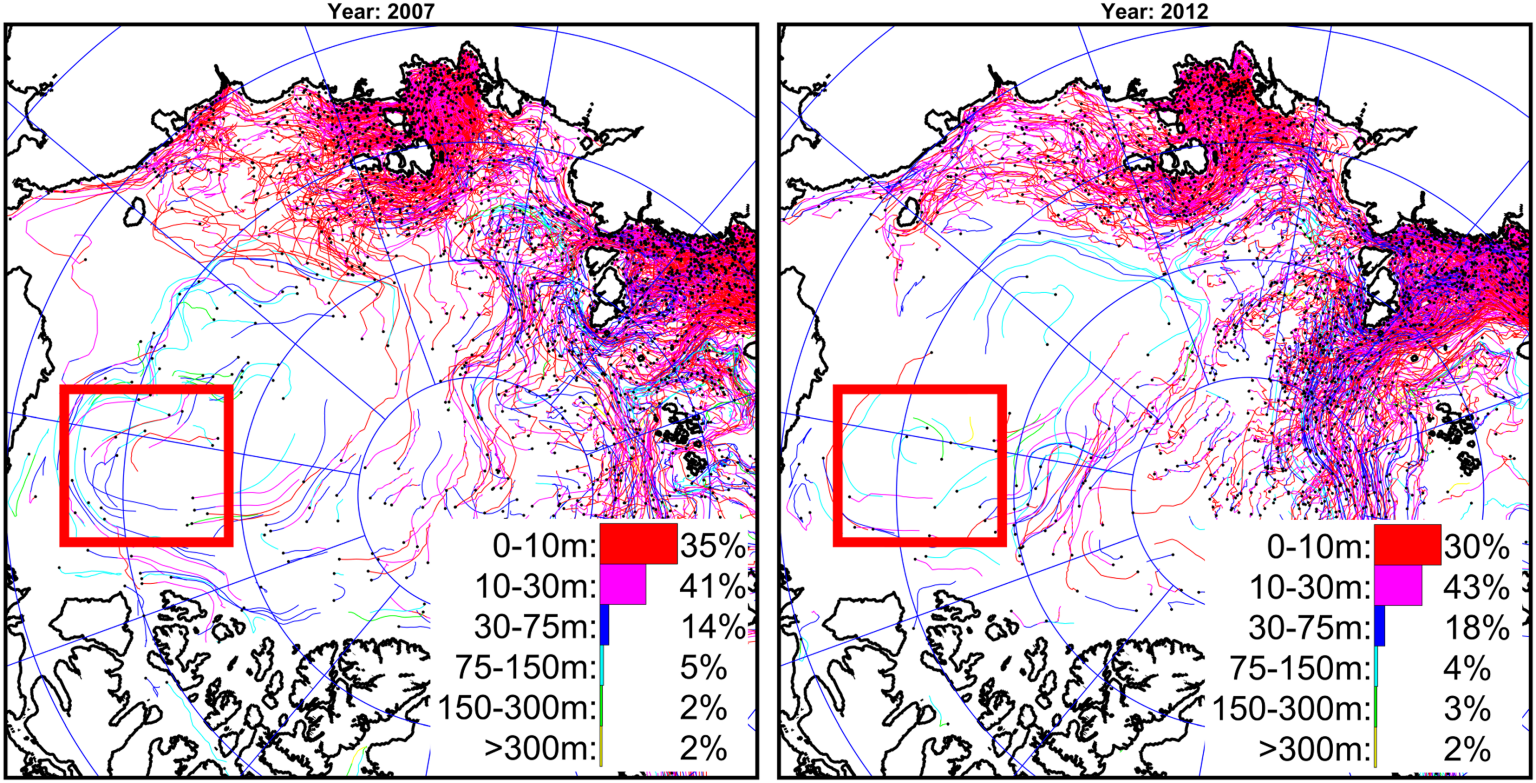
Same as in Figure 12 but for floats released from Siberian rivers since 1 January 1985 by the end of 2007 and 2012. The colored bars show the percentage of floats in different water column layers. The bounded region is the same as shown in Figure 7.

#### 4.2.1 Mackenzie River

The Mackenzie River annual runoff is ~300 km^3^/a (Holmes et al., 2019). Fichot et al. (2013) analyzed MODIS Aqua ocean color 4‐km resolution satellite data and found that a transition from eastward to northwestward advection of Mackenzie River runoff occurred between 2002 and 2011 (Figure 10). The Mackenzie River release simulation is in agreement with those observations (Figure 10). It took slightly more than 1 year for floats released at the Mackenzie River mouth (not shown) to reach the southern boundaries of our rectangular region and to influence freshwater content in this area. The right panel of Figure 10 clearly demonstrates that substantial volumes of fresh water released from the Mackenzie River propagate through the Beaufort Gyre region and remain there or return to the region via the western and northern boundaries under the influence of the wind‐driven anticyclonic circulation during the period of our study. This is also in agreement with Figure 8 where fresh water accumulation in the region is shown to be due to Ekman transport convergence with the major influx of fresh water along the southern boundary (Figure 8). The numerical simulations indicate (Figure 11, red line) that over the period 2003–2012, water from the Mackenzie River was one of the major sources of fresh water that accumulated in the region. Three peaks (red solid line, Figure 11) were identified: in 2005, 2007, and 2010; these are in agreement with the observed freshwater content changes in the region (black solid line in Figure 11). Supporting information S2 with Figures S3a–S3h show observed water salinity at different depths confirming that freshwater flux via southern boundary originated from the Mackenzie River delta.

#### 4.2.2 Bering Strait

The observed increase of Beaufort Gyre region freshwater content may be associated with fresh water originating at Bering Strait. Haine et al. (2015) estimated that the annual flux of fresh water to the Arctic Ocean from Bering Strait is 2,500±100 km^3^/a. This flux could “fill” the Beaufort Gyre region with the observed freshwater anomaly of 6,400 km^3^ in 2.6 years; see, for example, the analysis of Timmermans et al. (2014) demonstrating how Pacific Water is swept into the Beaufort Gyre. Numerical modeling experiments were conducted to assess how much of the Pacific fresh water can be accumulated in the Beaufort Gyre under the influence of the 2003–2018 anticyclonic winds? The simulations (Figures 11 and 12) reveal that in 2003–2007, the freshwater flux anomaly to the rectangular region from Bering Strait was low due to very strong anticyclonic winds (Figure 7). Note that trajectories of floats shown in Figure 12 are in excellent agreement with SLPs, sea ice drift, and geostrophic currents (Figures 7). In 2008 and later, the floats from the Bering Strait region were able to reach the region mainly via the northern boundary and in some years via the southern and western boundaries. By 2012, the contribution of fresh water to the region from Bering Strait is very close or even greater than that from the Mackenzie River (Figure 11).

#### 4.2.3 Siberian Rivers

**Figure 14:**
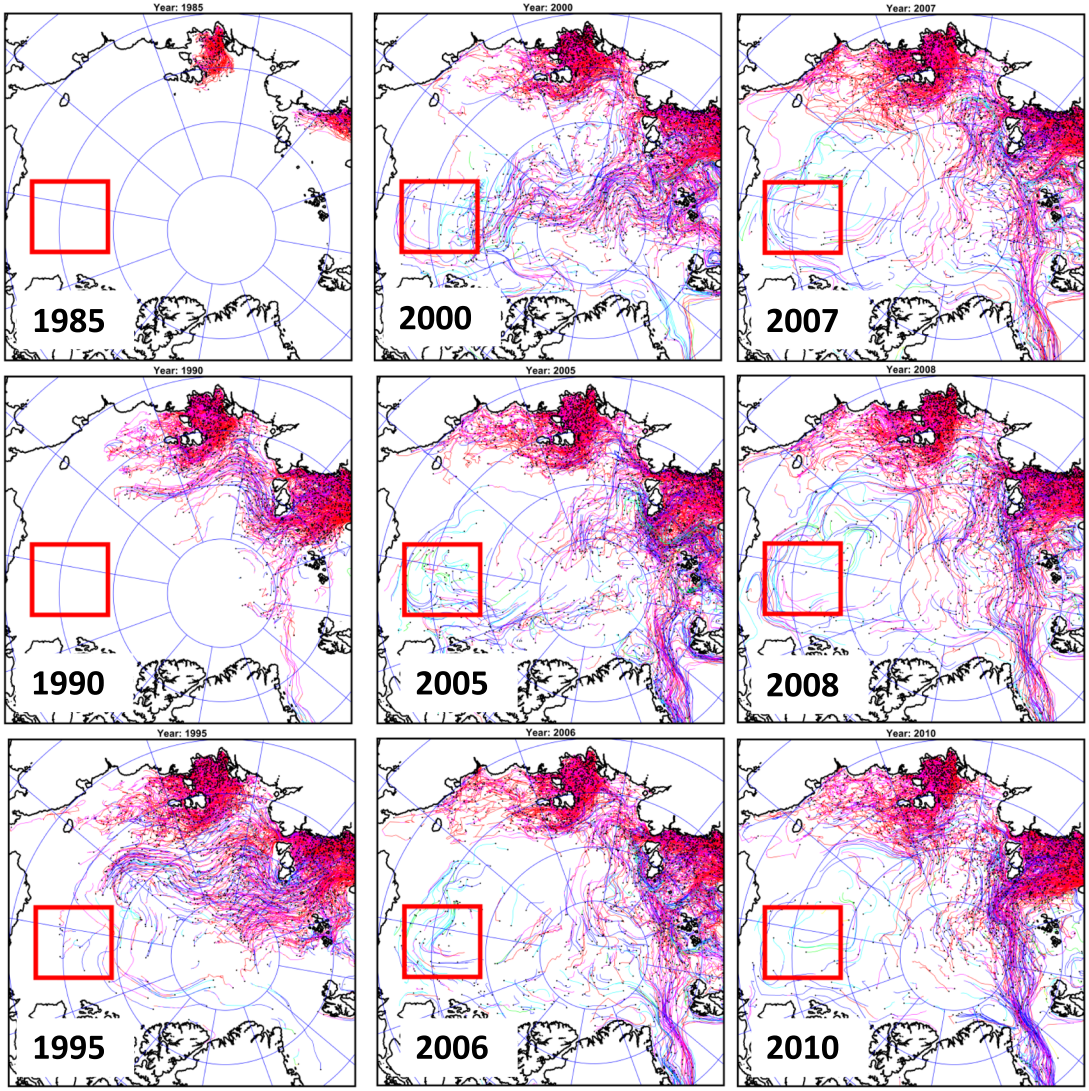
Trajectories of floats released from Siberian rivers since 1 January 1985 by the end of 1985,1990,1995,2000, 2005, 2006, 2007, 2008, and 2010 based on SibCIOM model results. During cyclonic circulation regime of 1989–1996 (see Proshutinsky et al., 2015), water floats from Siberian rivers reached the rectangular region (bounded by red line); by 2005 their number reached maximum, and then in 2006–2010 these particles were leaving the RR. RR is the same as shown in Figures 2 and 7. These distributions of floats illustrate that freshwater content anomaly in 2007 relative to 2000 was close to 0.

The total freshwater flux from Siberian rivers is close to 2,000 km^3^/a (Holmes et al., 2015). Our modeling results suggest that the Siberian river contributions to fresh water accumulation in the Beaufort Gyre region in 2003–2018 are negligible relative to the Bering Strait and Mackenzie River contributions (Figures 12 and 11). Note that the results of these simulations do not contradict the results of the geochemical analysis by Morison et al. (2012), who found fresh water originating from Siberian rivers in the Beaufort Gyre region (see also Alkire et al., 2015). Instead, it is plausible that the detected waters are remnants of Siberian river waters that reached the Beaufort Gyre region during the previous cyclonic circulation regime (1989–1996; Figure 14). After 1996, these waters may have been trapped in the Beaufort Gyre region by sustained anticyclonic winds with some additions from surrounding regions due to anticyclonic circulation. After 2005, more Siberian origin floats were removed from the region than added, and by 2007, the freshwater content anomaly due to Siberian rivers relative to 2003 was close to zero and then became negative. Based on these modeling results, we conclude that the contribution from Siberian rivers to the increase in Beaufort Gyre region fresh water content between 2003 and 2014 is very small. Those Siberian river waters that arrived previously were trapped in the region, and their “concentration” has not changed or waned after 2007. The confinement of fresh water in the Beaufort Gyre by anticyclonic winds in 2005–2010 was also shown in Timmermans et al. (2011).

Consequently, we conclude that fresh water accumulation in the Beaufort Gyre region was the product of persistent anticyclonic atmospheric wind forcing (1997–2018; see Proshutinsky et al., 2015) accompanied by sea ice melt. The former induced an anomalous wind‐forced redirection of Mackenzie River discharge from predominantly eastward to westward flow supplying the Beaufort Gyre region with its fresh water. There was in addition a contribution of waters of Pacific Ocean origin via Bering Strait. The fresh water input to the Beaufort Gyre region from Siberian rivers during 2003–2018 was negligible. Remnants of Siberian river waters detected by geochemical analysis presumably penetrated into the region in 1989–1996 during a cyclonic circulation regime.

**Figure 15:**
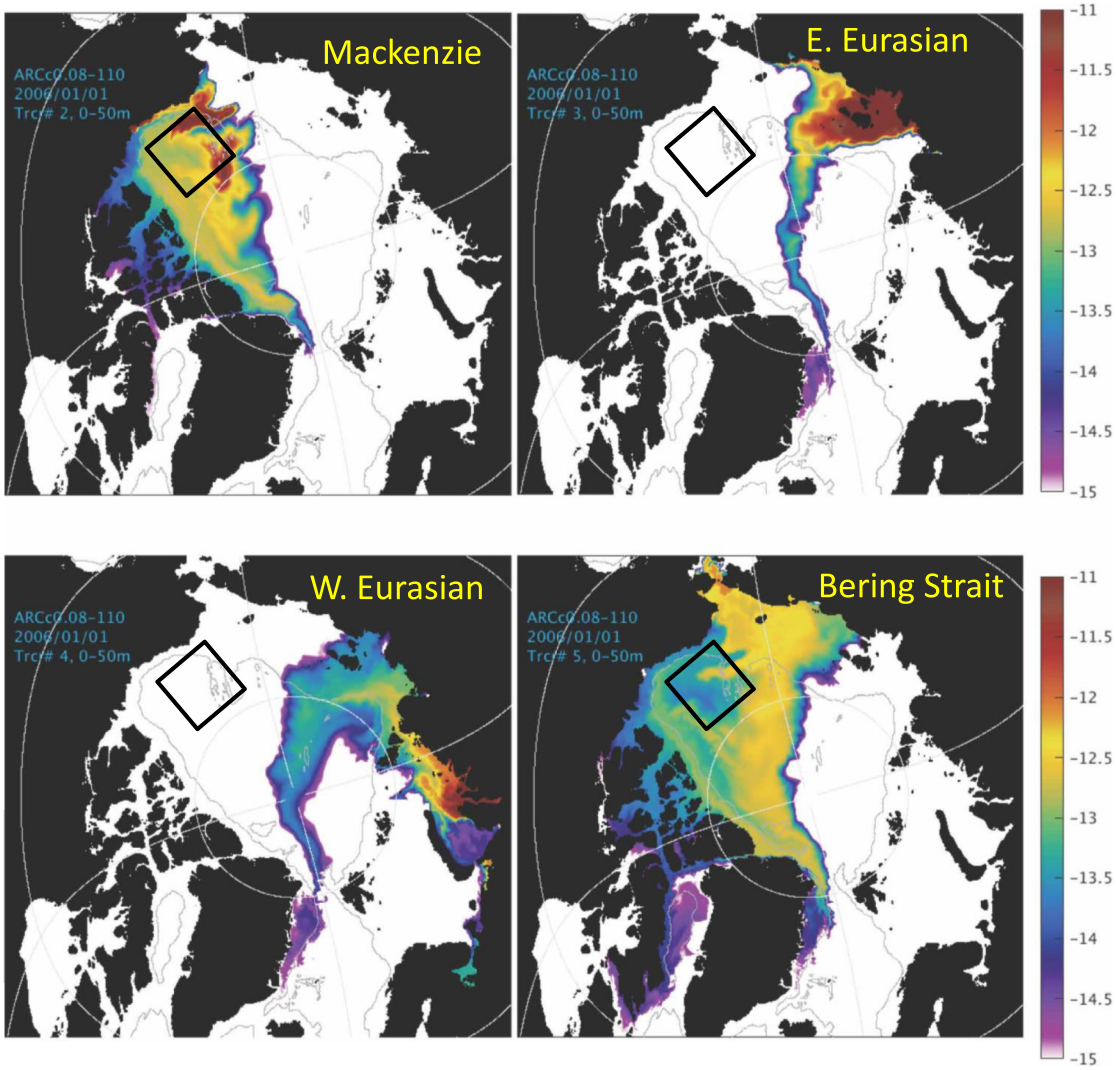
January 2006 fraction of a passive tracer (log10 scale) that was released since 1993 from different sources (noted in the panel titles) from HYCOM 1/12° model results. The Beaufort Gyre region is depicted by the black box (same as in Figure 7).

Additional validation of these findings was conducted through comparison with results of tracer experiments employing a 0.08° (~4.5‐km horizontal resolution) HYbrid Coordinate Ocean Model (HYCOM) model. The model details and passive tracer calculations are provided in Dukhovskoy et al. (2019; this special issue). Since 1993, site‐specific passive tracers were released continuously at the mouths of the Mackenzie River, East Eurasian Rivers (Kolyma, Lena, and Khatanga), West Eurasian Rivers (Pyasina, Enisey, Ob, Pechora, and Sev. Dvina), and in Bering Strait. Tracer concentrations were proportional to the monthly freshwater fluxes from each source. The distribution of tracers from these sources (Figure 15) is in agreement with our conclusions. Our results are also supported by simulations of particles released in the Beaufort Gyre (Kelly et al., 2019; this special issue) and using a backward trajectories method to track particle origins based on results of the 1/12° NEMO model. It was found that pathways of fresh water from Mackenzie and Bering Strait depend significantly on the sense and intensity of the atmospheric circulation regime and that since 2000, the contribution of fresh water from the Mackenzie River to the accumulation in the Beaufort Gyre region was comparable with the freshwater volume transported to the region from Bering Strait. The role of Siberian rivers decreased substantially after 1990s.

## 5 Summary

Here we summarize the key conclusions of this paper. Some conclusions are still unsettled and motivate future investigation.

### 5.1 Beaufort Gyre Freshwater Content Time Series (2003–2018) From Different Data Sets Are Updated, Compared, and Analyzed

The data from BGOS moorings and ITPs were jointly analyzed with freshwater content estimates from annual hydrographic surveys, and data inferred from remote sensing. This study has demonstrated the relative strengths and weakness of the different datasets for measuring Beaufort Gyre freshwater content:

1. The most accurate measure of local freshwater content is the August/September/October BGOS CTD casts, but while they capture interannual variability well over the long‐term, they are only a monthly snapshot and do not resolve month‐to‐month or seasonal variability, and are limited in their spatial coverage.
2. The BGEP moorings, on the other hand, offer year‐round measurements over a long period, but they are only available at three or four locations and only capture freshwater content variability below 65‐m depth.
3. ITPs offer 6‐ to 750‐m measurements almost completely through the fresh water layer but have irregular spatial and temporal sampling.
4. Freshwater content derived from satellite SSH estimates offers the best spatial and temporal coverage but rely on relatively uncertain empirical relationships between SSH and freshwater content, and they only represent an integrated measure of freshwater content, with no information about its vertical distribution in the water column.

Despite significant uncertainties in the different data sets, this study has demonstrated the synergistic value of having multiple diverse observations to obtain a comprehensive understanding of Beaufort Gyre freshwater content variability. BGOS surveys clearly show the interannual increase in freshwater content, but without satellite or ITP measurements it would not be possible to resolve the seasonal cycle of freshwater content, which is larger than the year‐to‐year variability.

### 5.2 Qualitative and Quantitative Estimates of Factors and Mechanisms Driving Freshwater Content Changes Are Provided

This paper focuses on two major factors influencing rates of freshwater accumulation and release in the Beaufort Gyre region, namely, wind‐driven Ekman transport convergence and the availability of fresh water for accumulation. Ekman transport convergence and freshwater content changes due to sea ice seasonal transformations from growth to melt are analyzed. While these major factors and processes have been known and discussed in many publications starting from Proshutinsky et al. (2002) and the many subsequent works by others cited above, in this paper the role of these factors is evaluated not only qualitatively but quantitatively and illustrated by observational evidence from different data sources. The major result of this analysis is that the seasonal changes of Beaufort Gyre region freshwater content are mainly dominated by sea ice melting and growth, while net annual fresh water accumulation and release are driven by changes of Ekman transport modified by sea ice conditions and the sense and intensity of the ocean geostrophic circulation. Based on annual hydrographic surveys, the Beaufort Gyre region accumulated approximately 6,400 km^3^ of fresh water (relative to 34.8 reference salinity) over a 16‐year period. This represents a 40% increase of fresh water volume in the region relative to the climatology of the 1970s. While the average rate of fresh water accumulation was 397±116 km^3^/a, the freshwater content growth was not uniform due to interannual changes in the wind, sea ice conditions, and ocean geostrophic currents. The ocean circulation played a flywheel role regulating momentum transfer from the wind into the ocean (regulated by sea ice conditions), effectively damping disturbances in external forcing and stabilizing freshwater content changes.

In 2003–2008, freshwater content measured in August–September increased by 4,900±1.550 km^3^, from 16,900±1,400 km^3^ in 2003 to 21,800±1,700km^3^ in 2008, mainly due to anomalous Ekman pumping under strong anticyclonic winds and reduced sea ice cover. Freshwater content stabilized around 22,000±1,900 km^3^ for a 4‐year period under consistent wind forcing and more or less stable sea ice conditions. In 2013, freshwater content decreased by about 1,600±500 km^3^ to almost the volume observed in 2007. After 2013, the changes of freshwater content were mostly regulated by interplay among strength of Ekman transport convergence, sea ice conditions, and intensity of geostrophic ocean circulation. In 2014–2016, freshwater content again increased to reach a record maximum of around 23,200±1,800 km^3^ in 2016 due to intensification of anticyclonic winds and some reduction of sea ice cover. In 2017 and 2018, the freshwater content changed only modestly to 23,400±1,800km^3^ and 23,300±2,000 km^3^, respectively, due to small changes in prevailing winds, ocean currents, and sea ice cover. While we have not analyzed processes and mechanisms of freshwater accumulation and release in the region due to eddies and mixing, recent studies suggest that eddy fluxes can balance wind‐driven momentum flux into the Beaufort Gyre and limit fresh water accumulation (e.g., Manucharyan & Spall, 2016; Meneghello et al., 2017).

### 5.3 In 2003–2018, the Major Sources of Accumulated Freshwater Were Sea Ice Melt, Mackenzie River Runoff, and Bering Strait Transport

Observations show that local cumulative melting of sea ice (thermodynamic effect) and the convergence of melt water from surrounding regions (dynamical effect) contributed some 10% to 20% of fresh water to the central Beaufort Gyre region over the study period. Our numerical simulations indicate that the fresh water from the Mackenzie River was one of the major sources (from 15 to 45% depending on year) responsible for freshwater content increase in the region with peaks observed and simulated in 2005, 2007, and 2010. The fresh water originated from the Bering Strait region contributed from 5 to 50% depending on year. Over the 2003 to 2014 period, fresh water contributed by Siberian rivers ranged between 0 and 6% of the observed Beaufort Gyre freshwater content anomaly.

## Supporting information



Supporting Information S1
