# Supplementary material for: Analysis of the Beaufort Gyre Freshwater Content in 2003–2018
Source: J Geophys Res Oceans. 2019 Dec 28;124(12):9658–89. doi: 10.1029/2019JC015281 (PMC7003849; doi:10.1029/2019JC015281)
Supplement: Supplementary file 1 — Supporting Information S1 [file JGRC-124-9658-s001.docx]

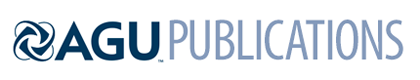


*Journal Geophysical Research - Oceans*

Supporting Information for

Analysis of the Beaufort Gyre freshwater content in 2003-2018

**A. Proshutinsky^1^, R. Krishfield^1^, J. Toole^1^, M.-L. Timmermans^2^, W. Williams^3^, S. Zimmerman^3^, M. Yamamoto-Kawai^4^, T. W. K. Armitage^5^, D. Dukhovskoy^6^, E. Golubeva^7,8^, G. E. Manucharyan^5^, G. Platov^7,8^, E. Watanabe^9^, T. Kikuchi^9^, S. Nishino^9^, M. Itoh^9^, S.-H. Kang^10^, K.-H. Cho^10^, K. Tateyama^11^ and J. Zhao^12^**

^1^Woods Hole Oceanographic Institution, MA, USA

^2^Yale University, CT, USA

^3^Fisheries and Oceans Canada, Institute of Ocean Sciences, Sidney, British Columbia, Canada

^4^Tokyo University of Marine Science and Technology, Tokyo, Japan

^5^Jet Propulsion Laboratory, California Institute of Technology, Pasadena, CA, USA

^6^Florida State University, FL, USA

^7^Institute of Computational Mathematics and Mathematical Geophysics, Siberian Branch of Russian Academy of Science, Novosibirsk, Russia

^8^Novosibirsk State University, Novosibirsk, Russia

^9^Japan Agency for Marine-Earth Science and Technology (JAMSTEC), Yokosuka, Kanagawa, Japan

^10^Korea Polar Research Institute (KOPRI), Incheon, Republic of Korea

^11^Kitami Institute of Technology, Hokkaido, Japan

^12^Ocean University China, China

**Contents of this file**

1. **Introduction**
   1. Text S1: Particle tracer in SibCIOM model
2. **Figures**
   1. Figures S1a to S1g: panels showing processes of fresh water accumulation in the BG region
   2. Figure S2: Monthly components of Ekman velocities at boundaries
   3. Figures S3a-3h: Regional salinity distributions at 1m, 5m, 10m and 20m from ammual hydrographic surveys
   4. Figures S4a, S4b: Observed and simulated river runoff from Mackenzie River in 2002-2010
3. **Annual and monthly data for Figures 2 and 3**
   1. S5.1 Figure 2: Annual FWC from moorings
   2. S5.2 Figure 3, top panel: FWC from hydrography
   3. S5.3 Figure 3, top panel: FWC from ITPs
   4. S5.4 Figure 3, top panel: FWC inferred from SSH
   5. S5.5 Figure 3, top panel: FWC from sea ice
   6. S5.6 Figure 3, top panel: FWC from model results
   7. S5.7 Figure 3, top panel: FWC at mooring location inferred from SSH
   8. S5.8 Figure 3, top panel: FWC from moorings below 65m
   9. S5.9 Figure 3, bottom panel: all parameters
4. **Introduction**

ICMMG (Institute of Computational Mathematics and Mathematical Geophysics) model description is provided below. In addition, model’s particle transport algorithm is discussed to clarify results of tracer trajectories discussed in the paper.

Attached figures illustrate forcing and results of Ekman velocities calculations in the Beaufort Gyre of the Arctic Ocean and freshwater fluxes via boundaries.

## 1.1 Particle tracer in SibCIOM model

In order to numerically track the distribution of water masses in SibCIOM model (see section 2.6 of this paper), a so-called distributed tracer is used. This tracer is similar to a dye and is a three-dimensional (or two-dimensional) concentration field that has some specific source and evolves in time like salinity or temperature (Maslowski et al. 2000, Condron et al. 2009, Jahn et al. 2010, Aksenov et al., 2016, Dukhovskoy et al., 2016). The drawback of this approach is the lack of information about individual trajectories. One can state what is the tracer concentration in a certain cell of a numerical grid, but information about how this tracer got there, when and where it was emitted, what its initial temperature and salinity were, what its further movement is, cannot be established via this approach. An alternative approach is the method of Lagrangian particles. Particles are emitted individually and periodically in the region of a certain source and move within the numerical domain with a model velocity. In this case, all the above information is available The tracer concentration is estimated based on how many tracer particles are currently inside a given cell.

To calculate the position of a particle $\vec{\boldsymbol{r}}$ moving with velocity $\vec{\boldsymbol{v}}$ from the initial point $\vec{\boldsymbol{r}_{\boldsymbol{0}}}$, one can use the explicit advection equation in the form of Lagrange

$$\vec{\boldsymbol{r}}=\vec{\boldsymbol{r}_{\boldsymbol{0}}}+\vec{\boldsymbol{v}}\boldsymbol{\cdot}dt,$$

where velocity $\vec{\boldsymbol{v}}$ is an interpolant of the model velocity field at a point $\vec{\boldsymbol{r}_{\boldsymbol{0}}}$, and $dt$ is a model time step. The explicit form is easy to use, but a problem arises when the speed is so great that, if it is saved during the whole time step, the particle could leave the domain. In this case a more acceptable solution is obtained under the assumption that the velocity decreases linearly to zero as the boundary is approached. If the boundary of the region has the coordinate $x=0$, and the particle starts its motion from the point with the coordinate $x_{0}>0$ with the negative initial velocity $v_{0}=-\left| v_{0} \right|<0$, then the explicit expression gives a new position with the coordinate $x=x_{0}-\left| v_{0} \right|dt$, which can be negative, that is, the particle crosses the boundary. If the particle velocity decreases linearly to zero when approaching the boundary, i.e. $v\left( x \right)=-\left| v_{0} \right|\frac{x}{x_{0}}$, the Lagrange formula yields the following differential equation

$$\frac{dx}{dt}=-\left( \frac{\left| v_{0} \right|}{x_{0}} \right)x,$$

which has an exponential solution. For one model time step period it gives

$$x\left( t \right)=x_{0}\exp\left( -\frac{\left| v_{0} \right|}{x_{0}}dt \right).$$

That is, a particle exponentially approaches the boundary, but does not intersect it.

The advective motion of particles is additionally accompanied by diffusion. Diffusion is considered here as a stochastic process. As a result of diffusion, the position of a particle will be randomized (relative to its position obtained after advection) with a normal distribution and with a standard deviation proportional to the magnitude of the diffusion scale, $\sqrt{A\cdot dt}$, where $A$ is the diffusion coefficient. If the position of the particle after advection is determined by the coordinate $x_{a}$, and the standard deviation along the $X$ coordinate is $\sigma_{x}=\alpha\sqrt{A\cdot dt}$ ($\alpha$ is a scaling factor), then particle position $x_{d}$ after diffusion will be

$$x_{d}=x_{a}+\sigma_{x}\cdot\Phi^{-1}\left( \mathrm{rand}-\frac{1}{2} \right),$$

where $\mathrm{rand}$ denotes a random number of a uniform distribution on the interval (0,1) numerically realized on a computer, and $\Phi^{-1}$ is the inverse function to the Laplace function, i.e.

$$x=\Phi^{-1}\left( y \right): y=\Phi\left( x \right)=\frac{1}{\sqrt{2\pi}}\int_{0}^{x} e^{-\frac{\xi^{2}}{2}}d\xi.$$

A similar procedure is performed using the other two model coordinates.

The position of a particle tracing water subject to convective or wind-driven mixing is also determined stochastically on the basis of a uniform distribution in the mixed layer

$$z=z_{l}+\left( z_{u}-z_{l} \right)\cdot rand,$$

where $z_{u}$ and $z_{l}$ are the upper and the lower boundaries of the mixed layer.

If particles are generated with a certain periodicity, then over time their number becomes very large, which significantly slows the model calculations. Furthermore, over time, particles lose their identity as indicators of a particular source of water mass due to diffusion processes. Guided by these two reasons, we have also introduced a particle elimination process that limits the number of older particles. Removal of particles also occurs randomly in accordance with the exponential distribution of their lifetime. If we assume that the average lifetime of a particle is $\tau\gg dt$, then, in accordance with the exponential distribution law, the probability of particle removal during a model time step is

$$P_{d}=1-\exp\left( -\frac{dt}{\tau} \right)\approx\frac{dt}{\tau}.$$

The probability that the particle will remain active for a certain period of time $t$ is

$$P_{a}\left( t \right)=\exp\left( -\frac{t}{\tau} \right).$$

If we assume that the decrease in the number of particles should not lead to a decrease in the water masses represented by them, the elimination of some particles of age $t$ should increase the weight of the remaining particles of this age. This means that the weight of the surviving particles increases with time by a factor of

$$C=\frac{1}{P_{a}\left( t \right)}=\exp\left( \frac{t}{\tau} \right).$$

If each particle initially represents a volume $V_{0}$ of some water mass, and some area contains $N$ particles of different ages $t_{i}$, then the associated water mass volume in this area will be

$$V=\sum_{i=1}^{N} V_{0}\exp\left( \frac{t_{i}}{\tau} \right).$$

In our numerical experiment we used $\tau=5$ years with model time step $dt=1.5$ hours. A particle of any river runoff was deployed in a way that it represents a volume $V_{0}=0.6859$ km^3^, while each particle of Bering Strait inflow represents $V_{0}=38.81\frac{34.8-S_{0}}{34.8}$ km^3^. where $S_{0}$ is the salinity in place of origin and at time of origin.

1. Figures
   1. **Supplemental S1**


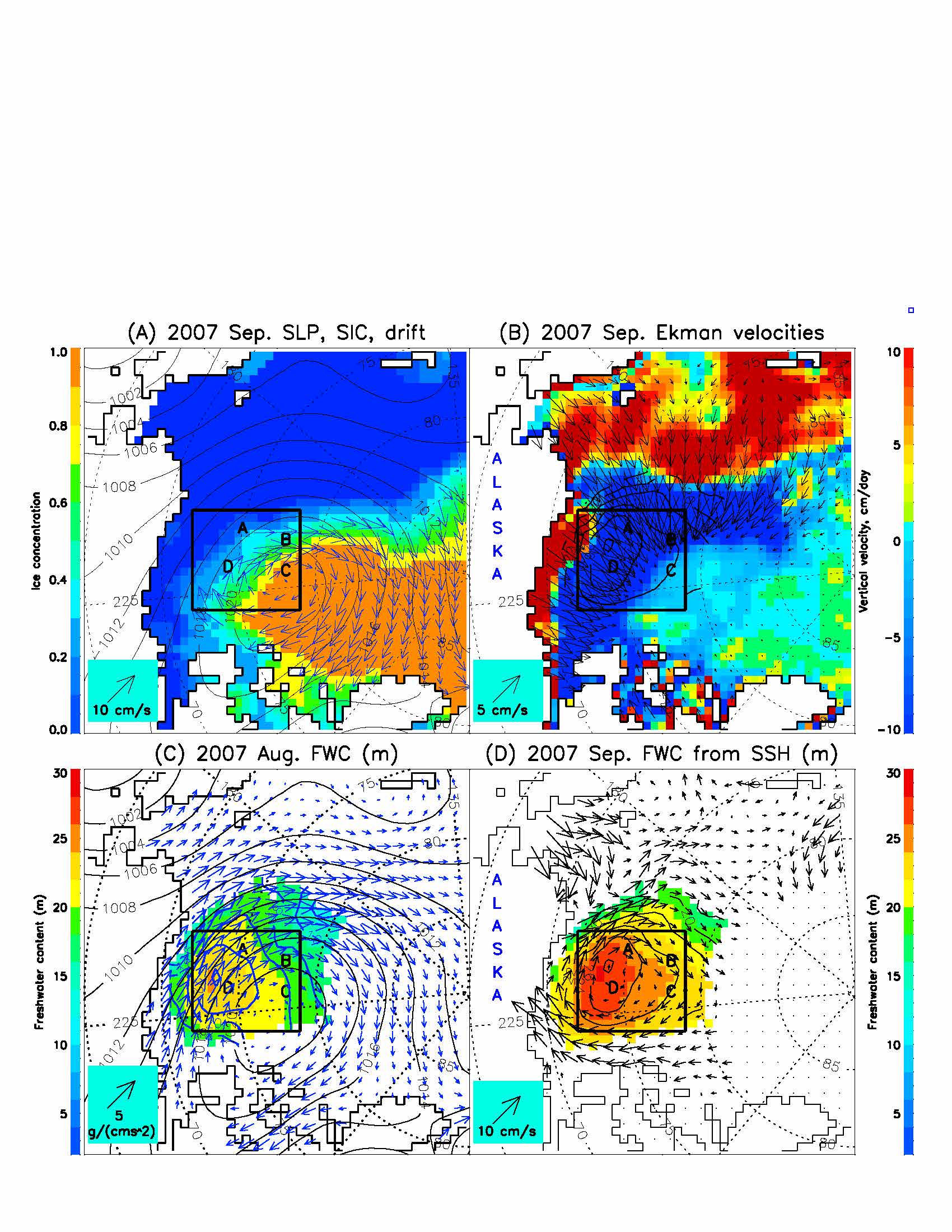


Figure S1a Monthly freshwater content and forcing factors in the Beaufort Gyre region in September 2007. (A): sea ice concentration (colors), ice drift (vectors, cms^-1^). Black contours show SLP (hPa). (B): Ekman velocities. Horizontal Ekman mean velocities are depicted by vectors (cms^-1^) and Ekman vertical velocity (cmday^-1^) by colors. Black solid lines represent freshwater content (m) with 2 meter increment. (C): freshwater content from CTD data is shown in colors and by blue contours (m, 2 m increment); Vectors show wind stresses at the ice and ocean surface (gcm^-1^s^-2^); (D): August freshwater content (m, colors and contours) inferred from DOT. Vectors depict geostrophic currents inferred from DOT and published by Armitage et al. (2017). Note: we intentionally repeat freshwater content observed in the August hydrographic survey (panels C in all Figures 6) to better visualize the rate of fresh water accumulation and accurately compare observed freshwater content in August with freshwater content inferred from the monthly satellite data.


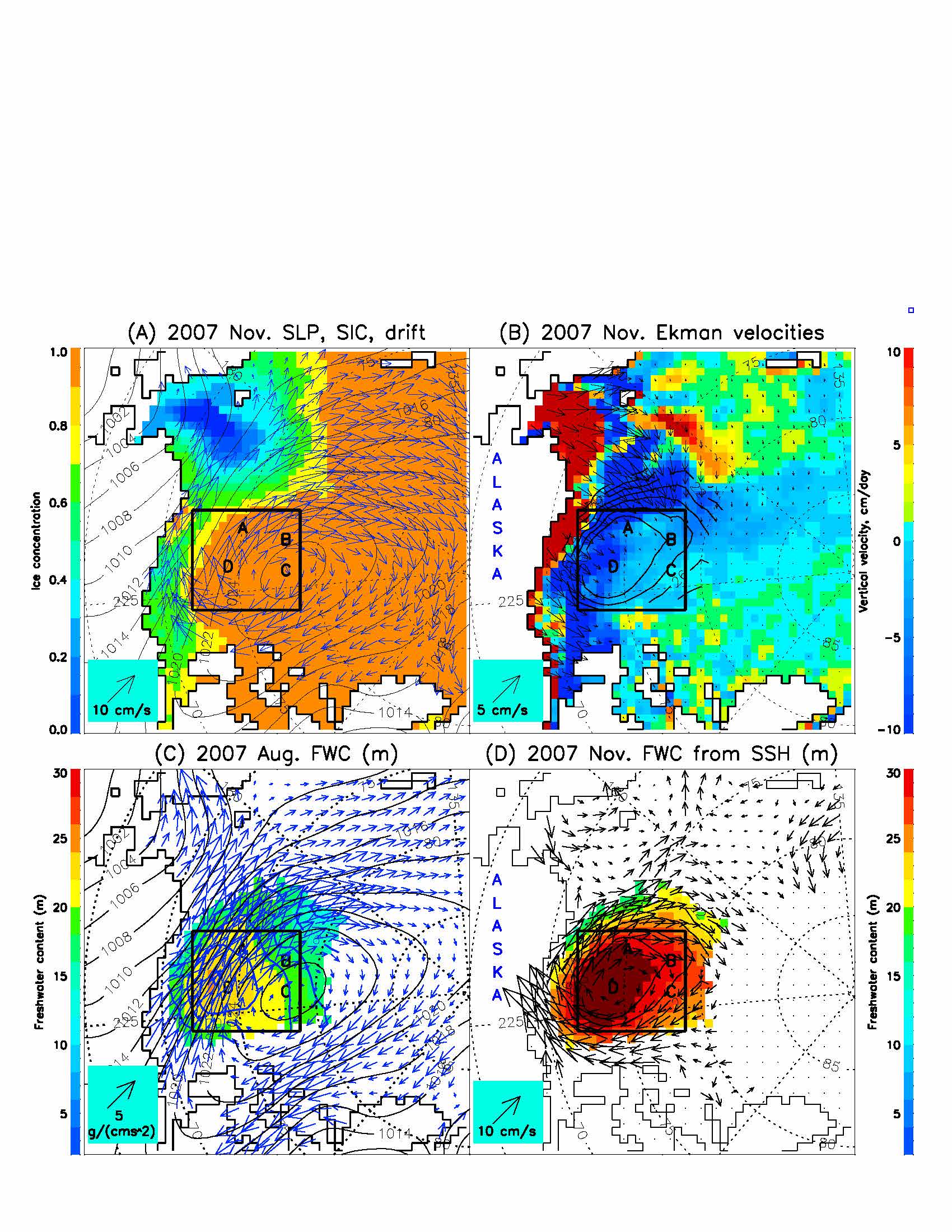


Figure S1b Same as Figure S1a but for November 2007


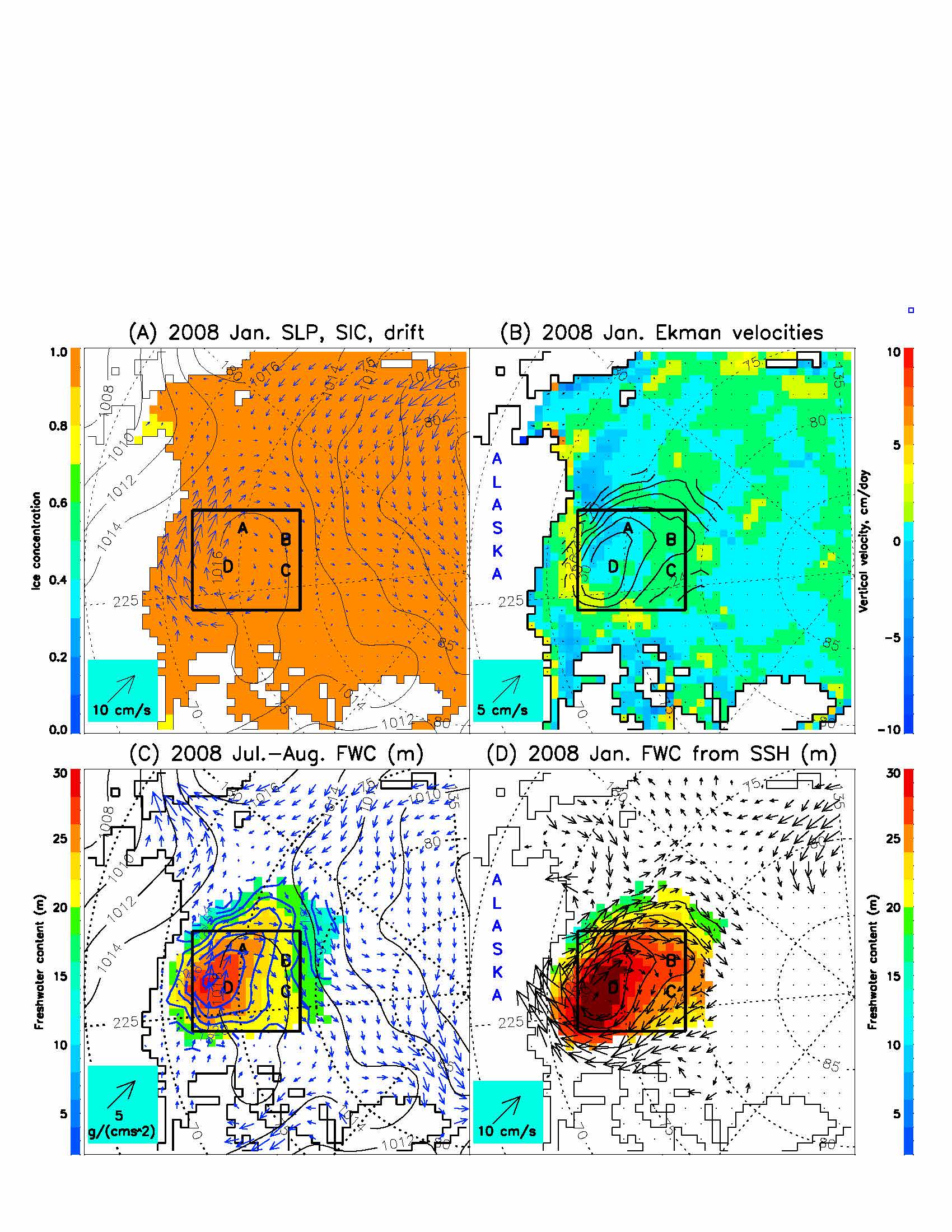


Figure S1c Same as Figure S1a but for January 2008.


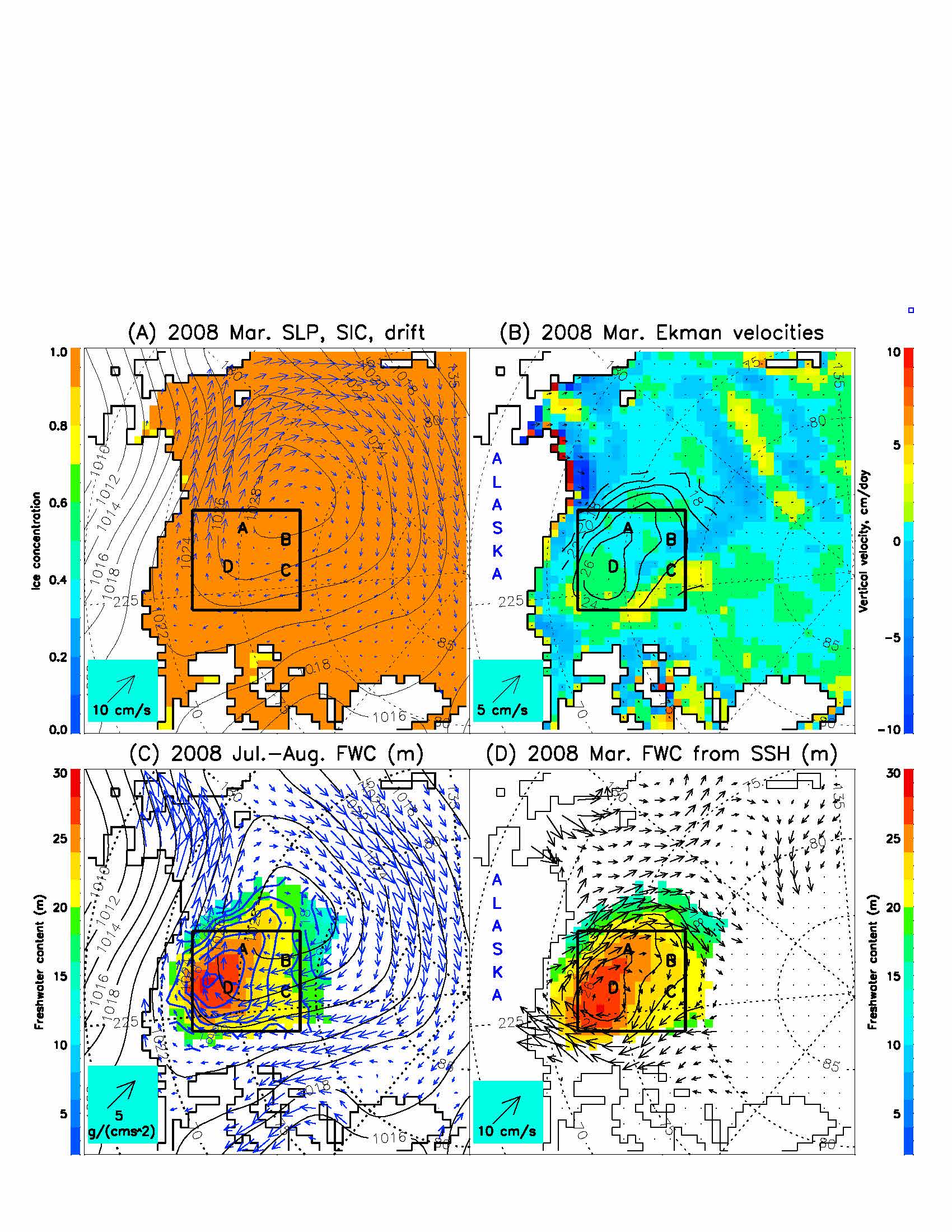


Figure S1d Same as Figure S1a but for March 2008


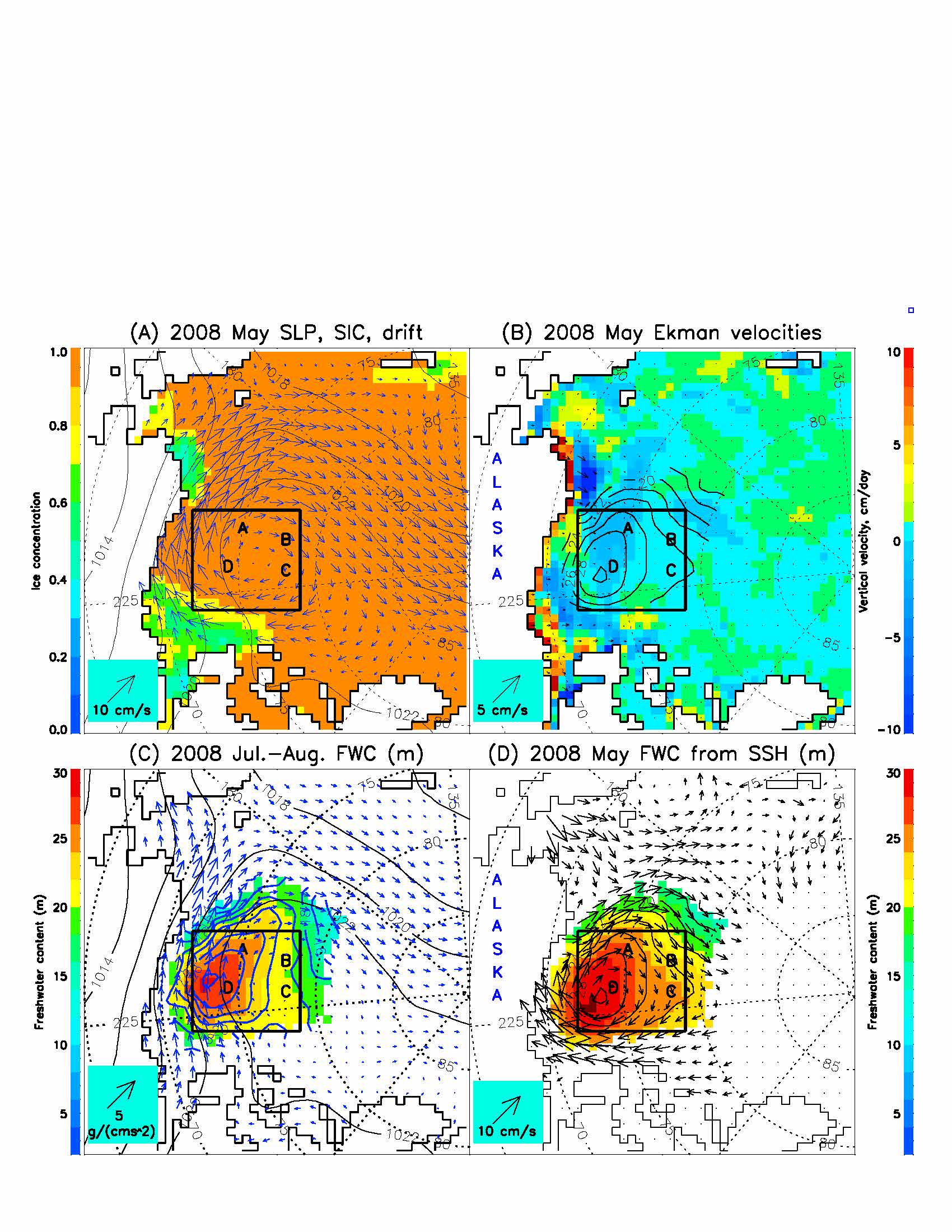


Figure S1e Same as Figure S1a but for May 2008


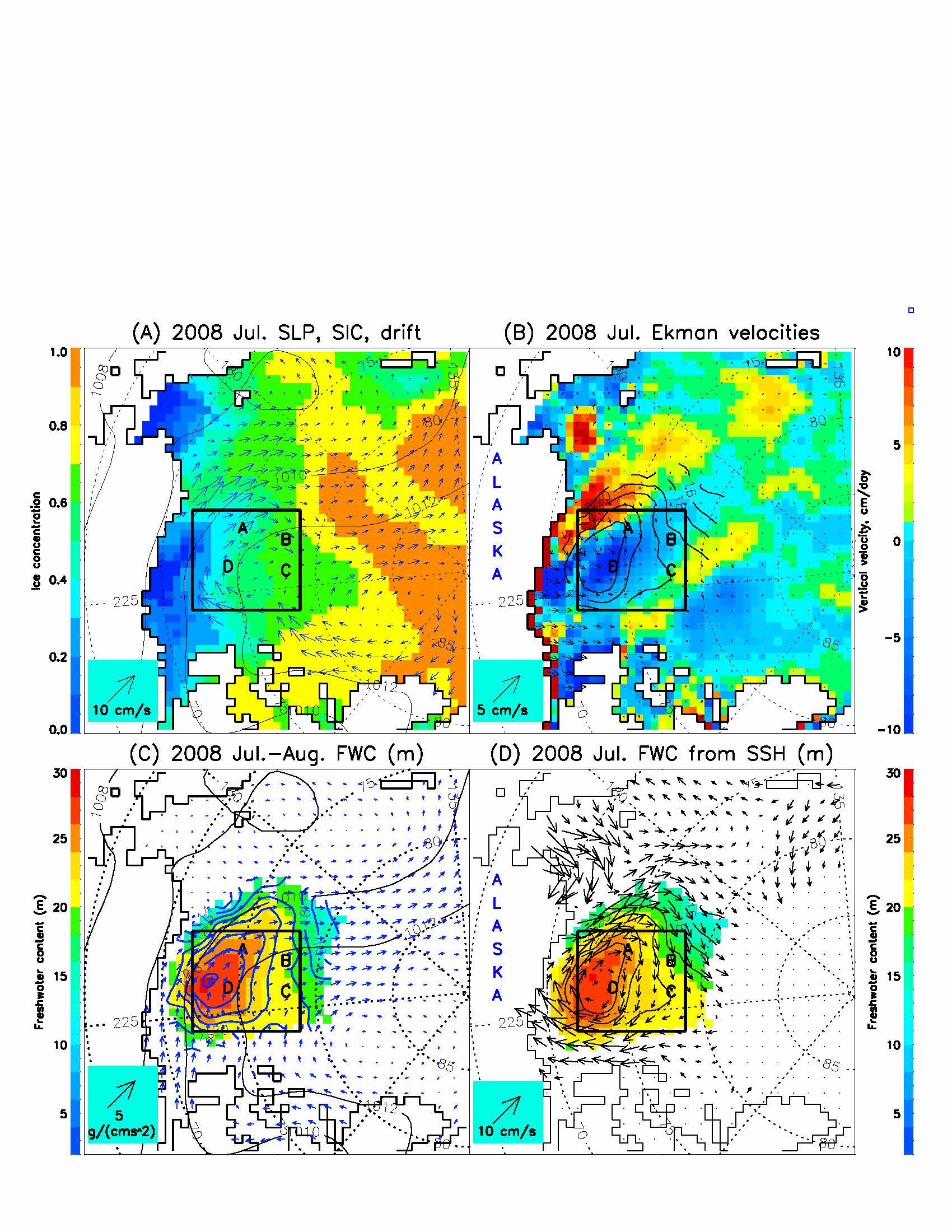


Figure S1f Same as Figure S1a but for July 2008.


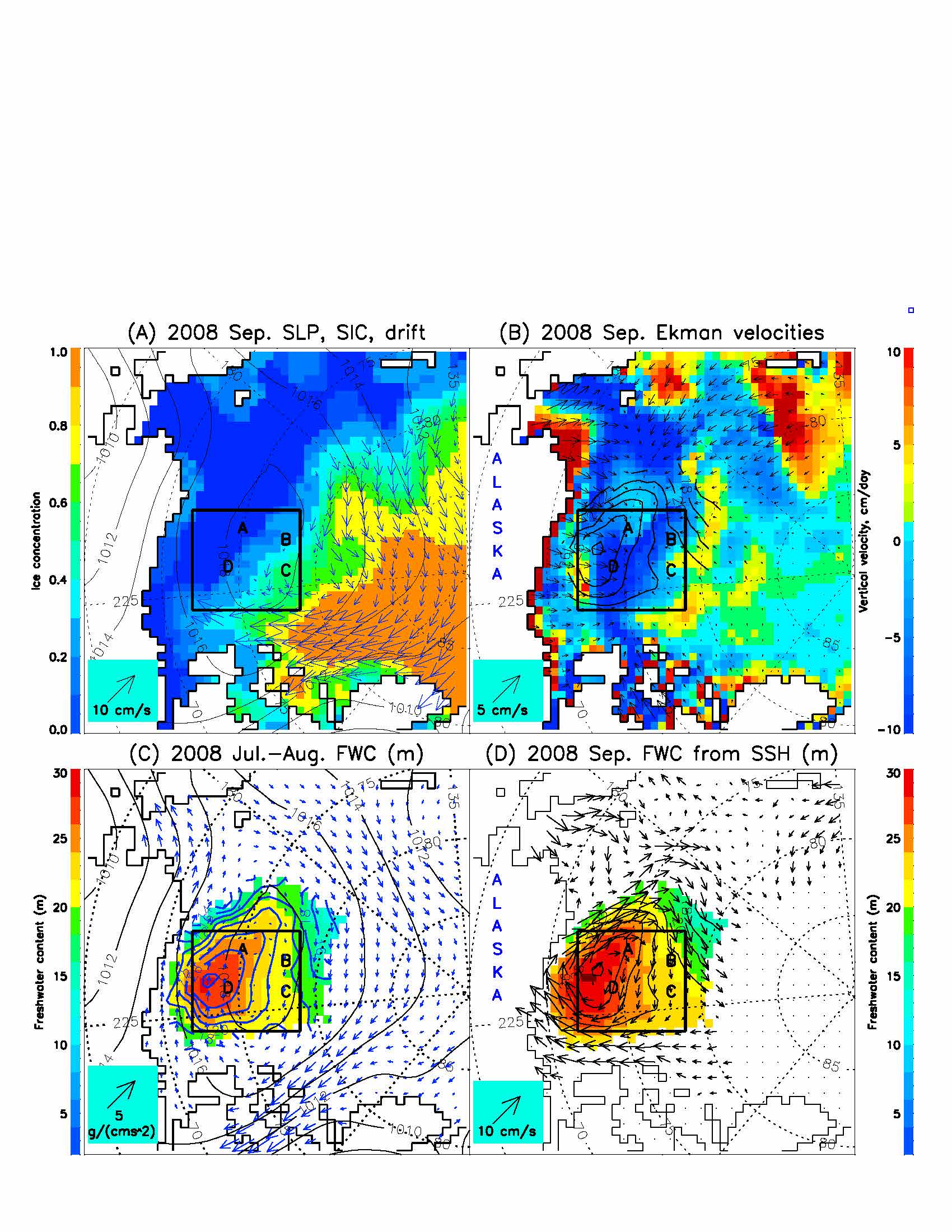


Figure S1g Same as Figure S1a but for September 2008.

- 1. **Supplemental S2**


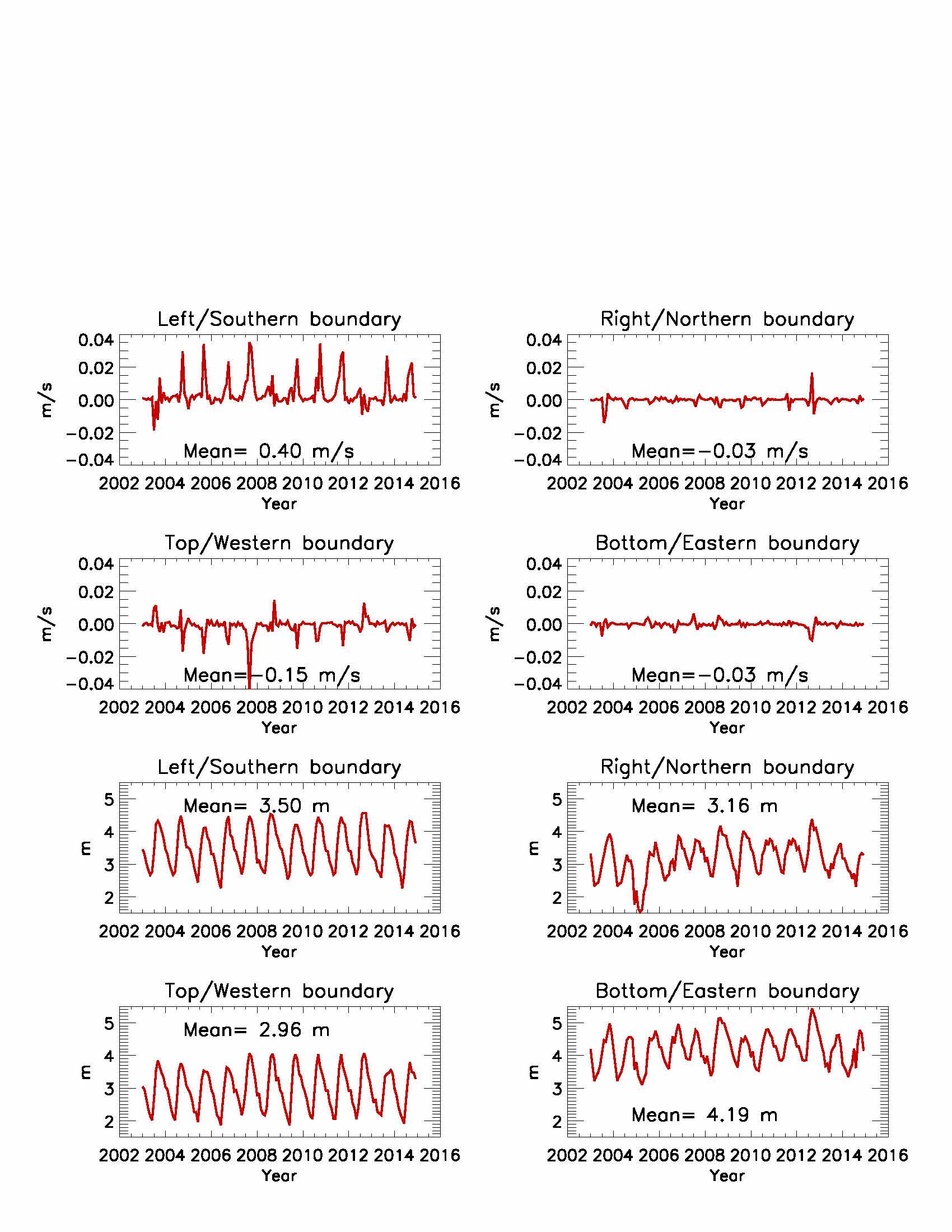


Figure S2 Top four panels: Mean monthly components of Ekman velocities perpendicular to region boundaries (m/s); Bottom four panels: mean monthly freshwater content of the upper 20m layer along boundaries (m)

- 1. **Supplemental S3**

***
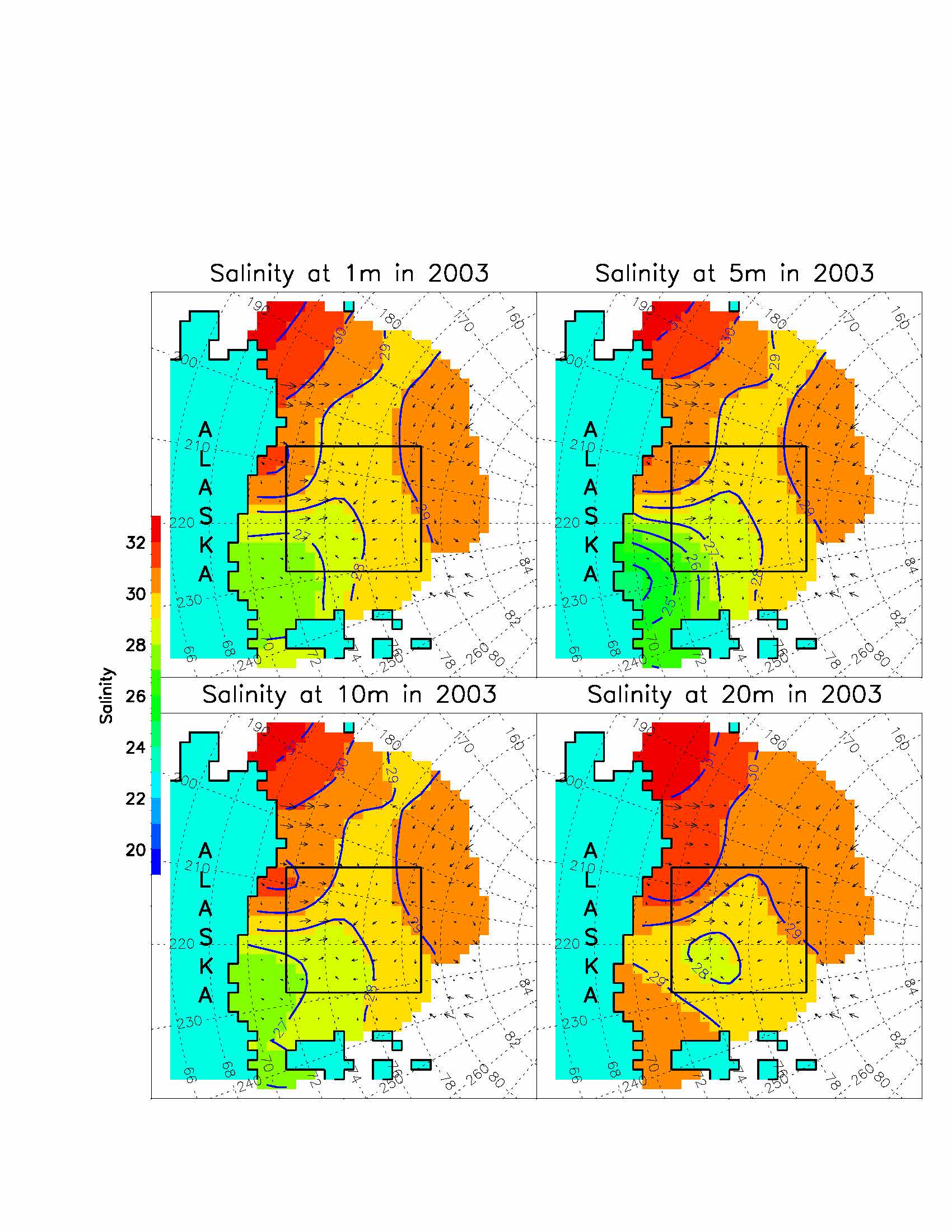
***

Figure S3a Surface water salinity (contour lines and colors) at 1, 5, 10 and 20m observed during hydrographic surveys of the Beaufort Gyre region in 2003. Arrows show Ekman transport in the region (see section 2.5). Black lines bound our rectangular region where freshwater content budget was calculated.


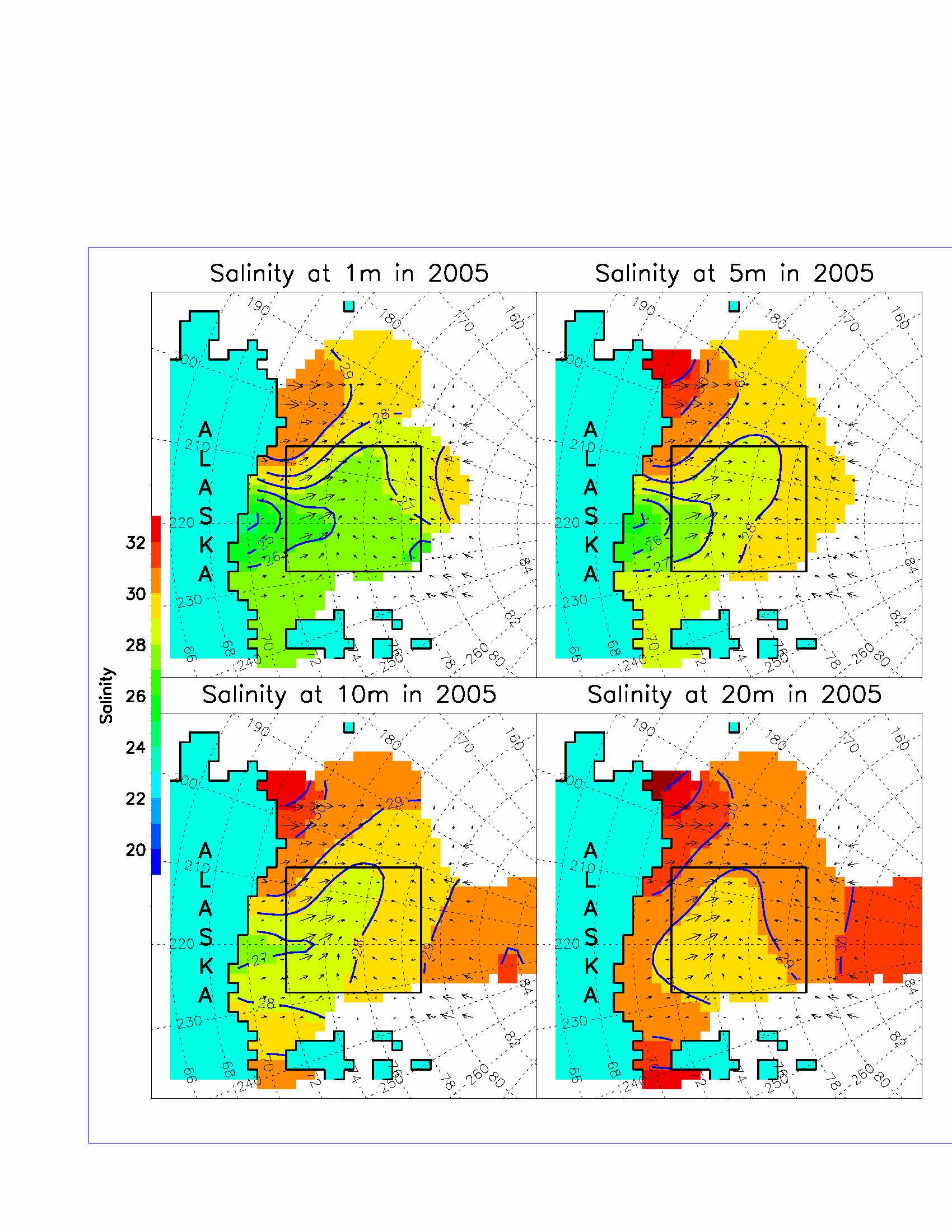


Figure S3b Same as S3a but for 2005.

***
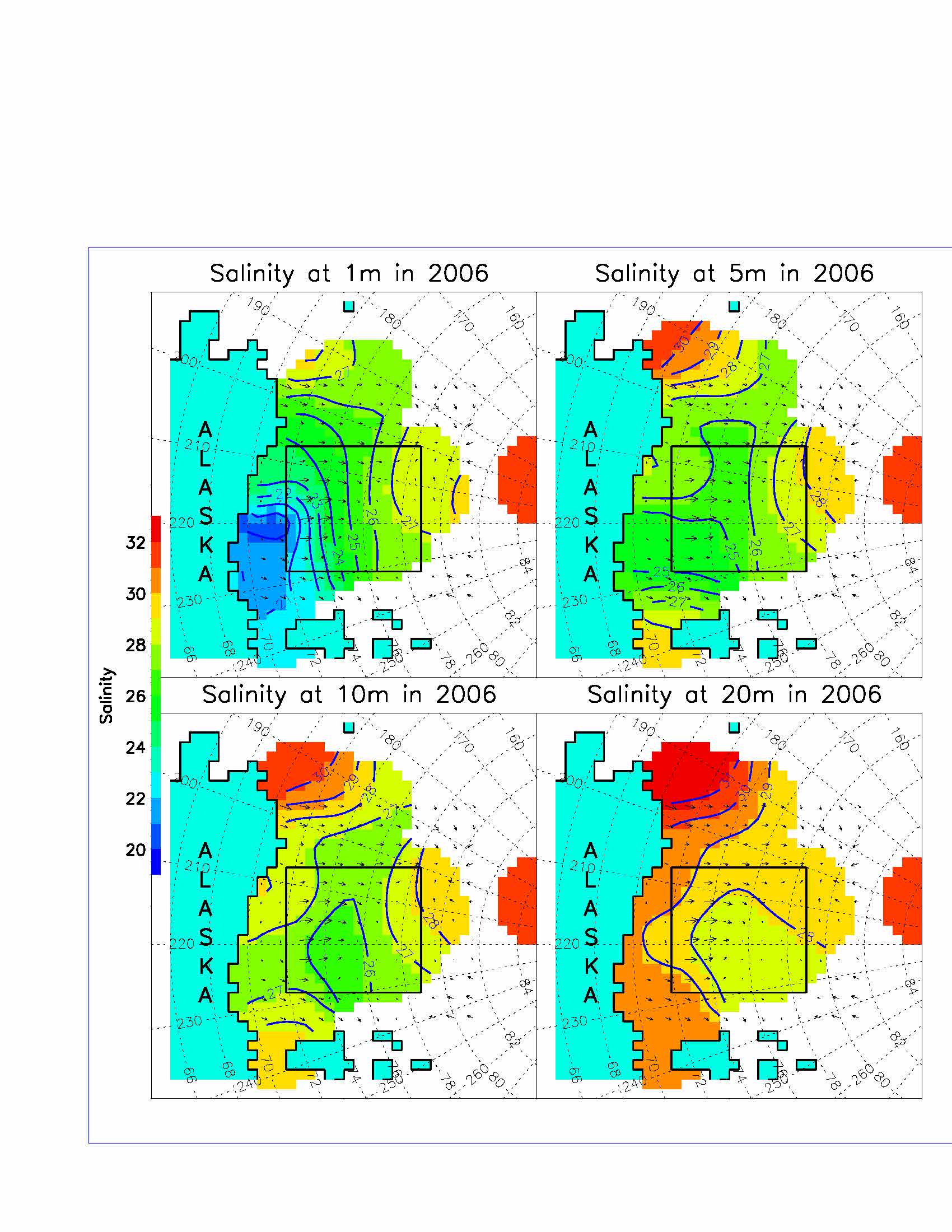
***

Figure S3c Same as S3a but for 2006.

***
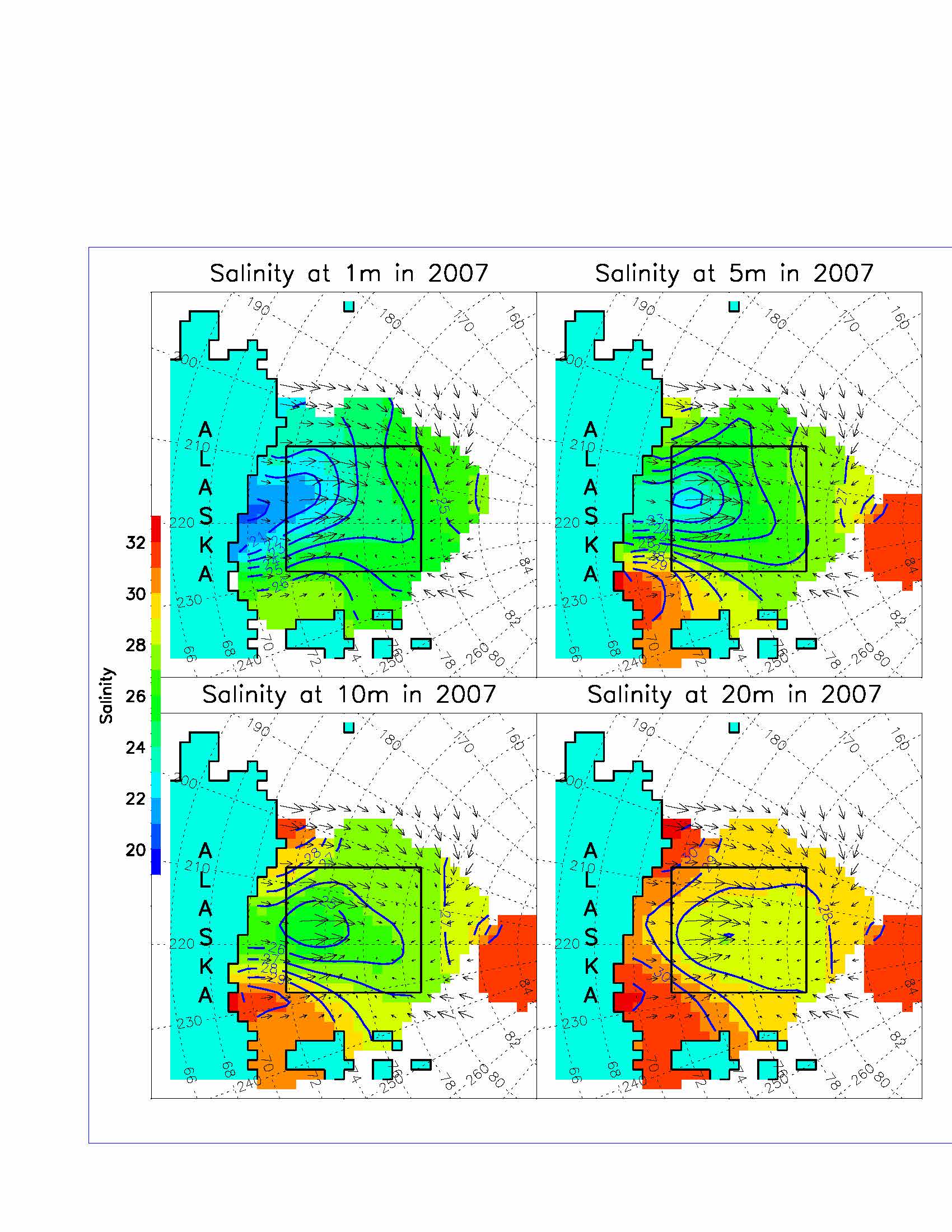
***

Figure S3d Same as S3a but for 2007.

***
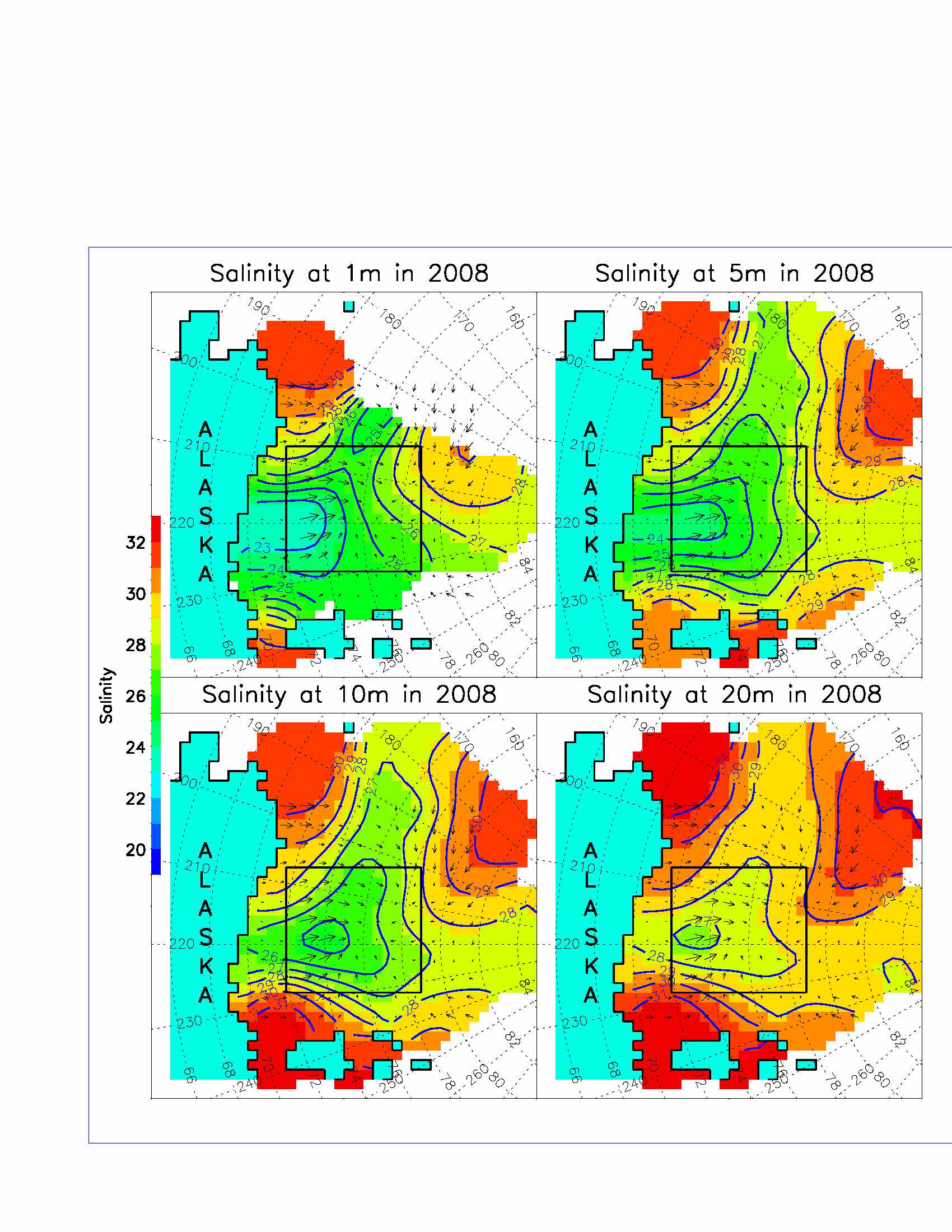
***

Figure S3e Same as S3a but for 2008.

***
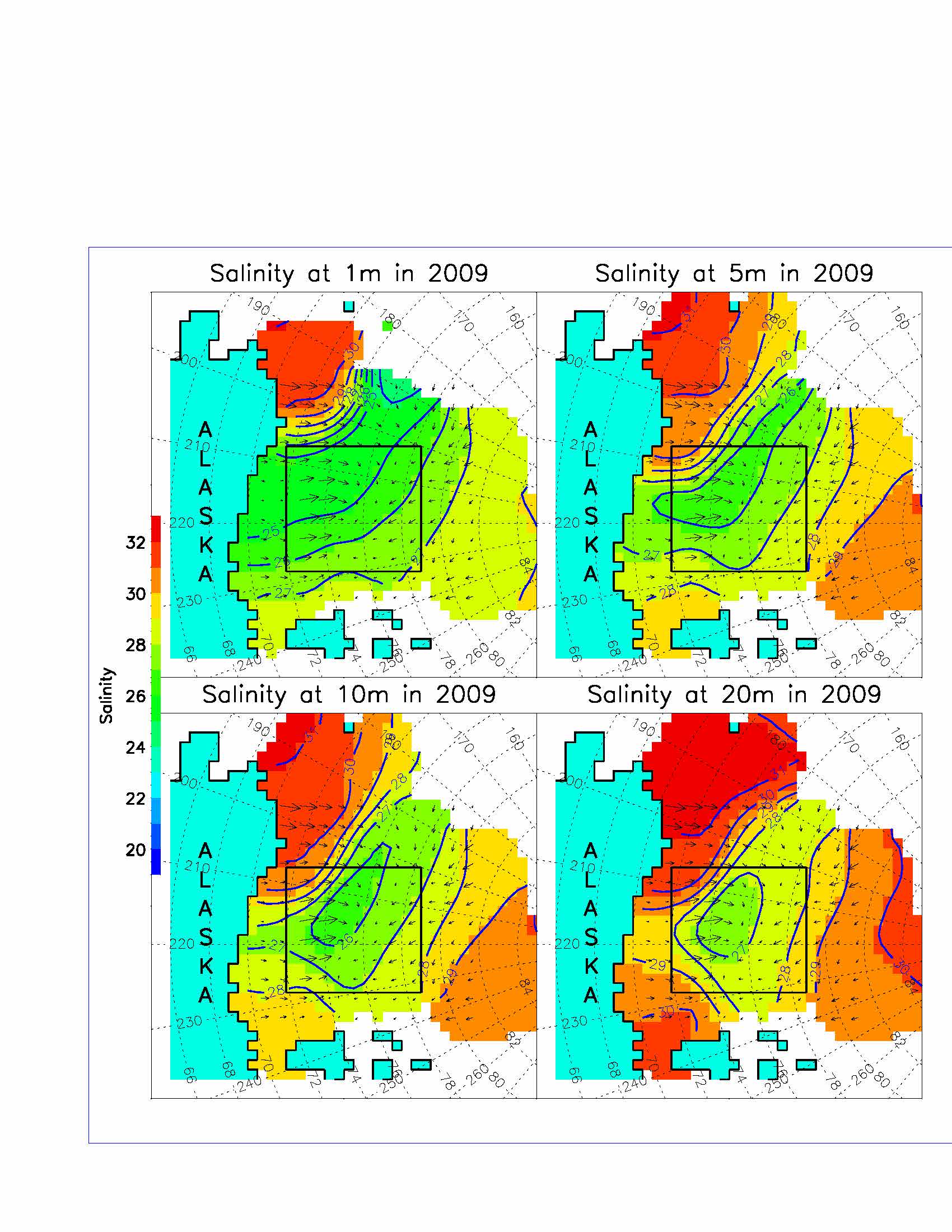
***

Figure S3f Same as S3a but for 2009.

***
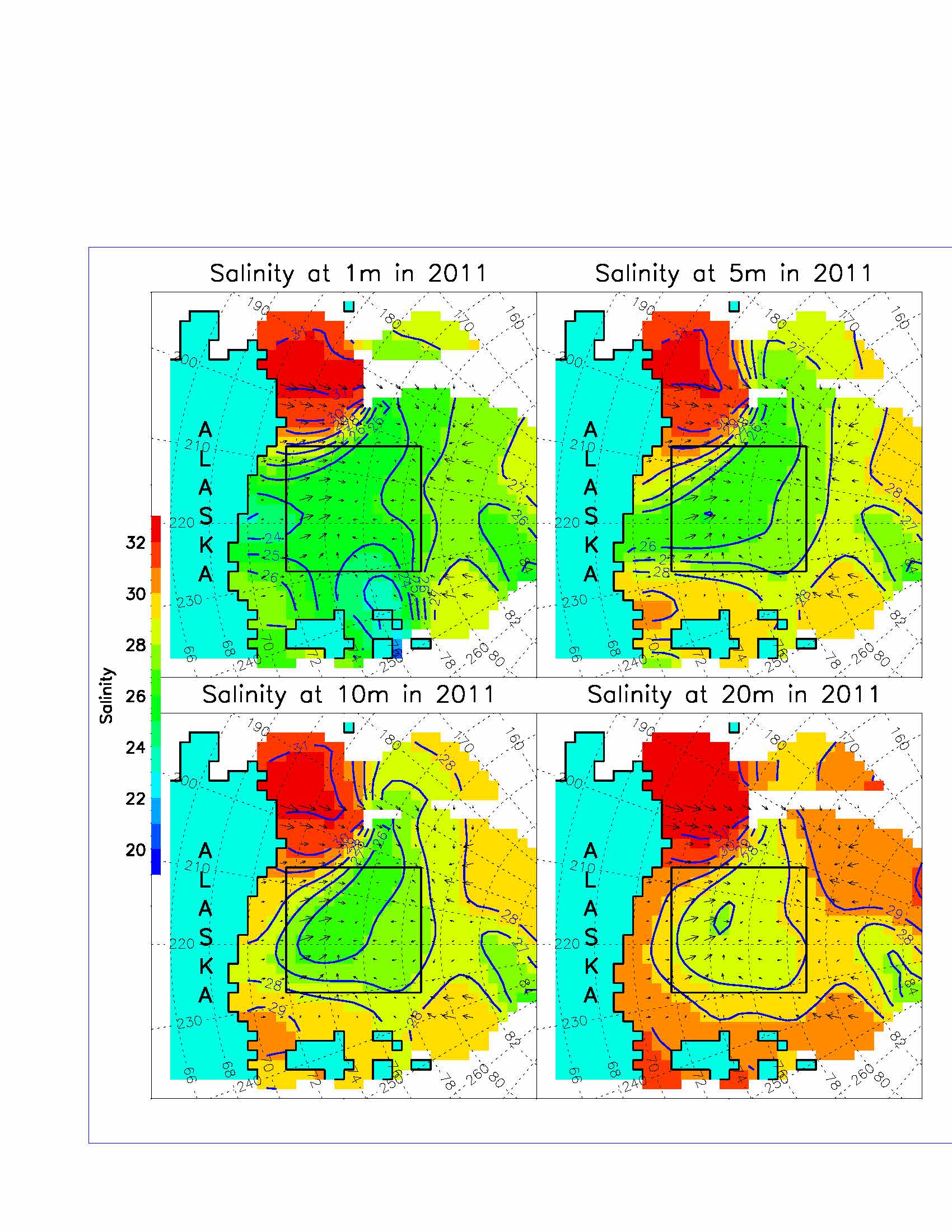
***

Figure S3g Same as S3a but for 2011.

***
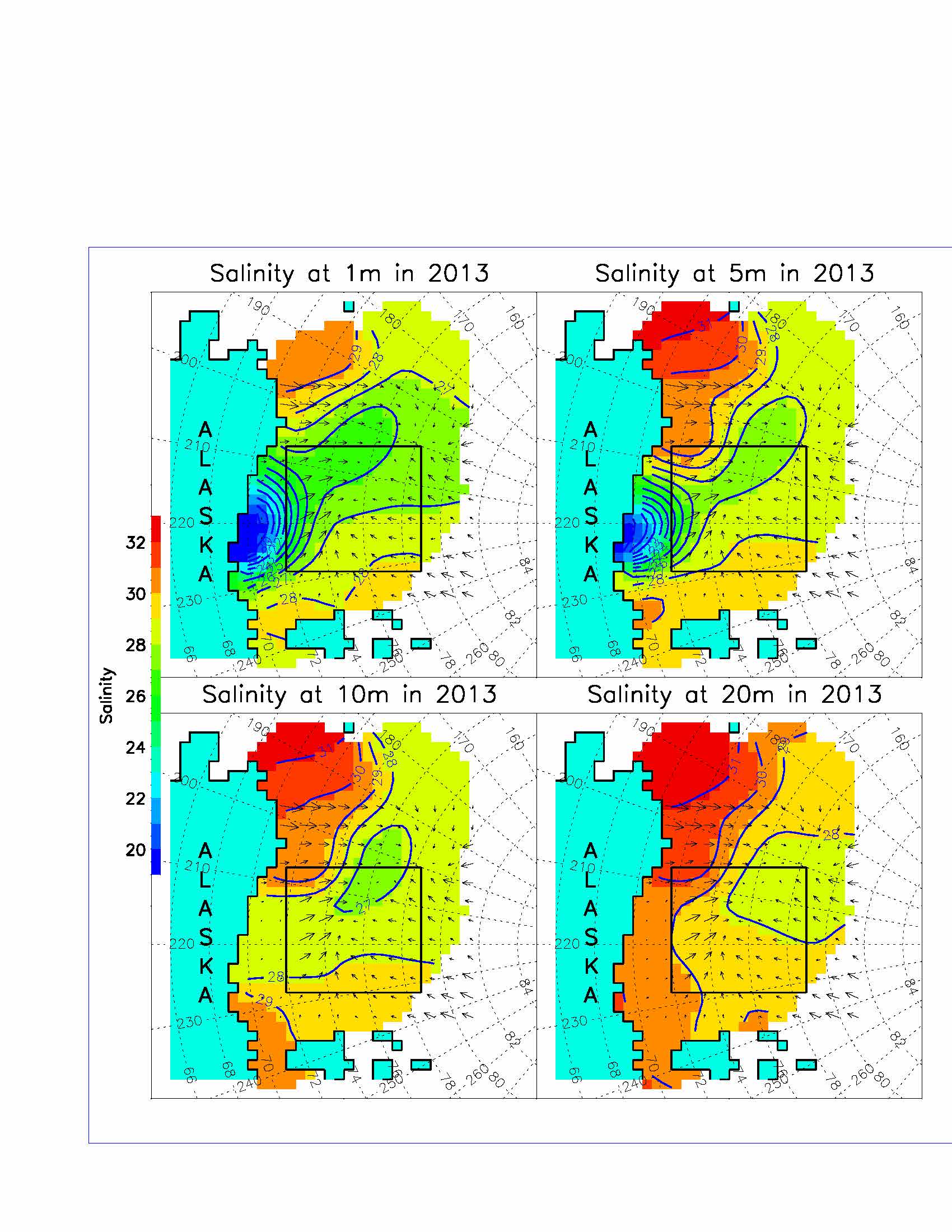
***

Figure S3h Same as S3a but for 2013.

- 1. **Supplemental S4**


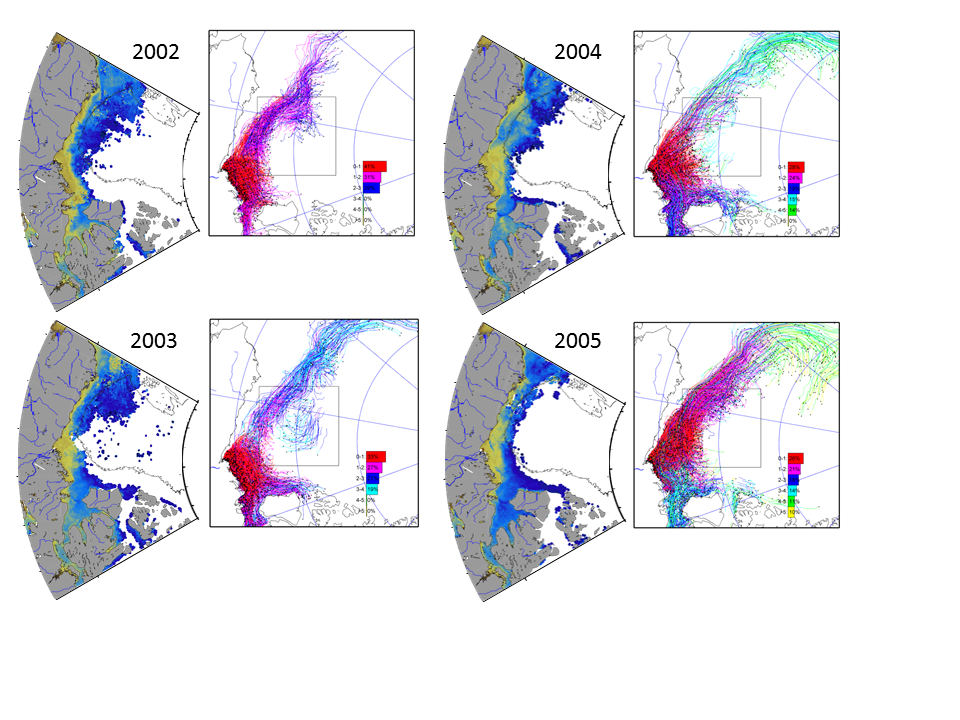


**Figure S4a** Left: The Mackenzie River origin water outflow observed in 2002-2005 (from Fichot et al., 2013). Colors depict distribution of terrigenous dissolved organic matter (tDOM) as a surface manifestation of Mackenzie River water westward propagation (yellow is high concentration and blue is low).  MODIS *Aqua* ocean color 4km resolution satellite data were used for analysis. The contour line represents the 2000-m isobath and outlines the Canada Basin. Right: Distribution of floats released since 2000 at the Mackenzie River mouth and their trajectories at the end of 2002, 2003, 2004 and 2005. Colors of trajectories and legend show percentage of floats at different depths.  Results are from the SibCIOM model (see section 2.6 and supporting information S1). Black bounded region is the same as shown in Figure 2 and 7.


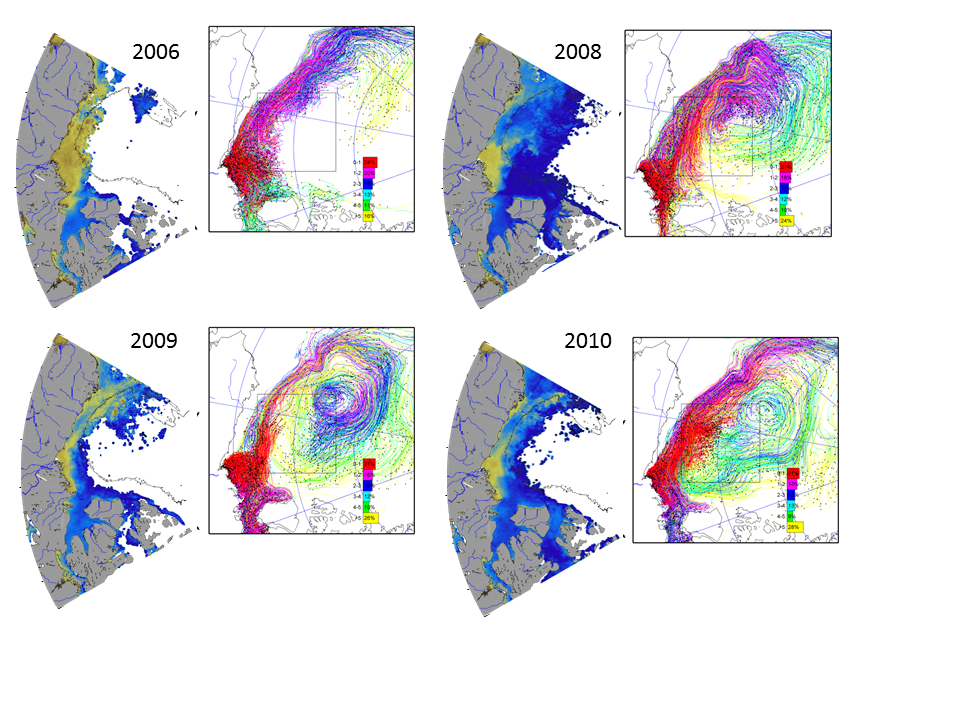


**Figure S4b** Left: The Mackenzie River origin water outflow observed in 2006, 2008, 2009 and 2010 (from Fichot et al., 2013). Colors depict distribution of terrigenous dissolved organic matter (tDOM) as a surface manifestation of Mackenzie River water westward propagation (yellow is high concentration and blue is low).  MODIS *Aqua* ocean color 4km resolution satellite data were used for analysis. The contour line represents the 2000-m isobath and outlines the Canada Basin. Right: Distribution of floats released since 2000 at the Mackenzie River mouth and their trajectories at the end of 2006, 2008, 2009 and 2010. Colors of trajectories and legend show percentage of floats at different depths.  Results are from the SibCIOM model (see section 2.6 and supporting information S1). Black bounded region is the same as shown in Figure 2 and 7.

1. **Supplemental S5: Annual and monthly data for Figures 2 and 3**

**3.1 S5.1 Figure 2 bottom: Annual FWC from moorings**

**Year FWC (x1.E3 km^3^)**

2003 6.47500

2004 7.20278

2005 8.88194

2006 9.13820

2007 9.71945

2008 13.1292

2009 11.9917

2010 12.4528

2011 13.1000

2012 12.4183

2013 11.6778

2014 12.3187

2015 12.5833

2016 14.1367

2017 14.4751

1. 14.5811

**3.2 S5.2 Figure 3, top panel: FWC from hydrography**

**Year month FWC (x1.E3 km^3^) Error (x1.E3 km^3^)**

2003 8.0 16.9 1.3

2004 8.0 17.2 1.6

2005 8.0 18.2 1.7

2006 8.0 18.7 1.5

2007 8.0 19.8 1.5

2008 7.5 21.8 1.3

2009 9.5 21.8 1.7

2010 9.5 21.9 1.6

2011 7.5 21.8 1.9

2012 8.0 22.1 1.4

2013 8.0 20.6 1.4

2014 9.5 21.4 1.5

2015 9.5 22.3 1.6

2016 9.5 23.2 1.6

2017 9.0 23.4 1.7

2018 8.0 23.3 1.7

**3.3 S5.3 Figure 3, top panel: FWC from ITPs**

**Year FWC(x1.E3 km^3^)**

2004.58 15.8000

2004.67 16.3000

2005.67 17.8000

2005.75 18.1000

2005.83 18.4000

2005.92 19.0000

2006.00 19.2000

2006.08 19.2000

2006.17 19.9000

2006.25 19.4000

2006.33 19.3000

2006.42 18.9000

2006.50 18.4000

2006.58 18.8000

2006.67 21.1000

2006.75 20.7000

2006.83 20.9000

2006.92 20.6000

2007.00 21.0000

2007.08 21.3000

2007.17 21.4000

2007.25 20.8000

2007.33 21.2000

2007.42 21.0000

2007.50 21.5000

2007.58 22.9000

2008.17 25.4000

2008.25 25.5000

2008.33 25.1000

2008.42 24.2000

2008.50 23.8000

2008.67 22.1000

2008.92 21.2000

2009.08 20.2000

2009.42 20.5000

2009.75 22.3000

2009.83 25.4000

2009.92 25.5000

2010.00 26.7000

2010.08 24.7000

2010.17 24.6000

2010.33 25.1000

2010.58 25.4000

2010.67 24.1000

2010.75 23.9000

2010.83 26.6000

2010.92 26.4000

2011.00 27.4000

2011.08 26.6000

2011.58 23.7000

2011.67 23.7000

2011.75 24.8000

2011.83 23.9000

2011.92 24.5000

2012.00 25.7000

2012.08 24.8000

2012.17 23.8000

2012.25 23.5000

2012.33 22.7000

2012.75 22.8000

2012.83 21.6000

2012.92 20.7000

2013.00 20.7000

2013.08 20.7000

2013.17 22.6000

2013.25 23.8000

2013.33 23.0000

2013.42 22.0000

2013.58 21.9000

2013.67 23.4000

2013.75 25.0000

2013.83 24.7000

2013.92 24.3000

2014.00 24.2000

2014.08 24.6000

2014.17 21.0000

2014.25 20.4000

2014.33 21.3000

2014.42 22.5000

2014.50 23.7000

2014.58 23.8000

2014.67 23.9000

2014.75 25.2000

2014.83 25.3000

2014.92 26.5000

2015.00 26.2000

2015.08 24.9000

2015.92 22.4000

2016.00 23.0000

2016.08 25.9000

2016.17 27.9000

2016.25 27.9000

2016.75 22.3000

2016.83 23.5000

2016.92 22.5000

2017.00 23.0000

2017.42 24.2000

2017.50 24.4000

2018.08 23.6000

2018.17 24.7000

2018.25 25.1000

2018.33 25.7000

2018.42 25.7000

2018.67 24.2000

2018.75 23.9000

2018.83 23.4000

- 1. 24.9000

**3.4 S5.4 Figure 3, top panel: FWC inferred from SSH**

**FWC1 – FWC in the BG region; FWC2 – FWC based on data at mooring locations (x1.E3 km^3^)**

**Year FWC1 FWC2**

2003.75 17.3300 18.0575

2003.83 17.6200 18.5575

2003.92 18.8800 19.4825

2004.00 19.9000 20.8400

2004.08 20.2900 21.1350

2004.17 21.4300 22.1850

2004.25 19.8700 20.1925

2004.33 19.3200 20.0125

2004.42 18.6600 19.4950

2004.50 19.8000 20.9850

2004.58 18.3500 19.1200

2004.67 17.5500 18.0350

2004.75 17.7700 17.9550

2004.83 18.5800 19.5225

2004.92 19.6100 20.4200

2005.00 20.3400 21.2825

2005.08 20.6600 21.1375

2005.17 21.4000 22.0650

2005.25 21.2600 22.0075

2005.33 19.9300 20.6800

2005.42 19.1200 20.2850

2005.50 19.8500 21.1575

2005.58 19.2500 20.6175

2005.67 19.2100 20.3000

2005.75 18.8400 19.8750

2005.83 19.4900 20.5475

2005.92 20.2100 20.6825

2006.00 21.0300 21.5300

2006.08 21.3800 22.2050

2006.17 20.8400 21.5575

2006.25 20.8300 22.1125

2006.33 19.7600 21.0725

2006.42 17.7900 19.2525

2006.50 19.2700 20.8275

2006.58 18.7100 19.9400

2006.67 17.7800 18.9125

2006.75 20.0400 21.3800

2006.83 21.0700 22.7750

2006.92 21.3000 22.8975

2007.00 22.6900 24.3025

2007.08 23.5400 25.2575

2007.17 23.0600 24.9325

2007.25 23.4400 25.2400

2007.33 21.9100 23.8025

2007.42 21.0200 22.8425

2007.50 22.1700 24.2925

2007.58 20.4400 22.0075

2007.67 22.4000 24.1300

2007.75 22.2400 24.4375

2007.83 22.8900 26.0700

2007.92 24.1900 27.6875

2008.00 25.3200 28.6550

2008.08 26.7300 29.5475

2008.17 26.6400 28.9125

2008.25 26.5300 28.7725

2008.33 25.0000 27.4225

2008.42 23.5200 25.7600

2008.50 24.0100 26.2525

2008.58 22.1100 23.8350

2008.67 21.6000 23.0325

2008.75 22.1400 23.6950

2008.83 23.0400 24.5275

2008.92 23.6400 25.2650

2009.00 23.6100 25.2950

2009.08 24.4800 25.7775

2009.17 25.0000 26.4550

2009.25 24.4900 25.9525

2009.33 23.6400 25.1875

2009.42 21.7500 23.1775

2009.50 22.0300 23.6575

2009.58 21.3300 23.1375

2009.67 20.7000 22.1650

2009.75 19.4200 21.0600

2009.83 22.2800 24.1400

2009.92 24.0000 25.6500

2010.00 24.5600 25.8700

2010.08 25.8100 27.1700

2010.17 26.0300 27.6850

2010.25 25.1800 26.7050

2010.33 23.8500 25.5650

2010.42 22.8700 25.0250

2010.50 23.6300 26.0625

2010.58 22.6200 24.3150

2010.67 23.0900 25.0075

2010.75 23.5100 25.3825

2010.83 24.0300 25.8200

2010.92 24.2000 26.0575

2011.00 24.8800 26.7075

2011.08 26.0800 28.1725

2011.17 25.5700 27.2575

2011.25 25.8900 27.7750

2011.33 24.4600 26.1975

2011.42 22.9200 24.6975

2011.50 23.3800 25.5675

2011.58 21.9100 23.8600

2011.67 22.5500 24.4125

2011.75 22.7900 24.3850

2011.83 23.4400 25.8050

2011.92 24.2400 26.2025

2012.00 24.8800 26.1475

2012.08 25.4400 26.7475

2012.17 26.0600 27.3525

2012.25 25.4800 26.9075

2012.33 23.5500 25.2150

2012.42 22.5700 24.2850

2012.50 23.8800 25.7125

2012.58 22.8300 24.4300

2012.67 22.4600 23.9375

2012.75 23.2600 24.4400

2012.83 22.6200 23.9200

2012.92 22.7800 24.0375

2013.00 23.4400 24.7225

2013.08 23.6800 25.0125

2013.17 24.0000 25.1900

2013.25 23.4700 25.2125

2013.33 22.1000 23.6725

2013.42 20.7900 22.2675

2013.50 21.7100 23.3125

2013.58 22.2100 24.1250

2013.67 22.6500 24.2625

2013.75 22.6800 24.5600

2013.83 23.5900 25.6425

2013.92 23.5900 25.3225

2014.00 23.6900 25.3600

2014.08 24.1700 25.9025

2014.17 24.3500 26.1350

2014.25 23.9100 25.7400

2014.33 23.0800 24.9850

2014.42 21.6600 23.6125

2014.50 22.5400 24.4100

2014.58 22.7900 24.7225

2014.67 22.8100 24.8150

2014.75 23.1200 25.2400

2014.83 23.4800 25.6100

- 1. 23.4100 25.3700

**3.5 S5.5 Figure 3, top panel: FWC from sea ice**

**Year FWC (x1E3 km^3^)**

2003.75 0.777000

2003.83 0.538333

2003.92 0.653333

2004.00 0.884333

2004.08 1.18567

2004.17 1.47533

2004.25 1.77667

2004.33 2.21000

2004.42 2.29600

2004.50 2.51733

2004.58 2.36567

2004.67 1.86767

2004.75 1.39767

2004.83 1.01733

2004.92 1.20900

2005.00 1.31267

2005.08 1.61867

2005.17 2.03467

2005.25 2.23533

2005.33 2.31700

2005.42 2.54033

2005.50 2.55333

2005.58 2.62500

2005.67 2.39567

2005.75 1.85300

2005.83 1.32900

2005.92 1.37400

2006.00 1.29050

2006.08 1.45300

2006.17 1.75650

2006.25 1.89500

2006.33 2.16750

2006.42 2.27000

2006.50 2.51850

2006.58 2.32600

2006.67 1.79650

2006.75 1.16600

2006.83 1.08175

2006.92 1.27425

2007.00 1.31900

2007.08 1.55075

2007.17 1.59625

2007.25 1.80000

2007.33 2.07175

2007.42 2.32100

2007.50 2.25050

2007.58 2.12650

2007.67 1.50675

2007.75 0.98650

2007.83 0.60225

2007.92 0.96625

2008.00 1.56675

2008.08 1.39800

2008.17 1.58375

2008.25 1.79125

2008.33 1.92600

2008.42 2.04800

2008.50 1.91225

2008.58 1.97150

2008.67 1.27075

2008.75 0.25300

2008.83 0.11600

2008.92 0.35700

2009.00 0.66500

2009.08 0.84133

2009.17 1.18567

2009.25 1.36733

2009.33 1.60900

2009.42 1.63733

2009.50 1.76133

2009.58 1.72900

2009.67 1.34333

2009.75 0.57533

2009.83 0.16366

2009.92 0.52450

2010.00 0.70950

2010.08 1.04850

2010.17 1.30400

2010.25 1.49350

2010.33 1.73800

2010.42 2.07450

2010.50 2.55250

2010.58 2.41100

2010.67 1.50650

2010.75 0.57550

2010.83 0.28450

2010.92 0.45333

2011.00 0.65266

2011.08 0.90733

2011.17 1.33200

2011.25 1.37900

2011.33 1.69367

2011.42 1.77467

2011.50 2.01500

2011.58 1.83933

2011.67 1.34367

2011.75 0.56833

2011.83 0.26000

2011.92 0.54300

2012.00 0.75700

2012.08 0.95400

2012.17 1.32167

2012.25 1.53733

2012.33 1.72433

2012.42 1.87267

2012.50 1.95733

2012.58 1.92067

2012.67 0.99100

2012.75 0.13166

2012.83 0.00800

2012.92 0.06066

2013.00 0.39333

2013.08 0.65600

2013.17 0.98466

2013.25 1.18300

2013.33 1.39533

2013.42 1.69733

2013.50 1.84467

2013.58 1.74500

2013.67 1.31567

2013.75 0.83700

2013.83 0.88166

2013.92 1.04900

2014.00 1.19633

2014.08 1.32733

2014.17 1.33467

2014.25 1.65467

2014.33 1.74333

2014.42 1.85033

2014.50 1.85400

2014.58 1.83067

2014.67 1.35567

2014.75 0.79333

2014.83 0.56866

2014.92 0.79166

2015.00 1.02700

2015.08 1.20567

2015.17 1.32067

2015.25 1.59300

2015.33 1.68900

2015.42 1.91733

2015.50 2.06033

2015.58 1.78633

2015.67 1.02033

2015.75 0.38233

2015.83 0.18366

2015.92 0.35033

2016.00 0.61266

2016.08 0.88000

2016.17 1.18233

2016.25 1.36767

2016.33 1.48300

2016.42 1.58967

2016.50 1.61867

2016.58 1.30900

2016.67 0.76600

2016.75 0.19666

2016.83 0.20000

2016.92 0.67000

2017.00 0.89000

2017.08 0.95000

2017.17 1.29000

2017.25 1.47000

2017.33 1.33000

2017.42 1.63000

2017.50 1.90000

2017.58 0.25000

2017.67 0.08000

2017.75 0.19666

2017.83 0.37000

2017.92 0.74000

2018.00 1.14000

2018.08 1.08000

2018.17 1.34000

2018.25 1.70000

2018.33 1.65000

2018.42 1.70000

2018.50 1.13000

- 1. 0.32000

**3.6 S5.6 Figure 3, top panel: FWC from model results**

**Year FWC (1.E3 km^3^)**

2000.00 17.7300

2000.08 17.5610

2000.17 17.4000

2000.25 17.2540

2000.33 17.1460

2000.42 17.2500

2000.50 17.6690

2000.58 17.9190

2000.67 18.0140

2000.75 17.9680

2000.83 17.8240

2000.92 17.7180

2001.00 17.5850

2001.08 17.4390

2001.17 17.2440

2001.25 17.0910

2001.33 16.9870

2001.42 17.0710

2001.50 17.4330

2001.58 17.6800

2001.67 17.7340

2001.75 17.6550

2001.83 17.6150

2001.92 17.5400

2002.00 17.4060

2002.08 17.2420

2002.17 17.0980

2002.25 16.9830

2002.33 16.9500

2002.42 17.0900

2002.50 17.4400

2002.58 17.6540

2002.67 17.6700

2002.75 17.6520

2002.83 17.5040

2002.92 17.3830

2003.00 17.2480

2003.08 17.1310

2003.17 17.0220

2003.25 16.9140

2003.33 16.8670

2003.42 17.0010

2003.50 17.4110

2003.58 17.6170

2003.67 17.6910

2003.75 17.6730

2003.83 17.4990

2003.92 17.3520

2004.00 17.2870

2004.08 17.1580

2004.17 17.0130

2004.25 16.8890

2004.33 16.8320

2004.42 17.0140

2004.50 17.5000

2004.58 17.9110

2004.67 18.0050

2004.75 18.1220

2004.83 18.1300

2004.92 18.0670

2005.00 17.8960

2005.08 17.7610

2005.17 17.6450

2005.25 17.5290

2005.33 17.4720

2005.42 17.6190

2005.50 18.0540

2005.58 18.4540

2005.67 18.6780

2005.75 19.0200

2005.83 18.9800

2005.92 18.7160

2006.00 18.5420

2006.08 18.4090

2006.17 18.2600

2006.25 18.1090

2006.33 18.0530

2006.42 18.2420

2006.50 18.7100

2006.58 19.0340

2006.67 19.1810

2006.75 19.2980

2006.83 19.1660

2006.92 19.0270

2007.00 18.8600

2007.08 18.7120

2007.17 18.5730

2007.25 18.5000

2007.33 18.4660

2007.42 18.6790

2007.50 19.2140

2007.58 19.7790

2007.67 20.1650

2007.75 20.5780

2007.83 20.7060

2007.92 20.6500

2008.00 20.4720

2008.08 20.3480

2008.17 20.2450

2008.25 20.1160

2008.33 20.0820

2008.42 20.2730

2008.50 20.8390

2008.58 21.1140

2008.67 21.2710

2008.75 21.3430

2008.83 21.2750

2008.92 21.1150

2009.00 20.9710

2009.08 20.7950

2009.17 20.6370

2009.25 20.4940

2009.33 20.4490

2009.42 20.6530

2009.50 21.1440

2009.58 21.4690

2009.67 21.5200

2009.75 21.5840

2009.83 21.5620

2009.92 21.3990

2010.00 21.1470

2010.08 20.9660

2010.17 20.7570

2010.25 20.5880

2010.33 20.5460

2010.42 20.7670

2010.50 21.3020

2010.58 21.7460

2010.67 22.0040

2010.75 22.0360

2010.83 22.0030

2010.92 21.7840

2011.00 21.5460

2011.08 21.3530

2011.17 21.2120

2011.25 21.0990

2011.33 21.0210

2011.42 21.1940

2011.50 21.7310

2011.58 22.0710

2011.67 22.1700

2011.75 22.3400

2011.83 22.3130

2011.92 22.1360

2012.00 21.9670

2012.08 21.8300

2012.17 21.6580

2012.25 21.4700

2012.33 21.3690

2012.42 21.6290

2012.50 22.2190

2012.58 22.5490

2012.67 22.5290

2012.75 22.4360

2012.83 22.2480

2012.92 21.9480

2013.00 21.7050

2013.08 21.5050

2013.17 21.3620

2013.25 21.2110

2013.33 21.1250

2013.42 21.2910

2013.50 21.8010

2013.58 22.1610

2013.67 22.2980

2013.75 22.3100

2013.83 22.1760

2013.92 22.0190

2014.00 21.8540

2014.08 21.6910

2014.17 21.5580

2014.25 21.4500

2014.33 21.4440

2014.42 21.5950

2014.50 21.9230

2014.58 22.2270

2014.67 22.3910

2014.75 22.4400

2014.83 22.4100

2014.92 22.2720

2015.00 22.1360

2015.08 21.9960

2015.17 21.8630

2015.25 21.7370

2015.33 21.6840

2015.42 21.9060

2015.50 22.2990

2015.58 22.4650

2015.67 22.5800

2015.75 22.6520

2015.83 22.6940

2015.92 22.5620

2016.00 22.4220

2016.08 22.3300

2016.17 22.3050

2016.25 22.2730

2016.33 22.3050

2016.42 22.5000

2016.50 22.8800

2016.58 23.1680

2016.67 23.3340

2016.75 23.3500

2016.83 23.3240

2016.92 23.1860

2017.00 23.0380

2017.08 22.9250

2017.17 22.7800

2017.25 22.7010

2017.33 22.6640

2017.42 22.8870

2017.50 23.5210

2017.58 23.9430

2017.67 24.1160

2017.75 24.0870

2017.83 23.8980

2017.92 23.7230

2018.00 23.5510

2018.08 23.3610

2018.17 23.1630

2018.25 23.0040

2018.33 22.9590

2018.42 23.1940

2018.50 23.7280

2018.58 24.0270

2018.67 24.1910

2018.75 24.4840

2018.83 24.3980

- 1. 24.2150

**3.7 S5.7 Figure 3, top panel: FWC at mooring location inferred from SSH**

**YEAR FWC (*1.E3 km^3)**

**999.000 – no data**

2003.67 999.000

2003.75 8.39500

2003.83 9.58100

2003.92 10.5010

2004.00 10.6360

2004.08 10.0450

2004.17 10.8690

2004.25 8.58800

2004.33 7.86000

2004.42 7.82600

2004.50 11.3270

2004.58 7.99200

2004.67 8.32400

2004.75 8.09967

2004.83 12.4460

2004.92 12.4427

2005.00 10.6570

2005.08 8.63033

2005.17 7.82967

2005.25 9.07466

2005.33 9.29666

2005.42 9.06566

2005.50 11.9693

2005.58 10.4703

2005.67 9.48933

2005.75 10.6337

2005.83 13.0338

2005.92 11.9485

2006.00 10.1333

2006.08 11.1740

2006.17 9.12175

2006.25 10.3457

2006.33 9.64450

2006.42 9.33999

2006.50 10.9742

2006.58 9.94349

2006.67 8.94225

2006.75 11.2925

2006.83 15.1283

2006.92 13.8600

2007.00 13.1303

2007.08 12.4583

2007.17 14.5273

2007.25 11.3273

2007.33 11.1303

2007.42 11.8890

2007.50 14.9680

2007.58 11.9543

2007.67 13.1533

2007.75 16.0315

2007.83 19.5573

2007.92 18.5325

2008.00 18.6895

2008.08 14.9490

2008.17 13.9900

2008.25 11.1185

2008.33 12.6740

2008.42 14.1945

2008.50 15.3930

2008.58 13.6090

2008.67 12.5170

2008.75 13.0910

2008.83 12.2680

2008.92 12.6930

2009.00 12.6260

2009.08 12.4635

2009.17 11.3410

2009.25 11.3825

2009.33 11.4300

2009.42 10.5180

2009.50 13.3760

2009.58 11.4770

2009.67 10.8360

2009.75 12.3525

2009.83 16.9880

2009.92 13.6987

2010.00 14.1240

2010.08 14.4227

2010.17 13.2680

2010.25 13.5677

2010.33 11.5350

2010.42 12.7343

2010.50 15.3827

2010.58 11.5543

2010.67 12.6430

2010.75 14.2940

2010.83 17.9480

2010.92 17.1180

2011.00 15.6485

2011.08 16.0300

2011.17 14.5070

2011.25 12.2670

2011.33 12.4370

2011.42 12.7730

2011.50 14.6540

2011.58 13.0680

2011.67 999.000

2011.75 12.4450

2011.83 15.2345

2011.92 15.9910

2012.00 12.1135

2012.08 12.5765

2012.17 11.3860

2012.25 11.4380

2012.33 10.6125

2012.42 9.63950

2012.50 11.0980

2012.58 999.000

2012.67 999.000

2012.75 10.9043

2012.83 11.1910

2012.92 10.3693

2013.00 12.0800

2013.08 12.8243

2013.17 12.4417

2013.25 14.1377

2013.33 12.2883

2013.42 8.35965

2013.50 11.9205

2013.58 11.8705

2013.67 10.5505

2013.75 12.3770

2013.83 14.3553

2013.92 12.5075

2014.00 13.2533

2014.08 13.6630

2014.17 11.4685

2014.25 10.7725

2014.33 11.8785

2014.42 10.8075

2014.50 999.000

2014.58 999.000

2014.67 999.000

2014.75 999.000

- 1. 999.000

**3.8 S5.8 Figure 3, top panel: FWC from moorings below 65m**

**999.000 – no data**

**YEAR FWC (x1.E3 km^3^)**

2003.08 999.000

2003.17 999.000

2003.25 999.000

2003.33 999.000

2003.42 999.000

2003.50 999.000

2003.58 999.000

2003.67 999.000

2003.75 6.20000

2003.83 6.40000

2003.92 6.80000

2004.00 6.50000

2004.08 6.20000

2004.17 6.30000

2004.25 6.70000

2004.33 6.90000

2004.42 7.00000

2004.50 6.70000

2004.58 6.90000

2004.67 6.70000

2004.75 8.10000

2004.83 7.96667

2004.92 8.30000

2005.00 8.66667

2005.08 8.93333

2005.17 8.70000

2005.25 8.50000

2005.33 8.30000

2005.42 8.30000

2005.50 8.33333

2005.58 8.63333

2005.67 8.83333

2005.75 9.22500

2005.83 9.30000

2005.92 9.75000

2006.00 9.77500

2006.08 9.65000

2006.17 9.45000

2006.25 9.15000

2006.33 9.15000

2006.42 9.07500

2006.50 9.00000

2006.58 8.95000

2006.67 9.25000

2006.75 9.05000

2006.83 8.63333

2006.92 8.96667

2007.00 9.33333

2007.08 9.43333

2007.17 9.33333

2007.25 9.23333

2007.33 9.13333

2007.42 9.16667

2007.50 9.26667

2007.58 9.23333

2007.67 9.23333

2007.75 8.40000

2007.83 9.60000

2007.92 11.9000

2008.00 12.7000

2008.08 13.8500

2008.17 14.0500

2008.25 14.1000

2008.33 13.6500

2008.42 13.4000

2008.50 13.1000

2008.58 13.3000

2008.67 13.6000

2008.75 12.0500

2008.83 11.8500

2008.92 12.2500

2009.00 12.3500

2009.08 12.1000

2009.17 12.1500

2009.25 11.3000

2009.33 11.5000

2009.42 11.9500

2009.50 11.6000

2009.58 11.7500

2009.67 11.5000

2009.75 11.6500

2009.83 12.0000

2009.92 12.9000

2010.00 12.8333

2010.08 12.5750

2010.17 12.5500

2010.25 12.3750

2010.33 12.0500

2010.42 11.9750

2010.50 12.2250

2010.58 12.2750

2010.67 12.2500

2010.75 12.3333

2010.83 11.7000

2010.92 13.1000

2011.00 13.2000

2011.08 13.9000

2011.17 14.4000

2011.25 14.0000

2011.33 13.5000

2011.42 12.9000

2011.50 12.9000

2011.58 13.1000

2011.67 999.000

2011.75 12.3000

2011.83 12.2000

2011.92 12.4500

2012.00 12.4500

2012.08 12.3500

2012.17 12.6000

2012.25 12.3500

2012.33 12.4000

2012.42 12.3500

2012.50 13.1000

2012.58 999.000

2012.67 999.000

2012.75 12.5000

2012.83 12.4333

2012.92 12.1000

2013.00 12.0000

2013.08 11.9000

2013.17 11.6667

2013.25 11.4667

2013.33 11.6000

2013.42 11.7667

2013.50 11.4500

2013.58 11.5000

2013.67 11.5000

2013.75 11.5333

2013.83 11.7000

2013.92 12.1500

2014.00 11.9000

2014.08 12.1000

2014.17 12.1000

2014.25 12.4000

2014.33 12.3500

2014.42 12.2000

2014.50 999.000

2014.58 999.000

2014.67 999.000

2014.75 999.000

2014.83 12.4000

2014.92 12.3000

2015.00 12.7000

2015.08 12.9000

2015.17 13.4000

2015.25 12.7000

2015.33 12.2000

2015.42 12.4000

2015.50 12.4000

2015.58 12.3000

2015.67 12.2000

2015.75 12.2000

2015.83 12.4000

2015.92 12.9750

2016.00 13.3750

2016.08 13.6000

2016.17 13.5000

2016.25 13.7667

2016.33 14.1000

2016.42 14.0667

2016.50 14.4000

2016.58 14.6333

2016.67 14.6667

2016.75 14.5333

2016.83 14.4667

2016.92 14.7000

2017.00 15.3000

2017.08 15.3000

2017.17 14.5000

2017.25 14.0000

2017.33 13.5000

2017.42 13.5000

2017.50 13.8000

2017.58 14.0000

2017.67 14.5000

2017.75 14.6000

2017.83 999.000

2017.92 15.0000

2018.00 15.3000

2018.08 15.4000

2018.17 15.5000

2018.25 15.4000

2018.33 15.1000

2018.42 14.7000

2018.50 15.2000

2018.58 15.9000

2018.67 15.1000

2018.75 15.4000

2018.83 999.000

2018.92 999.000

**3.9 S5.9 Figure 3, bottom panel: all parameters**

A1 - Ekman vertical velocity in the BG region (meters per month)

A2 - Sea ice concentartion in the BG region

A3 - Same as A1 but calculated without geostrophic currents

A4 - Cumullative depth of downwelling (meters)

A5 - same as A1 but from SibSIOM model results

A6 - Error of A1 calculations (meters per month)

**Year A1 A2 A3 A4 A5 A6**

2003.00 -0.124 0.952 -1.218 -0.124 0.257 0.076

2003.08 -0.167 0.974 -4.123 -0.291 -0.144 0.058

2003.17 -0.068 0.977 -2.459 -0.360 -0.554 0.031

2003.25 0.005 0.969 -0.140 -0.355 -0.142 0.041

2003.33 -0.083 0.946 -1.114 -0.438 -0.397 0.048

2003.42 -0.366 0.918 -2.413 -0.804 -0.332 0.066

2003.50 2.214 0.638 4.511 1.410 -0.039 0.408

2003.58 0.250 0.381 -0.179 1.660 -0.131 0.587

2003.67 0.405 0.297 -0.338 2.066 -0.243 0.513

2003.75 -0.658 0.623 0.199 1.408 0.363 0.522

2003.83 0.318 0.866 3.366 1.726 -0.504 0.162

2003.92 -0.810 0.949 -6.533 0.916 -0.731 0.137

2004.00 -0.132 0.929 -3.771 0.784 -0.262 0.109

2004.08 -0.105 0.968 -2.851 0.679 0.166 0.080

2004.17 0.041 0.970 -2.089 0.720 0.015 0.058

2004.25 -0.134 0.980 -2.526 0.586 -0.253 0.035

2004.33 -0.308 0.971 -2.447 0.278 -0.328 0.056

2004.42 -0.444 0.830 -1.793 -0.166 -0.378 0.139

2004.50 -0.392 0.694 -1.180 -0.558 -0.400 0.250

2004.58 -0.385 0.455 -1.382 -0.943 -0.004 0.376

2004.67 0.440 0.401 0.599 -0.503 0.300 0.380

2004.75 -2.918 0.690 -3.718 -3.421 -0.860 0.384

2004.83 -0.832 0.960 -4.260 -4.253 -0.890 0.175

2004.92 -0.206 0.990 -3.027 -4.459 0.092 0.095

2005.00 0.512 0.928 2.478 -3.947 -0.487 0.196

2005.08 -0.030 0.981 -2.360 -3.977 -0.340 0.052

2005.17 -0.198 0.983 -6.146 -4.175 -0.234 0.056

2005.25 -0.009 0.973 -1.466 -4.184 0.074 0.037

2005.33 -0.323 0.925 -3.118 -4.508 -0.915 0.065

2005.42 -0.577 0.868 -3.029 -5.084 -0.302 0.099

2005.50 -0.810 0.731 -2.189 -5.895 0.338 0.156

2005.58 -0.395 0.616 -0.901 -6.290 0.677 0.197

2005.67 -2.999 0.640 -4.677 -9.288 -0.281 0.578

2005.75 -1.546 0.789 -3.470 -10.835 0.214 0.406

2005.83 0.152 0.945 1.337 -10.683 -0.382 0.089

2005.92 -0.099 0.969 -0.864 -10.783 -0.440 0.045

2006.00 0.200 0.932 2.122 -10.583 -0.434 0.075

2006.08 0.204 0.956 2.297 -10.379 -0.431 0.146

2006.17 -0.139 0.957 -2.003 -10.518 -0.554 0.065

2006.25 0.048 0.971 -0.140 -10.470 -0.087 0.025

2006.33 -0.070 0.957 -1.084 -10.540 -0.587 0.043

2006.42 -0.014 0.896 -0.454 -10.554 -0.917 0.044

2006.50 -0.036 0.773 -0.166 -10.590 0.998 0.130

2006.58 -0.348 0.631 -1.071 -10.938 0.105 0.149

2006.67 -0.443 0.618 -1.411 -11.381 -0.175 0.232

2006.75 -1.411 0.747 -1.604 -12.792 -0.076 0.529

2006.83 -0.196 0.944 -2.760 -12.988 0.790 0.089

2006.92 -0.104 0.960 -0.360 -13.092 -1.706 0.078

2007.00 -0.065 0.950 -3.327 -13.157 -0.701 0.031

2007.08 -0.059 0.977 -1.584 -13.215 -0.208 0.061

2007.17 0.061 0.982 -0.834 -13.154 -0.305 0.034

2007.25 -0.190 0.968 -3.783 -13.344 -0.543 0.057

2007.33 -0.032 0.960 -0.881 -13.376 -0.765 0.034

2007.42 -0.762 0.808 -3.467 -14.138 -0.603 0.162

2007.50 -1.449 0.632 -2.823 -15.586 -0.365 0.205

2007.58 -2.062 0.377 -2.919 -17.648 -0.429 0.336

2007.67 -4.282 0.347 -5.049 -21.930 0.178 0.447

2007.75 -3.281 0.609 -5.383 -25.212 -1.674 0.351

2007.83 -1.690 0.892 -6.432 -26.901 -1.218 0.191

2007.92 -0.427 0.953 -3.367 -27.328 -0.760 0.092

2008.00 0.059 0.950 -1.086 -27.269 0.263 0.038

2008.08 0.005 0.973 -1.853 -27.264 -0.368 0.028

2008.17 -0.045 0.982 -4.810 -27.309 -0.757 0.023

2008.25 -0.073 0.969 -0.290 -27.382 -0.244 0.056

2008.33 -0.308 0.944 -3.502 -27.690 -0.805 0.077

2008.42 -0.175 0.808 -1.164 -27.865 -0.953 0.085

2008.50 -0.352 0.560 -0.853 -28.217 -0.614 0.463

2008.58 -0.848 0.286 -1.475 -29.065 -0.238 0.291

2008.67 -1.340 0.240 -2.230 -30.405 -2.050 0.363

2008.75 0.781 0.594 0.291 -29.625 -1.556 0.367

2008.83 -0.337 0.952 -1.750 -29.962 0.228 0.114

2008.92 -0.075 0.955 -2.631 -30.036 -0.033 0.085

2009.00 0.101 0.938 0.049 -29.936 0.023 0.046

2009.08 0.080 0.969 -0.690 -29.855 0.149 0.023

2009.17 -0.105 0.972 -3.982 -29.960 -0.151 0.054

2009.25 -0.041 0.980 -3.236 -30.002 -0.137 0.028

2009.33 0.143 0.925 -0.782 -29.859 -0.372 0.076

2009.42 -0.597 0.953 -3.923 -30.456 -0.194 0.112

2009.50 -0.980 0.850 -3.359 -31.436 -0.064 0.141

2009.58 -0.015 0.550 0.163 -31.451 -0.320 0.389

2009.67 -0.626 0.358 -1.145 -32.077 -0.413 0.510

2009.75 -1.955 0.697 -2.898 -34.032 -0.902 0.391

2009.83 -0.560 0.944 -1.666 -34.592 -0.778 0.165

2009.92 -0.076 0.988 -2.114 -34.668 -0.378 0.053

2010.00 0.096 0.958 -0.430 -34.572 -0.325 0.034

2010.08 -0.001 0.989 -1.054 -34.572 -0.440 0.043

2010.17 0.098 0.991 0.940 -34.475 -0.434 0.027

2010.25 0.031 0.991 -0.534 -34.443 -1.049 0.020

2010.33 -0.091 0.988 -3.529 -34.535 -0.462 0.021

2010.42 -0.516 0.940 -3.265 -35.051 -1.115 0.087

2010.50 -0.079 0.792 -0.459 -35.129 -0.926 0.153

2010.58 -1.297 0.536 -2.539 -36.426 -1.276 0.242

2010.67 -0.466 0.298 -0.382 -36.892 -2.351 0.766

2010.75 -2.331 0.700 -3.802 -39.222 -3.004 0.348

2010.83 -0.354 0.931 -0.726 -39.576 -2.520 0.179

2010.92 -0.056 0.989 -1.377 -39.632 -1.153 0.030

2011.00 0.044 0.958 -1.506 -39.588 -0.321 0.023

2011.08 0.083 0.990 0.595 -39.505 -0.361 0.025

2011.17 0.053 0.991 -0.780 -39.452 -0.212 0.014

2011.25 0.035 0.990 -2.750 -39.417 -0.170 0.019

2011.33 -0.050 0.979 -1.422 -39.467 -0.804 0.024

2011.42 -0.496 0.953 -4.412 -39.963 -0.257 0.079

2011.50 -0.649 0.783 -1.173 -40.612 -0.495 0.152

2011.58 -1.025 0.451 -0.822 -41.637 -0.119 0.280

2011.67 -1.712 0.281 -2.861 -43.349 -1.075 0.433

2011.75 -2.138 0.602 -3.459 -45.486 -0.192 0.499

2011.83 -0.278 0.857 -1.484 -45.764 -0.839 0.293

2011.92 -0.104 0.986 -2.117 -45.868 -0.577 0.040

2012.00 0.136 0.947 0.056 -45.733 -0.354 0.030

2012.08 0.028 0.984 -1.315 -45.705 0.071 0.019

2012.17 0.116 0.981 -0.755 -45.589 -0.160 0.020

2012.25 -0.030 0.983 -2.608 -45.619 -0.791 0.020

2012.33 -0.039 0.955 -1.555 -45.658 -0.218 0.024

2012.42 -0.252 0.832 -2.696 -45.909 -1.087 0.148

2012.50 -0.493 0.579 -1.452 -46.402 -1.127 0.198

2012.58 1.206 0.345 1.532 -45.196 0.223 0.420

2012.67 1.658 0.071 1.883 -43.538 -0.562 0.644

2012.75 0.679 0.290 0.653 -42.859 -1.318 0.388

2012.83 0.249 0.844 -0.321 -42.610 -0.970 0.152

2012.92 -0.002 0.977 -0.438 -42.612 -0.686 0.037

2013.00 -0.122 0.955 -6.053 -42.734 -0.294 0.039

2013.08 -0.041 0.995 -4.430 -42.775 -0.045 0.027

2013.17 -0.156 0.985 -5.607 -42.931 0.186 0.055

2013.25 0.040 0.989 -1.030 -42.891 -0.120 0.028

2013.33 0.029 0.978 0.107 -42.862 -0.814 0.026

2013.42 -0.050 0.953 -1.986 -42.912 -0.965 0.036

2013.50 -0.067 0.818 -1.132 -42.979 -0.015 0.239

2013.58 -0.354 0.667 -1.755 -43.333 -0.791 0.170

2013.67 -1.486 0.680 -1.859 -44.819 -0.084 0.305

2013.75 -0.368 0.788 -0.347 -45.187 -1.559 0.225

2013.83 0.131 0.936 -0.706 -45.056 -0.142 0.185

2013.92 -0.053 0.977 -2.211 -45.108 -0.236 0.071

2014.00 -0.181 0.951 -3.573 -45.290 -0.041 0.082

2014.08 0.000 0.988 -3.261 -45.290 -0.010 0.028

2014.17 0.051 0.992 -1.463 -45.238 -0.122 0.017

2014.25 -0.056 0.993 -3.033 -45.294 -0.241 0.028

2014.33 0.029 0.976 -0.487 -45.265 -0.474 0.038

2014.42 -0.325 0.949 -2.680 -45.590 -1.438 0.078

2014.50 0.004 0.786 0.323 -45.587 -0.480 0.163

2014.58 -1.342 0.669 -2.291 -46.929 -0.412 0.352

2014.67 -0.989 0.516 -1.805 -47.918 -1.182 0.568

2014.75 -1.150 0.745 -1.061 -49.068 -2.163 0.544

2014.83 -0.340 0.936 -4.125 -49.407 -0.356 0.135

2014.92 -0.086 0.988 -2.244 -49.494 -0.320 0.037

2015.00 0.053 0.952 -1.959 -49.441 -0.477 0.042

2015.08 0.081 0.982 -1.562 -49.359 -0.031 0.039

2015.17 0.007 0.985 -2.068 -49.353 0.098 0.032

2015.25 -0.123 0.990 -4.296 -49.475 -0.104 0.039

2015.33 -0.140 0.958 -1.809 -49.615 -0.270 0.061

2015.42 0.059 0.885 -0.695 -49.557 -0.843 0.078

2015.50 -0.385 0.677 -0.714 -49.941 -0.284 0.166

2015.58 0.593 0.426 1.639 -49.348 0.592 0.568

2015.67 -1.366 0.334 -2.043 -50.714 0.664 0.464

2015.75 -1.092 0.690 -3.858 -51.806 0.442 0.378

2015.83 -0.490 0.967 -3.313 -52.297 -0.283 0.124

2015.92 -0.064 0.987 -1.652 -52.361 0.130 0.041

2016.00 -0.070 0.958 -3.856 -52.431 -1.069 0.025

2016.08 -0.333 0.995 -8.980 -52.765 -0.278 0.041

2016.17 -0.287 0.993 -6.766 -53.051 -1.092 0.063

2016.25 -0.675 0.985 -9.904 -53.726 -0.448 0.073

2016.33 -0.468 0.916 -2.162 -54.195 -0.197 0.090

2016.42 0.173 0.825 -0.060 -54.022 -0.637 0.117

2016.50 0.172 0.621 0.005 -53.850 -0.251 0.272

2016.58 -1.569 0.372 -2.984 -55.419 -0.454 0.391

2016.67 1.600 0.200 1.870 -53.818 -0.727 0.719

2016.75 -1.350 0.434 -2.082 -55.169 -0.357 0.455

2016.83 -0.413 0.822 -0.432 -55.582 -0.473 0.287

2016.92 -0.048 0.988 -1.168 -55.630 -0.345 0.049

2017.00 0.197 0.950 1.106 -55.433 -0.606 0.066

2017.08 0.003 0.990 -0.012 -55.430 -0.373 0.030

2017.17 0.079 0.992 2.002 -55.351 -0.131 0.016

2017.25 -0.225 0.994 -5.073 -55.576 -0.843 0.036

2017.33 -0.153 0.968 -0.639 -55.729 -0.082 0.042

2017.42 -0.483 0.889 -3.398 -56.212 -0.946 0.114

2017.50 -0.530 0.675 -1.892 -56.742 0.079 0.279

2017.58 -0.467 0.281 -0.725 -57.209 -0.940 0.431

2017.67 -1.109 0.205 -1.325 -58.317 -1.110 0.722

2017.75 -0.601 0.492 0.292 -58.918 -0.877 0.494

2017.83 -0.979 0.831 -1.013 -59.897 -1.501 0.588

2017.92 -0.580 0.978 -5.404 -60.477 0.025 0.126

2018.00 -0.066 0.950 -0.608 -60.543 -0.486 0.043

2018.08 -0.003 0.990 -1.938 -60.546 -0.121 0.028

2018.17 -0.081 0.992 -2.949 -60.627 0.048 0.022

2018.25 -0.168 0.994 -3.022 -60.795 -0.653 0.024

2018.33 -0.350 0.968 -4.386 -61.144 -0.611 0.055

2018.42 -0.143 0.889 -0.225 -61.287 -0.350 0.163

2018.50 0.919 0.675 1.191 -60.368 -0.234 0.274

2018.58 -0.188 0.281 -0.737 -60.556 0.307 0.505

2018.67 -2.165 0.205 -4.147 -62.721 -0.794 0.452

2018.75 -2.225 0.492 -3.115 -64.946 -1.590 0.553

2018.83 -1.239 0.831 -3.298 -66.186 -0.968 0.335

2018.92 -0.177 1.011 -0.269 -66.363 -0.387 0.084
